# Supplementary material for: The regulatory relationship between NAMPT and PD-L1 in cancer and identification of a dual-targeting inhibitor
Source: EMBO Mol Med. 2024 Mar 6;16(4):885–903. doi: 10.1038/s44321-024-00051-z (PMC11018795; doi:10.1038/s44321-024-00051-z)
Supplement: Supplementary file 1 — Appendix [file 44321_2024_51_MOESM1_ESM.pdf]

Appendix

The regulatory relationship between NAMPT and PD-L1 in cancer and identification of a dual-targeting inhibitor

Yuan Yang[1,2#], Zefei Li[3#], Yidong Wang[1,2], Jiwei Gao[1,2], Yangyang Meng[3], Simeng Wang[1,2], Xiaoyao zhao[1,2],Chengfang Tang[1,2],Weiming Yang[1,2],Yingjia Li[1,2], Jie Bao[4], Xinyu Fan[5], Jing Tang[4], Jingyu Yang[1,2], Chunfu Wu[1,2], Mingze Qin[3]*, Lihui Wang[1,2]*

*** Corresponding author.**

E-mail addresses: lhwang@syphu.edu.cn (LH Wang); qinmingze@syphu.edu.cn (MZ Qin).

This PDF file includes:

Appendix Figure S1: Page 2

Appendix Figure S2: Page 3

Appendix Figure S3: Page 4

Appendix Figure S4: Page 5

Appendix Figure S5: Page 6

Appendix Figure S6: Page 7

Appendix Figure S7: Page 8

Appendix Figure S8: Page 9

Appendix Table S1: Page 11

Appendix Figure S1.

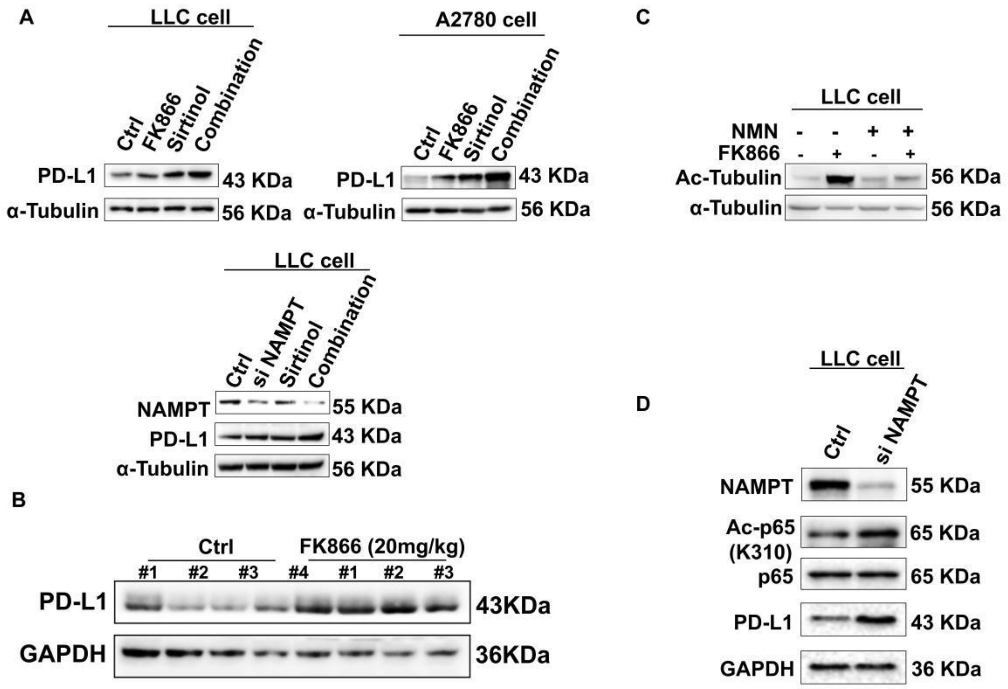

(A) PD-L1 protein expression was detected by western blot after adding FK866, Sirtinol, and combination for 72 h to A2780 and LLC cells. (A2780: FK866 0.5 μM, Sirtinol 10 μM; LLC: FK866 2 μM, Sirtinol 10 μM). Western blot analyzed NAMPT and PD-L1 expression after using ctrl, si NAMPT, Sirtinol, or the Combination in LLC cells. **(B)** Relative expression of PD-L1 after adding FK866 (20 mg/kg) from tumor model in which mice were inoculated with LLC cells with double over-expression of NAMPT and PD-L1 (n = 4 mice/group). **(C)** Ac-tubulin protein expression was detected after adding FK866 and NMN for 72 h to LLC cells by western blot. (LLC: FK866 2 μM, NMN 1 mM). **(D)** NAMPT, Ac-p65 (K310), p65, and PD-L1 protein expression were detected by western blot after using si Vector and si NAMPT in LLC cells for 72 h.

Appendix Figure S2.

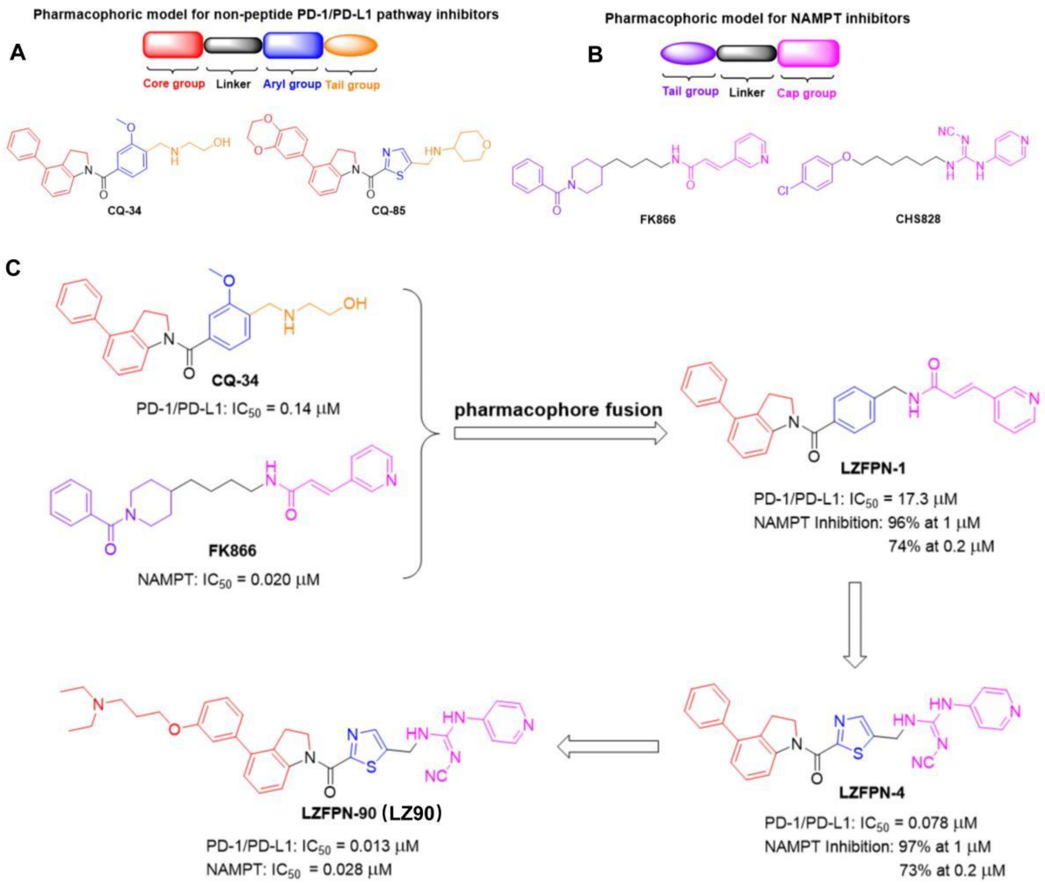

D

Table 1: The influence of LZFPN-90 to NAMPT enzyme activity

LZFPN-90	NAMPT Fluorescence		% Activity	
	Repeat1	Repeat2	Repeat1	Repeat2
No compound	2231	2265		
**4 nM**	2114	2099	94	93
**8 nM**	1758	1642	77	71
**16 nM**	1359	1265	58	53
**31 nM**	1228	1142	51	47
**63 nM**	749	784	29	30
**125 nM**	702	632	26	23
**250 nM**	544	456	19	15
**500 nM**	244	221	5	4
**1000 nM**	195	201	2	3
Background	134	157		

Table 2: NAMPT enzyme results table

	Repeat 1 (nM)	Repeat 2 (nM)	Average (nM)	SD
LZFPN-90	30.2	25.3	27.8	3.5
FK866	22.9	16.8	19.9	4.3

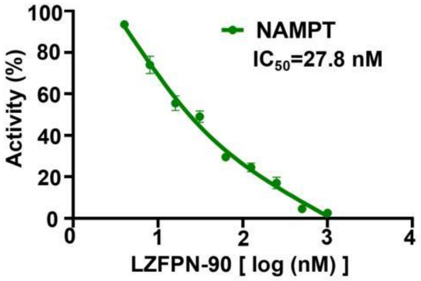

(A-B) Pharmacophoric model and chemical structures of the non-peptide PD-1/PD-L1 (A) and NAMPT (B) inhibitors. **(C)** Design strategy and structural optimization of dual inhibitors targeting the PD-1/PD-L1 interaction and NAMPT. **(D)** Table 1: The influence of LZ90 on NAMPT enzyme activity (left). Table 2: NAMPT enzyme results table and the NAMPT activity curves of different concentrations of LZ90 (right). Data are shown as mean ± SEM.

Appendix Figure S3.

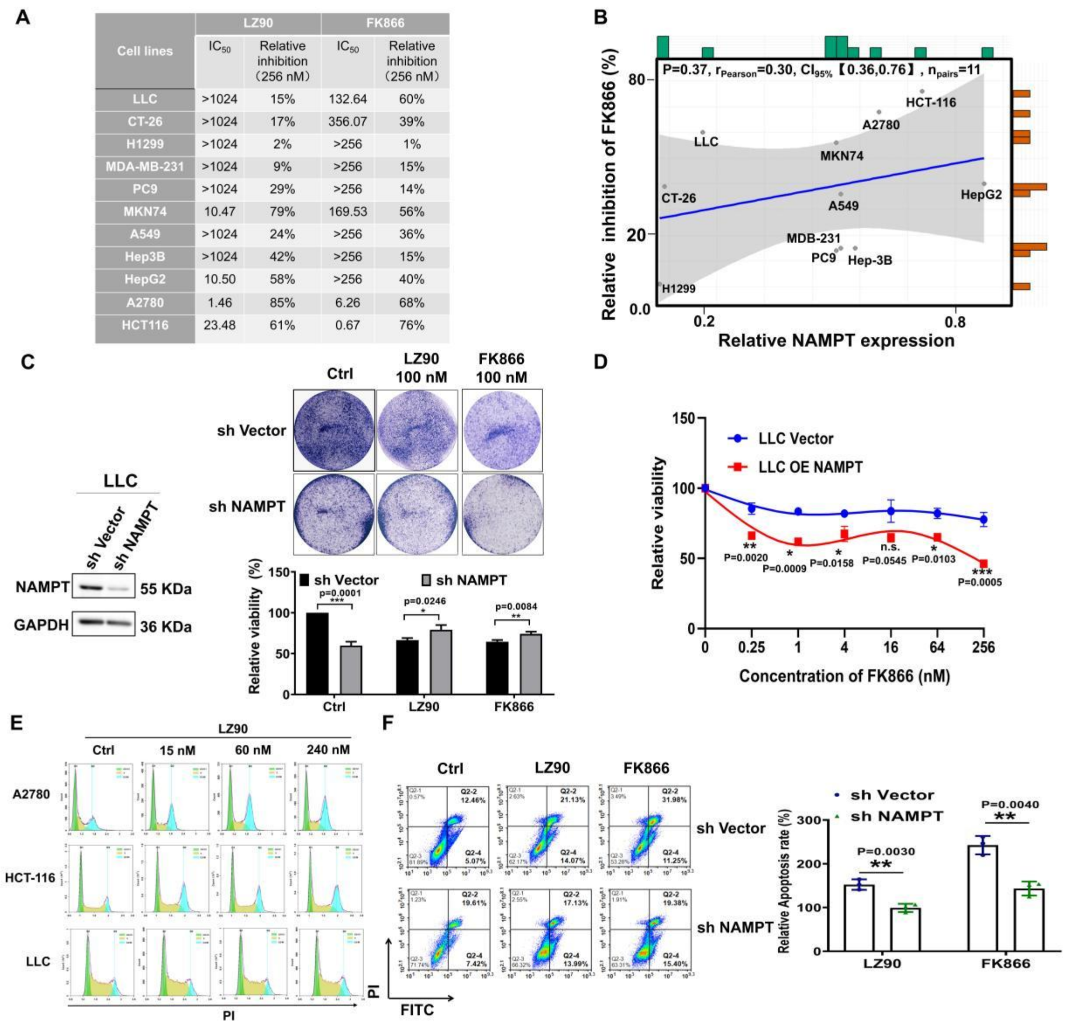

Cell lines	LZ90		FK866	
	IC_{50}	Relative inhibition (256 nM)	IC_{50}	Relative inhibition (256 nM)
LLC	>1024	15%	132.64	60%
CT-26	>1024	17%	356.07	39%
H1299	>1024	2%	>256	1%
MDA-MB-231	>1024	9%	>256	15%
PC9	>1024	29%	>256	14%
MKN74	10.47	79%	169.53	56%
A549	>1024	24%	>256	36%
Hep3B	>1024	42%	>256	15%
HepG2	10.50	58%	>256	40%
A2780	1.46	85%	6.26	68%
HCT116	23.48	61%	0.67	76%

(A) Detecting the sensitivity of different cell lines to LZ90 and FK866 by MTT assay for 48 h. **(B)** Correlation analysis between the inhibitory activity of FK866 and the protein expression of NAMPT in 11 cell lines. **(C)** NAMPT was knocked down by sh RNA in the LLC cells and verified by Western blot. In LLC sh Vector and sh NAMPT cells, after three days of treatment with LZ90 (100 nM) and FK866 (100 nM), cell proliferation was detected by cloning assay(n = 3 per group). **(D)** The inhibitory activity of the NAMPT inhibitor FK866 on the viability of LLC Vector and LLC OE NAMPT cells (n = 3 per group). **(E)** Effects of LZ90 on the cell cycle of A2780, HCT116, and LLC cells. **(F)** In LLC sh Vector and sh NAMPT cells, the apoptosis was detected by flow cytometry after three days of treatment with LZ90 (100 nM) and FK866 (100 nM) (n = 3 per group). Data information: Data represent different numbers (n) of biological replicates. Data information: Data are shown as mean ± SEM. Two-tailed Student’s t-test is used in (C, D, E). *P < 0.05, **P < 0.01, ***P < 0.001, n.s., not significant, compared with control.

Appendix Figure S4.

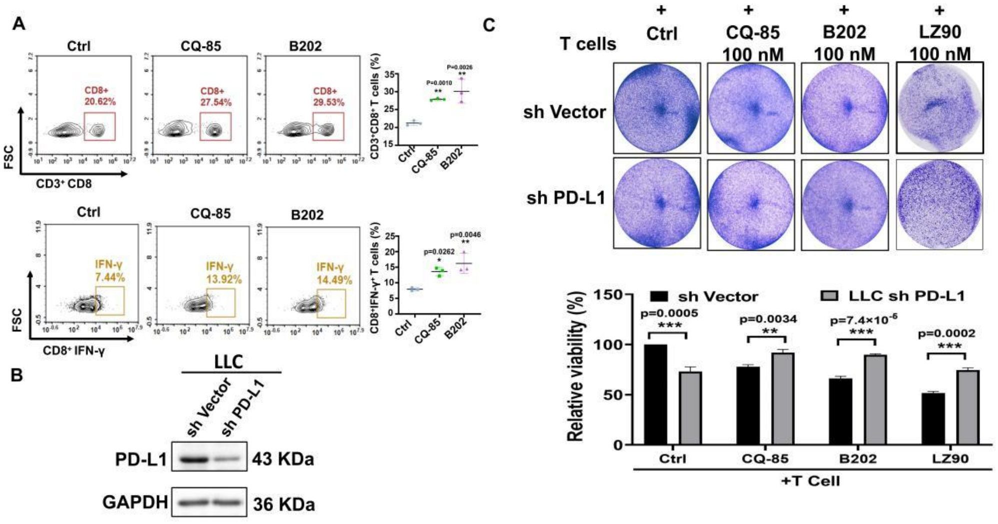

(A) The proportion and representative staining of CD8$^+$ T cells and IFN-γ$^+$ of CD8$^+$ T cells treatment of CQ-85 (100 nM), BMS202 (100 nM) after co-culture T cells with LLC cells(n = 3 mice/group). **(B)** PD-L1 was knocked down by sh RNA in the LLC cells and verified by Western blot. **(C)** With T cells, the LLC sh Vector and LLC sh PD-L1 cells were treated with CQ-85 (100 nM), BMS202 (100 nM), and LZFPN-90 (100 nM) for 72 h, the killing of T cells was detected (n = 3 per group). Data information: Data are shown as mean ± SEM. One-way ANOVA followed by Bonferroni's test is used in (A). Two-tailed Student's t-test is used in (C). *P < 0.05, **P < 0.01, ***P < 0.001 compared with control. n.s., not significant.

Appendix Figure S5.

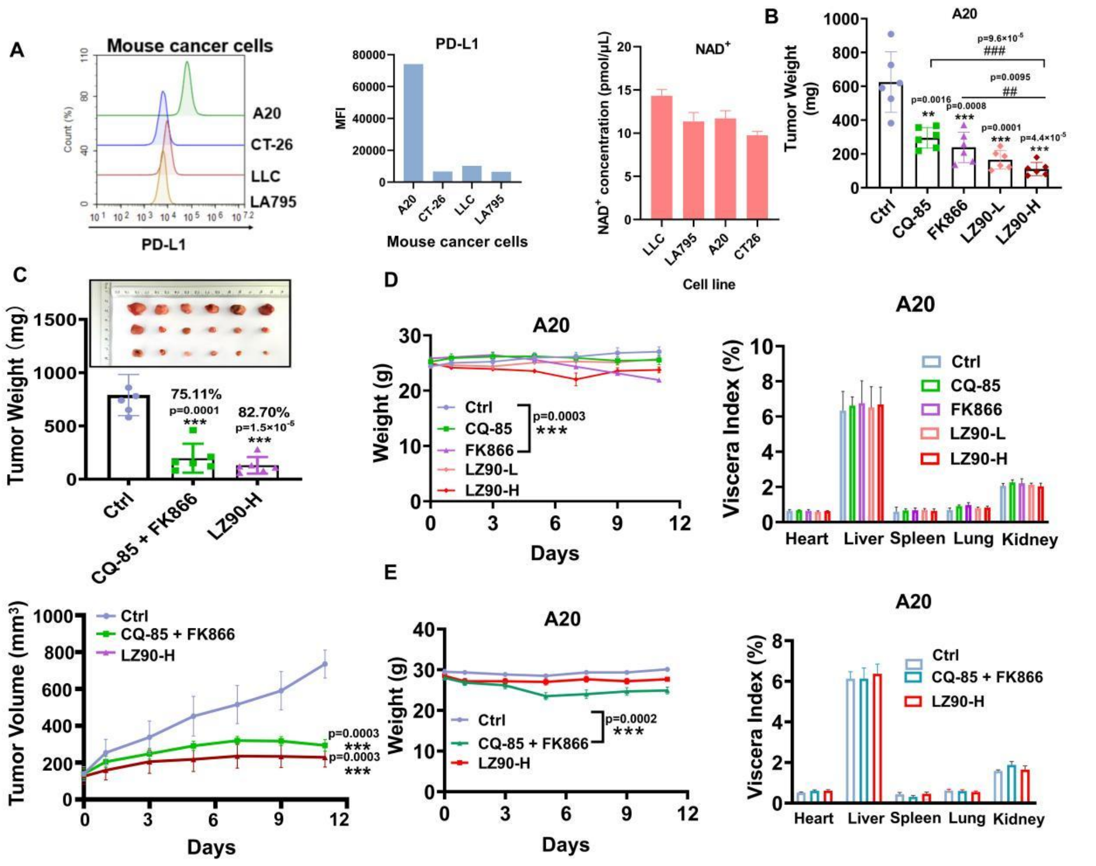

(A) The membrane expression of PD-L1 (n = 1 per group) and the NAD$^+$ content (n = 3 per group) was quantified in the mice cell lines A20, CT-26, LLC, and LA795. **(B)** Tumor weight and tumor volume in the A20 mice model. Balb/c mice bearing A20-derived tumors were treated with Ctrl, CQ-85 (20 mg/kg, i.p.), FK866 (20 mg/kg, i.p.), and LZ90 (20 mg/kg, 40 mg/kg, i.p.), n = 6 mice/ group. **(C)** Tumor weight and tumor growth curves in A20 tumor-bearing Balb/c mice were treated with a combination of CQ-85 (20 mg/kg, i.p.) and FK866 (20 mg/kg, i.p.), LZ90 (40 mg/kg, i.p.), n = 6 mice/group. **(D)** Mice body weight and organ index of A20 model mice. Balb/c mice bearing A20-derived tumors were treated with Ctrl, CQ-85 (20 mg/kg, i.p.), FK866 (20 mg/kg, i.p.), and LZ90 (20 mg/kg, 40 mg/kg, i.p.), n = 6 mice/group. **(E)** Mice body weight and organ index of A20 model mice. A20 tumor-bearing Balb/c mice were treated with CQ-85 (20 mg/kg, i.p.) and FK866 (20 mg/kg, i.p.), and LZ90 (40 mg/kg, i.p.), n = 6 mice/group. Data information: Data are shown as mean ± SEM. One-way ANOVA followed by Bonferroni's test is used in (B, C). Two-tailed Student's t-test is used in (B, D, E). **P < 0.01, ***P < 0.001 compared with control; $^{##}$ P< 0.01， $^{###}$ P< 0.001, compared with LZ90-H group; n.s., not significant.

Appendix Figure S6.

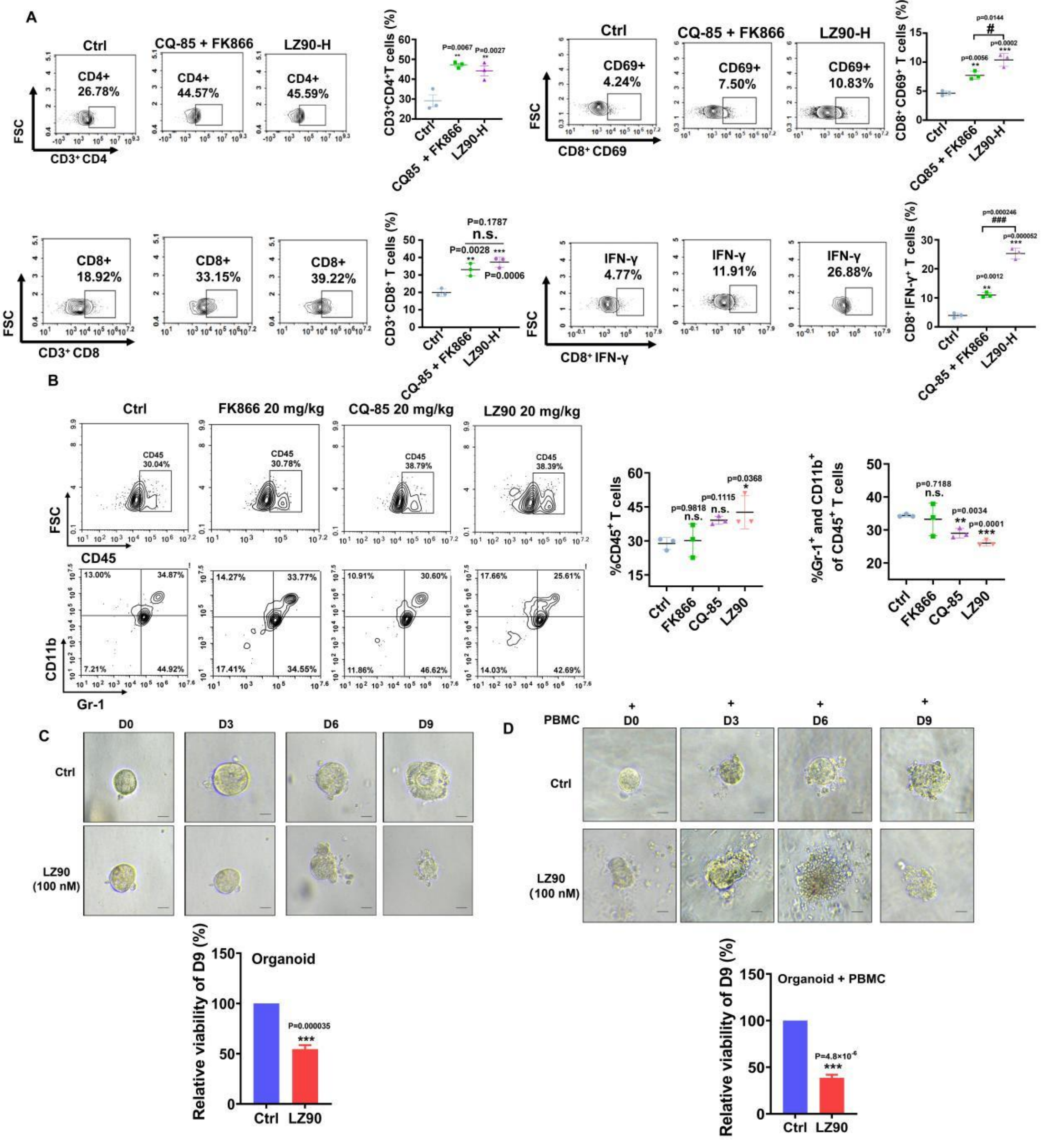

(A). Flow cytometry was used to analyze the proportion of $CD4^+$ and $CD69^+$ in the tumors from the A20 mice model, treating with CQ-85 and FK866 combination (20 mg/kg each, i.p.), LZ90 (40 mg/kg, i.p.), n = 3 mice/group. **(B).** Flow cytometry was used to analyze the proportion of $CD45^+$ cells and MDSCs in the tumors from the A20 mice model, n = 3 mice/group. **(C)** Representative fluorescent images of PBMC infiltration during co-culture for 3, 6, and 9 days (scale bar 100 μm)**.** The picture of the organoid proliferation on the 9th day was placed in the text Fig 6G as a representative pictur**e** (n = 3 per group). **(D)** representative bright-field images of PBMC co-cultured with organoids for 3, 6, and 9 days after treating with LZ90 (100 nM) (scale bar 100 μm),n = 3 per group. Data information: Data are shown as mean ± SEM. One-way ANOVA followed by Bonferroni's test is used in (A, B). Two-tailed Student's t-test is used in (C, D). *P < 0.05, **P < 0.01,***P < 0.001 compared with control; $^{\#}$ P< 0.05, $^{\#\#\#}$ P< 0.001 compared with LZ90-H group; n.s., not significant.

Appendix Figure S7.

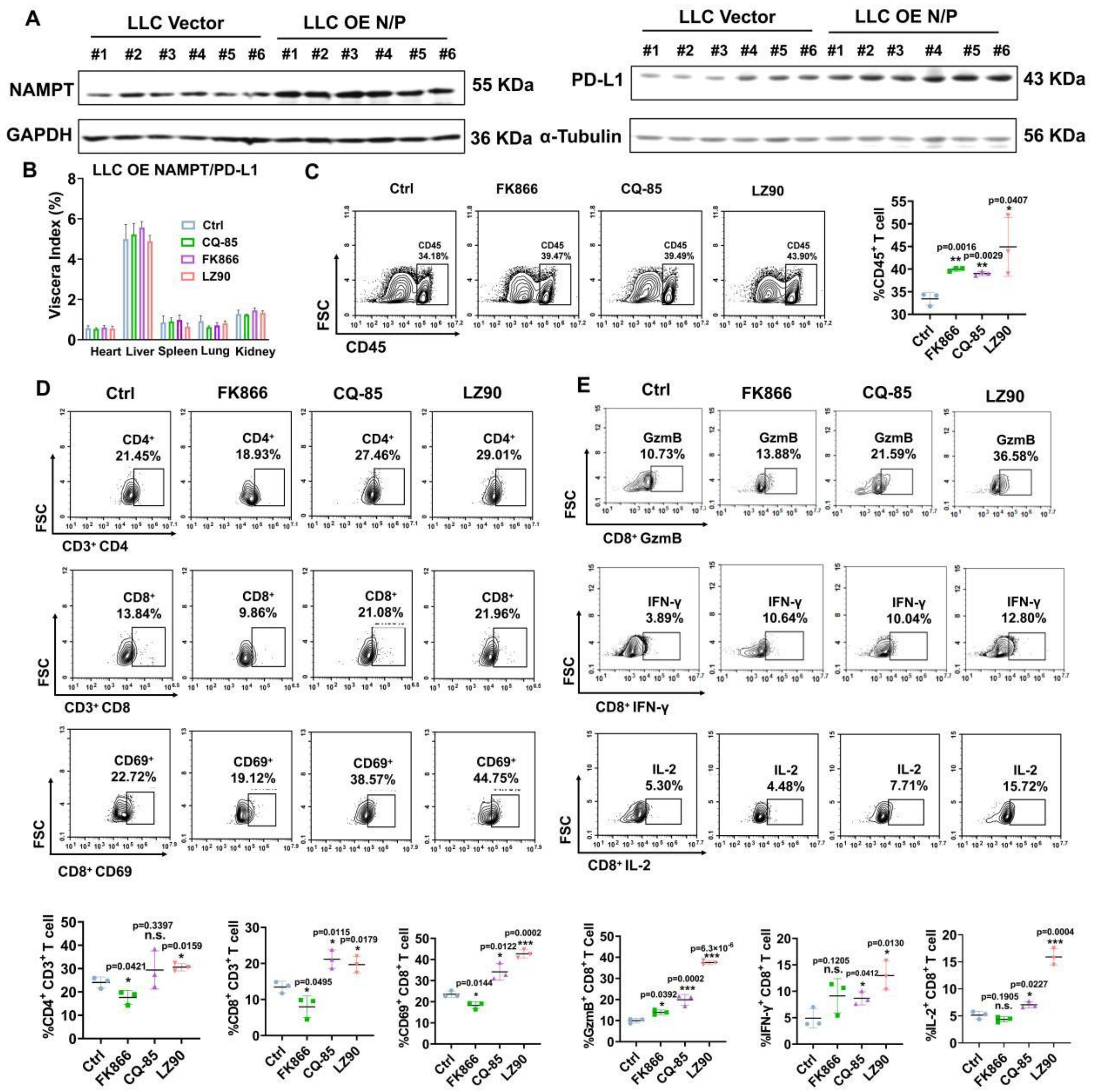

(A) The overexpression of NAMPT and PD-L1 was verified in tumor tissues of LLC OE NAMPT/PD-L1 bearing mice by Western blot, n = 6 mice/group. **(B)** Organ index in the LLC OE NAMPT/PD-L1 mice model. C57BL/6 mice bearing tumors derived from LLC OE NAMPT/PD-L1 cells were treated with Ctrl, CQ-85 (20 mg/kg, i.p.), FK866 (20 mg/kg, i.p.), and LZ90 (20 mg/kg, i.p.), n = 6 mice/per group. **(C)** Flow cytometry was used to detect the proportion of $CD45^{+}$ cells in the tumors from the LLC OE NAMPT/PD-L1 mice model, n = 3 mice/group. **(D)** Flow cytometry was used to detect the proportion of $CD4^{+}$, $CD8^{+,}$ and $CD69^{+}$ cells in tumors from the LLC OE NAMPT/PD-L1 mice model, n = 3 mice/group. **(E)** Flow cytometry was used to detect the content of GzmB, IFN-γ, and IL-2 in $CD8^{+}$ T cells from the LLC OE NAMPT/PD-L1 mice model, n = 3 mice/group. Data information: Data are shown as mean ± SEM. One-way ANOVA followed by Bonferroni's test is used in (C, D, E). *$P < 0.05$, **$P < 0.01$, ***$P < 0.001$ compared with control; n.s., not significant.

Appendix Figure S8.

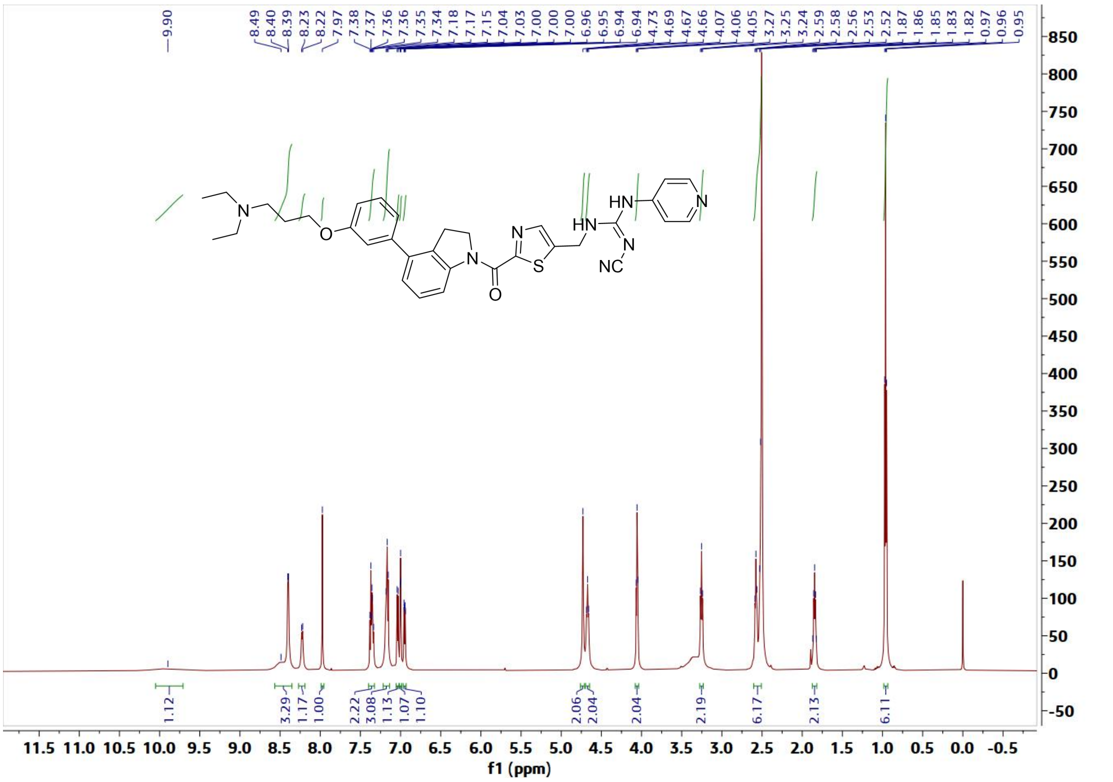

A. ^{1}H NMR spectrum of LZFPN-90

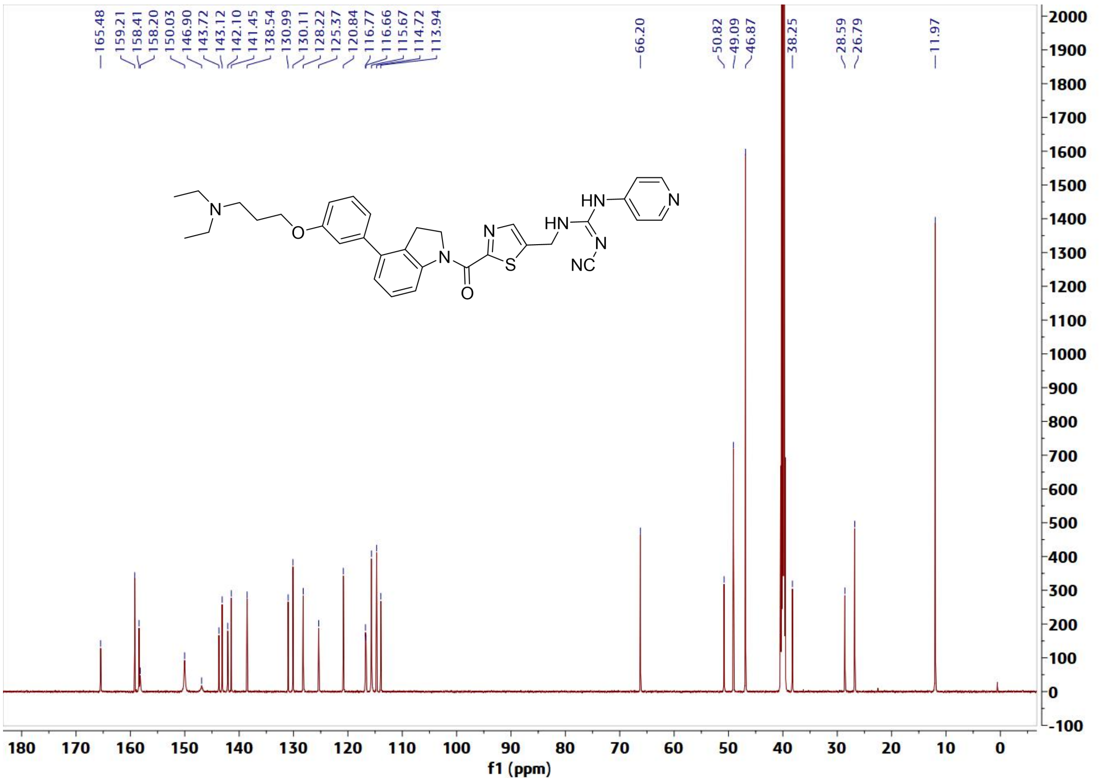

B. ^{13}C NMR spectrum of LZFPN-90

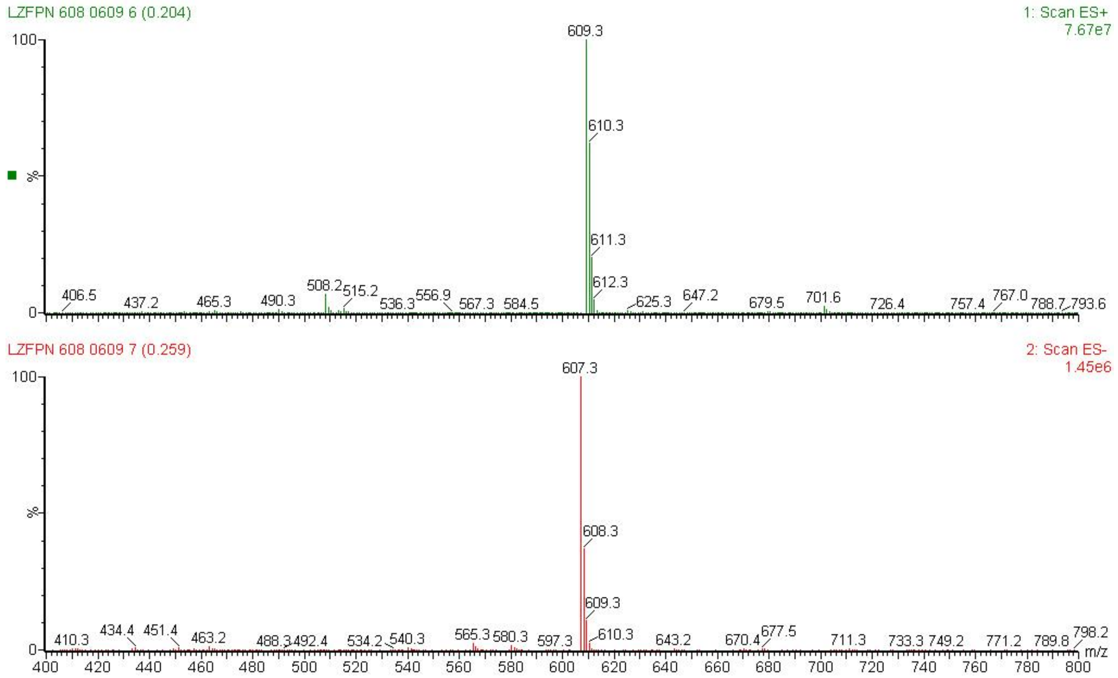

C. MS spectrum of LZFPN-90

(A). ^{1}H NMR spectrum of LZFPN-90**. (B).** ^{13}C NMR spectrum of LZFPN-90. **(C).**MS spectrum of LZFPN-90

Appendix Table S1

Table S1 Primers Sequence Table				
**Gene**	**Species**	**Forward**	**Reverse**	**Usage**
CD274	human	CTGCACTTTTAGGAGATTAGATC	CTGCACTTTTAGGAGATTAGATC	qPCR
CD274	mouse	GTGAAACCCTGAGTCTTATCC	GACCATTCTGAGACAATTCC	qPCR
Gapdh	human	GACCCCTTCATTGACCTCAAC	CTTCTCCATGGTGGTGAAGA	qPCR
Gapdh	mouse	TGACCTCAACTACATGGTCTACA	CTTCCCATTCTCGGCCTTG	qPCR
β-actin	human	CCTTCCTGGGCATGGAGTCCT	GGAGCAATGATCTTGATCTTC	qPCR
β-actin	mouse	GTGACGTTGACATCCGTAAAGA	GCCGGACTCATCGTACTCC	qPCR
Aldoa	human	ATGCCCTACCAATATCCAGCA	GCTCCCAGTGGACTCATCTG	qPCR
Aldoa	mouse	CGTGTGAATCCCTGCATTGG	CAGCCCCTGGGTAGTTGTC	qPCR
Eno1	human	TGGTGTCTATCGAAGATCCCTT	CCTTGGCGATCCTCTTTGG	qPCR
Eno1	mouse	TGCGTCCACTGGCATCTAC	CAGAGCAGGCGCAATAGTTTTA	qPCR
Pfkl	human	GCTGGGCGGCACTATCATT	TCAGGTGCGAGTAGGTCCG	qPCR
Pfkl	mouse	GGAGGCGAGAACATCAAGCC	CGGCCTTCCCTCGTAGTGA	qPCR
Hk2	human	GGCTTGGAGCCACCACTCACC	CCTTCTGGAGCCCATTGTCCGT	qPCR
Hk2	mouse	TGCAGAAGGTTGACCAGTATCTCTA	GTCCAGAGCCAGGAACTCG	qPCR
Glut1	human	CTCATCGCCCAGGTGTTC	TTCTCCTCGTTGCGGTTG	qPCR
Glut1	mouse	GGCCTAAGGTCACATGAAGAAGG	AGCGGTGGTTCCATGTTTGA	qPCR
1-P65（P1）	human	TGGCTGAAGGGTAGAAACAGGT	CTCCTAGATGGCCTGGATGATC	CHIP-qPCR
2-P65 （P2）	human	GCCAGGATTAAATCATATCCTCCTAG	TGAATGGCTGAAGGGTAGAAACAG	CHIP-qPCR
3-P65 （P3）	human	AATAGGGTTTGGGCCCAGC	CATTAAATGAAAATATCAGAGGGCATTGC	CHIP-qPCR
