# Supplementary figures and images for: The regulatory relationship between NAMPT and PD-L1 in cancer and identification of a dual-targeting inhibitor
Source: EMBO Mol Med. 2024 Mar 6;16(4):885–903. doi: 10.1038/s44321-024-00051-z (PMC11018795; doi:10.1038/s44321-024-00051-z)

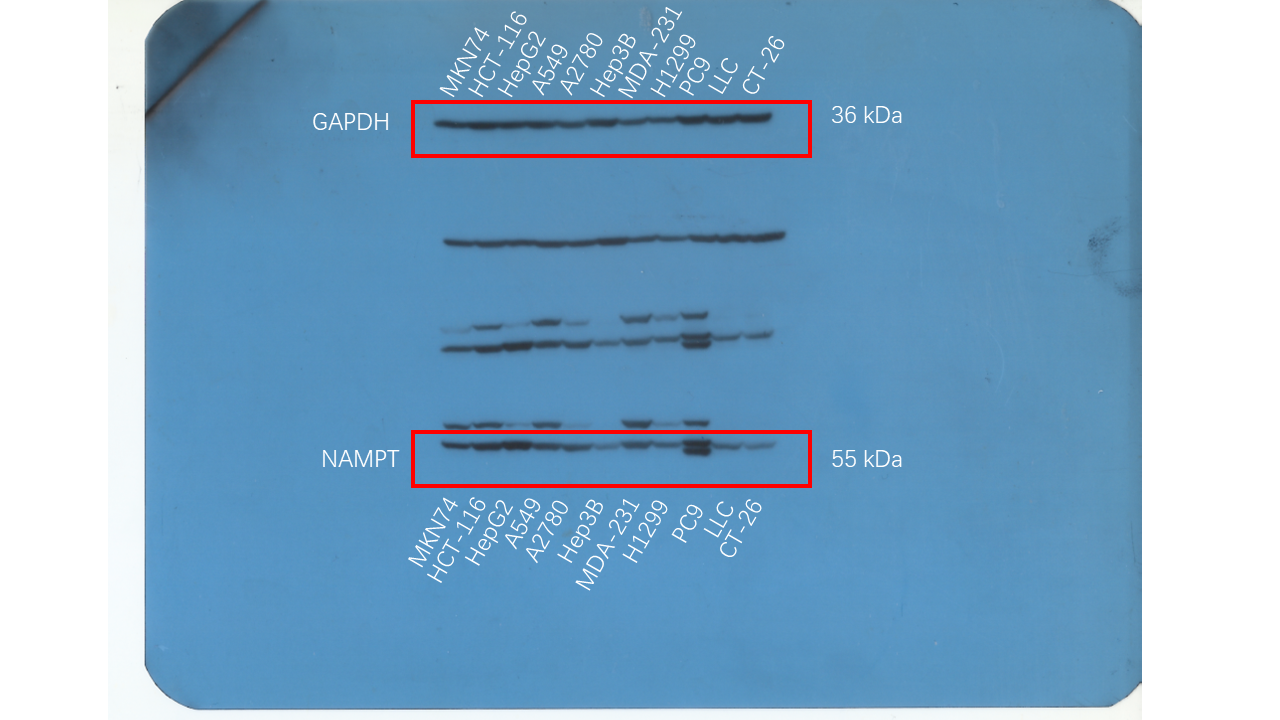

Supplement: Supplementary file 2 — Source Data Fig. 1 [file 44321_2024_51_MOESM2_ESM.zip › Fig-1/1C/Western Blot-Expression of NAMPT in different cell lines.tif]

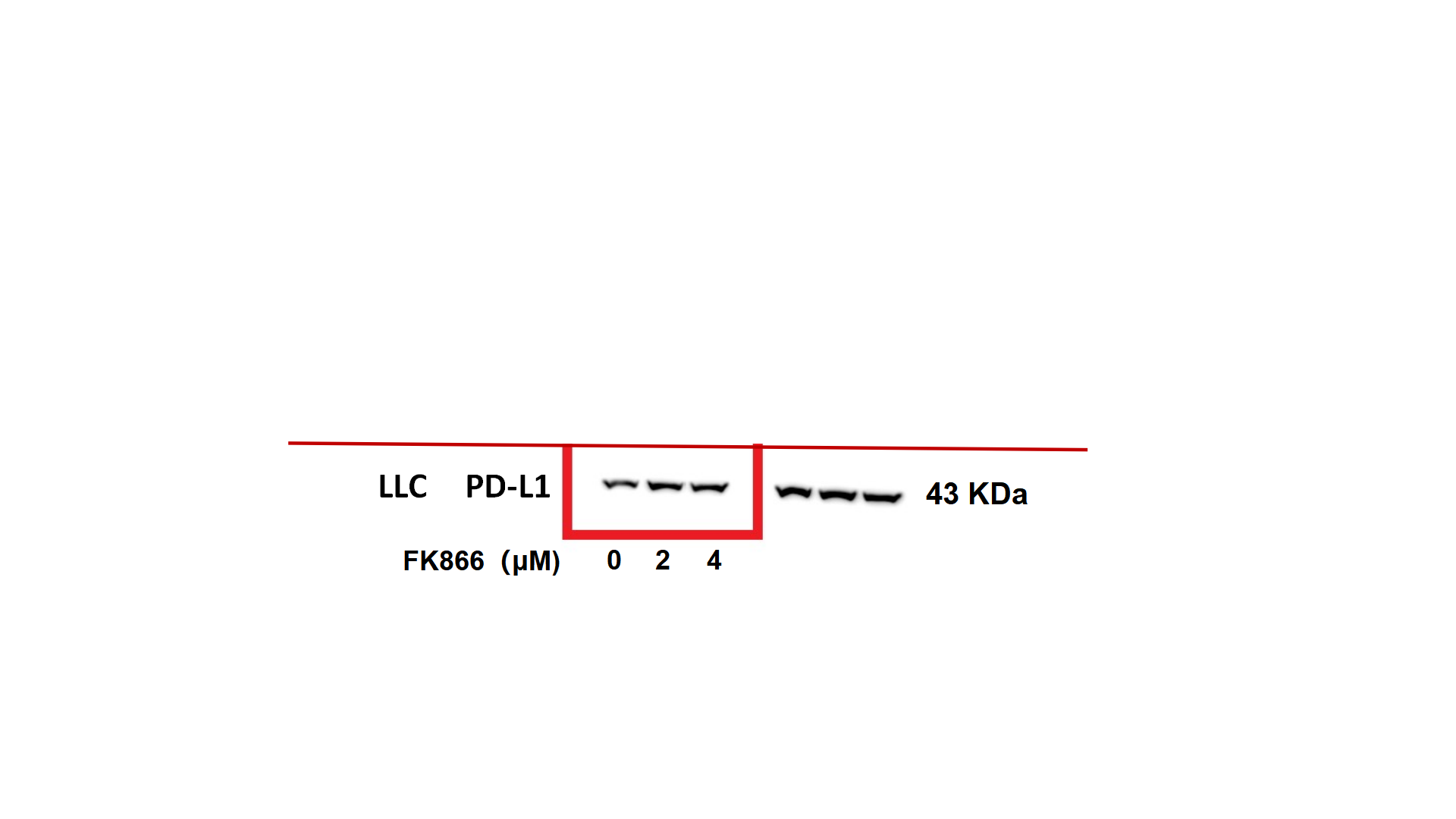

Supplement: Supplementary file 2 — Source Data Fig. 1 [file 44321_2024_51_MOESM2_ESM.zip › Fig-1/1D/LLC PD-L1.tif]

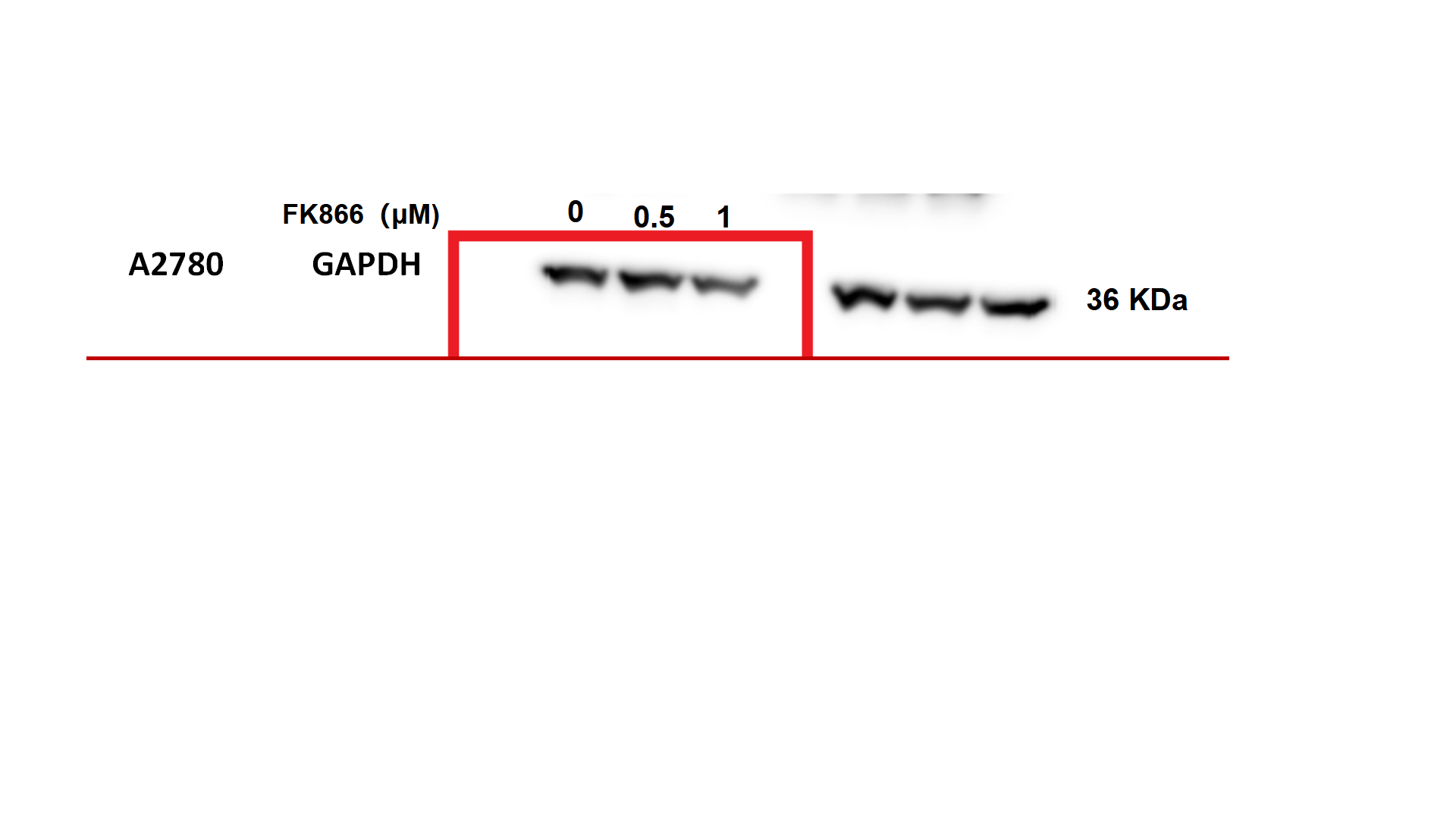

Supplement: Supplementary file 2 — Source Data Fig. 1 [file 44321_2024_51_MOESM2_ESM.zip › Fig-1/1D/Western Blot-A2780 GAPDH.tif]

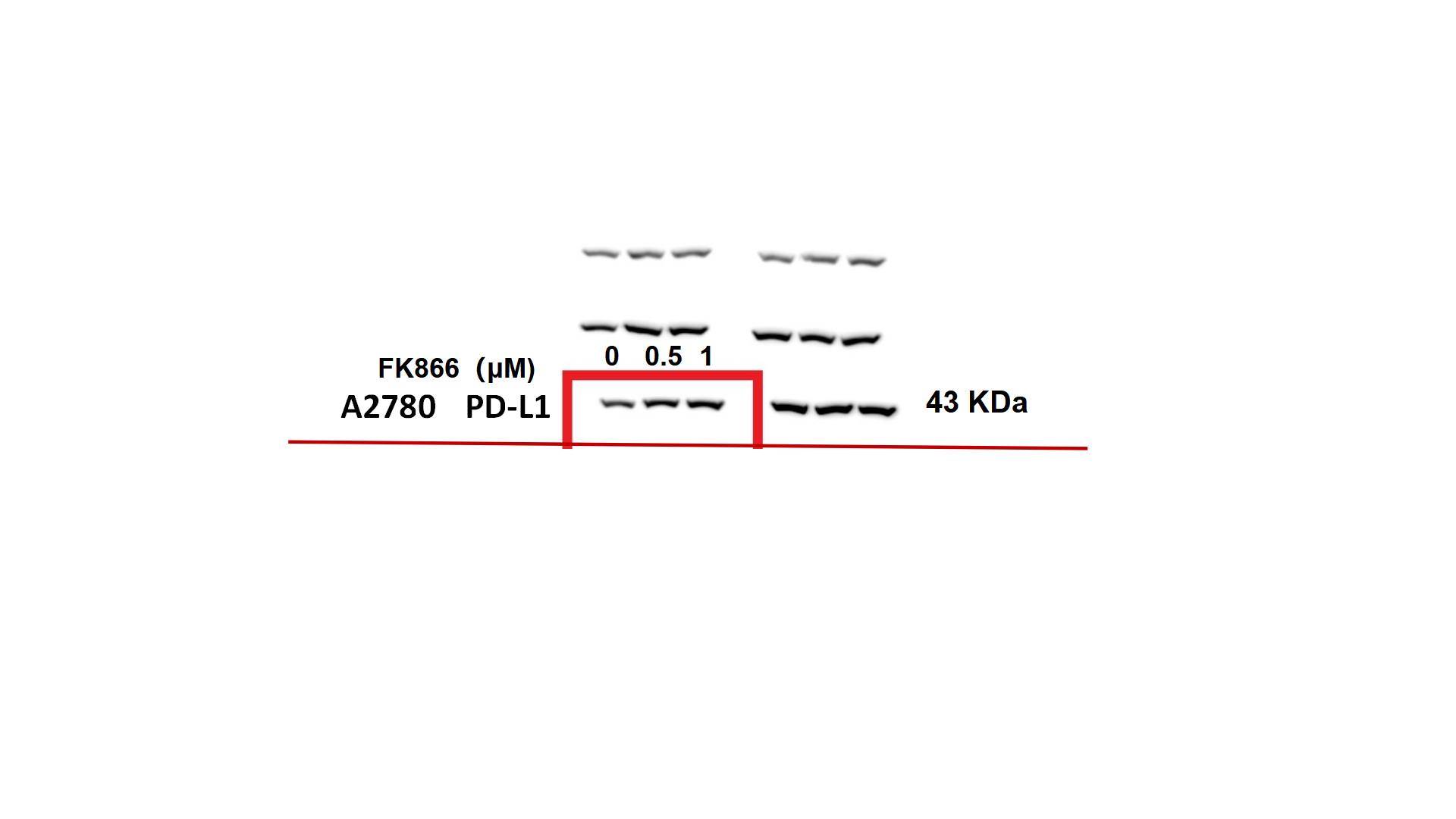

Supplement: Supplementary file 2 — Source Data Fig. 1 [file 44321_2024_51_MOESM2_ESM.zip › Fig-1/1D/Western Blot-A2780 PD-L1.tif]

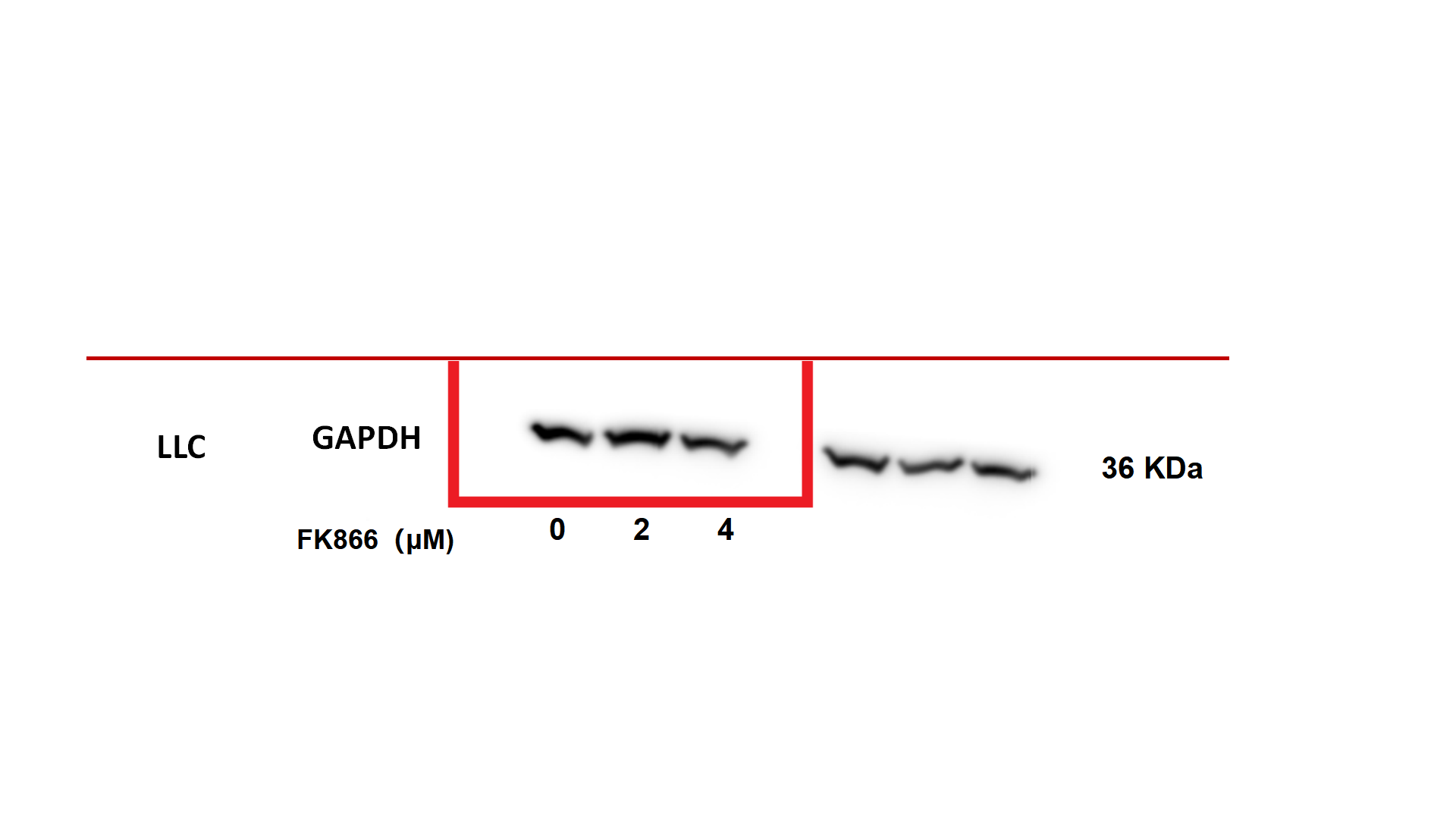

Supplement: Supplementary file 2 — Source Data Fig. 1 [file 44321_2024_51_MOESM2_ESM.zip › Fig-1/1D/Western Blot-LLC GAPDH.tif]

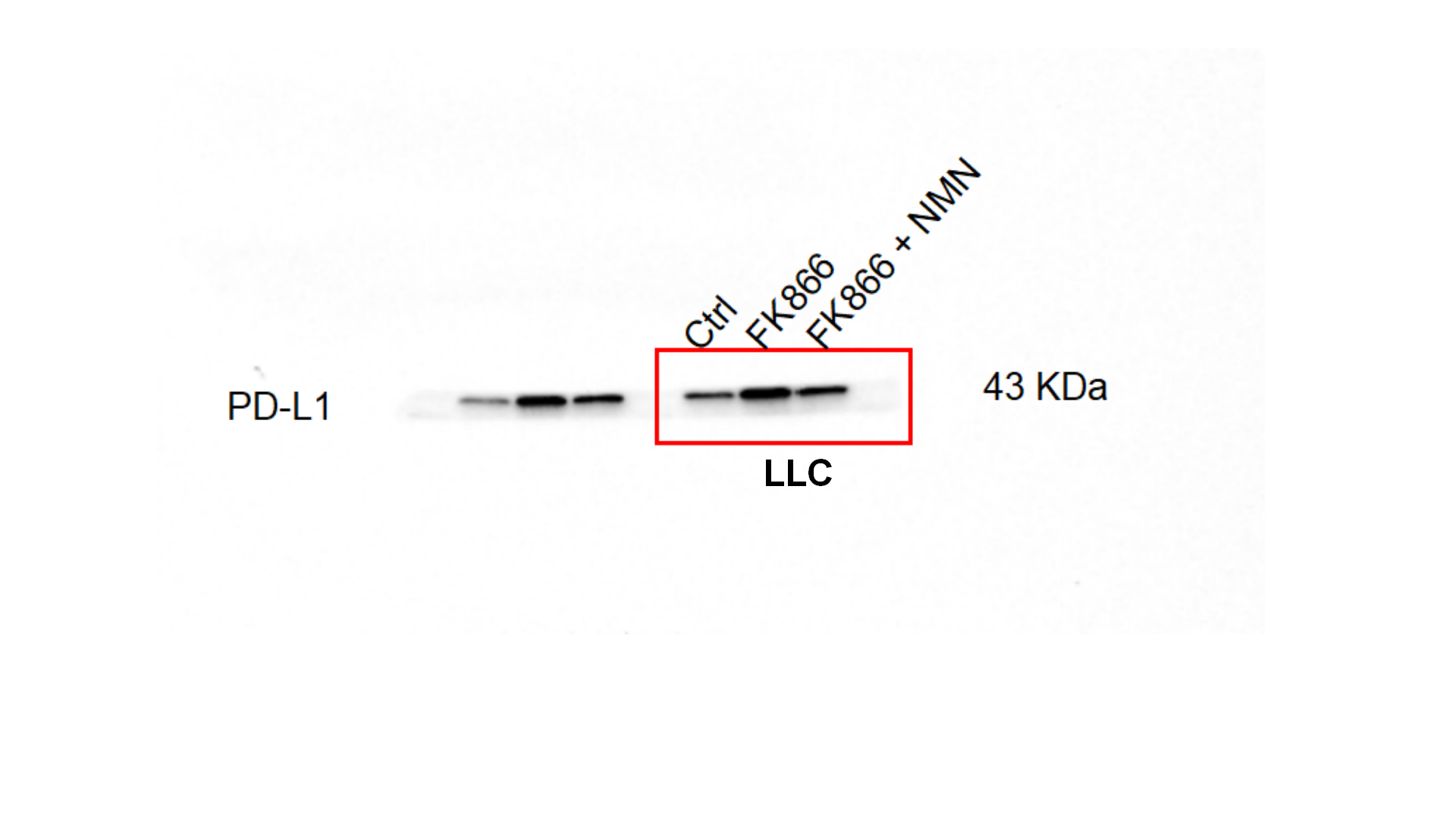

Supplement: Supplementary file 2 — Source Data Fig. 1 [file 44321_2024_51_MOESM2_ESM.zip › Fig-1/1I/PD-L1 LLC.tif]

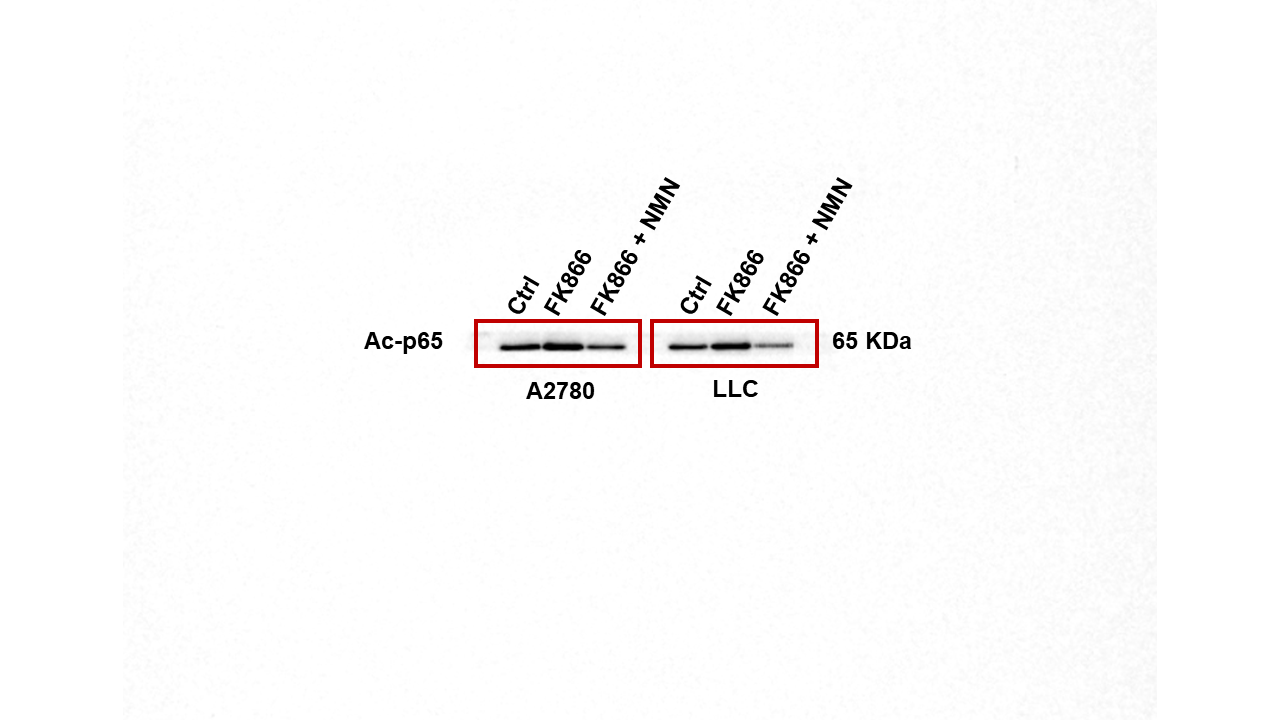

Supplement: Supplementary file 2 — Source Data Fig. 1 [file 44321_2024_51_MOESM2_ESM.zip › Fig-1/1I/Western Blot-Ac-p65 A2780 LLC.TIF]

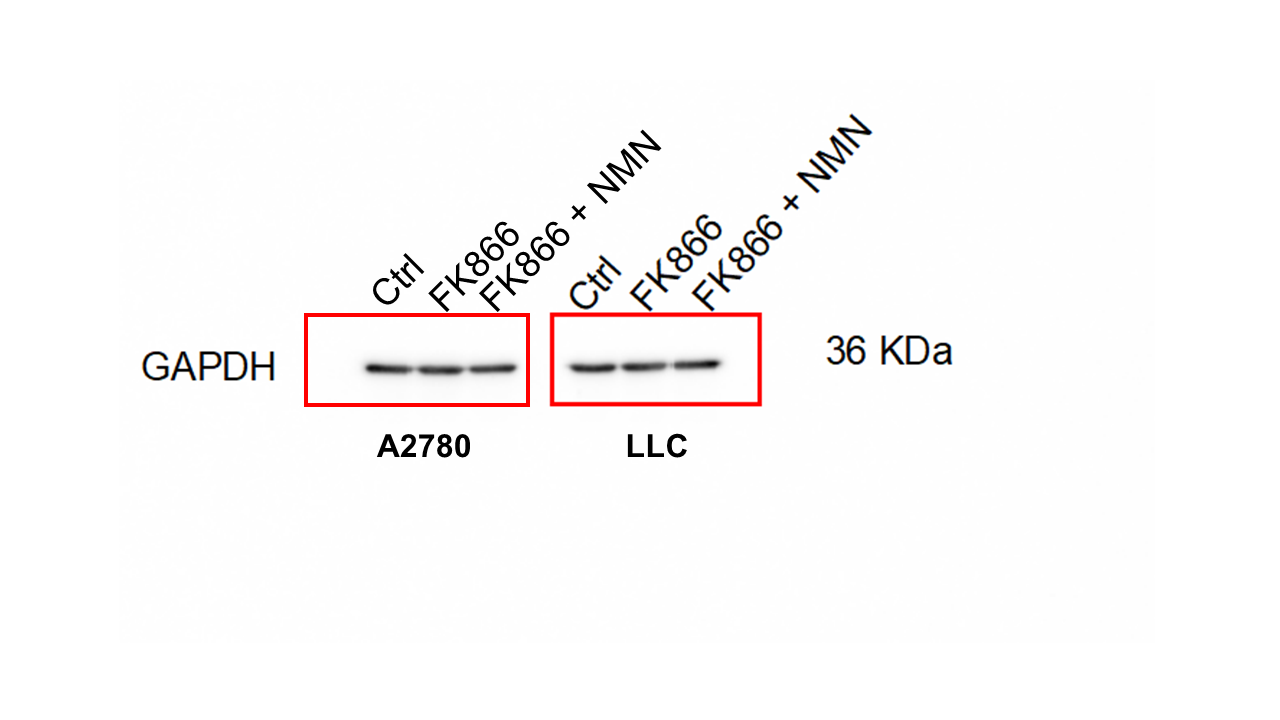

Supplement: Supplementary file 2 — Source Data Fig. 1 [file 44321_2024_51_MOESM2_ESM.zip › Fig-1/1I/Western Blot-GAPDH A2780 LLC.TIF]

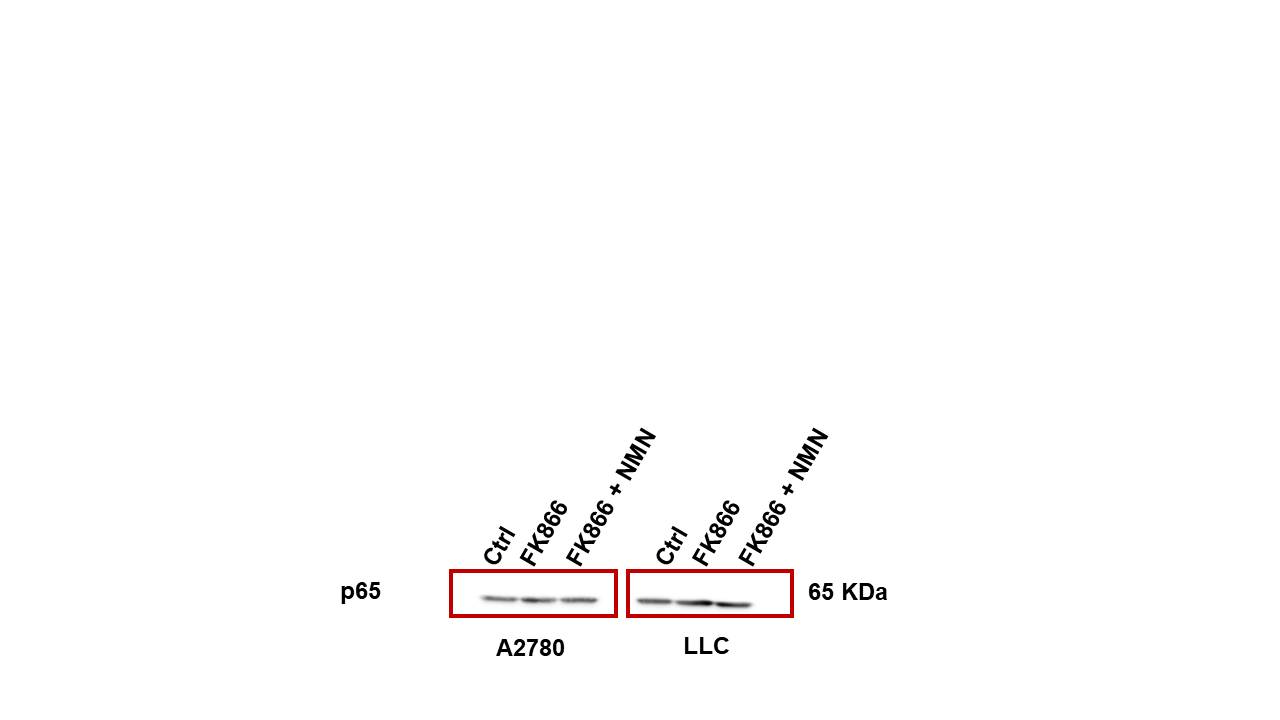

Supplement: Supplementary file 2 — Source Data Fig. 1 [file 44321_2024_51_MOESM2_ESM.zip › Fig-1/1I/Western Blot-p65 A2780 LLC.TIF]

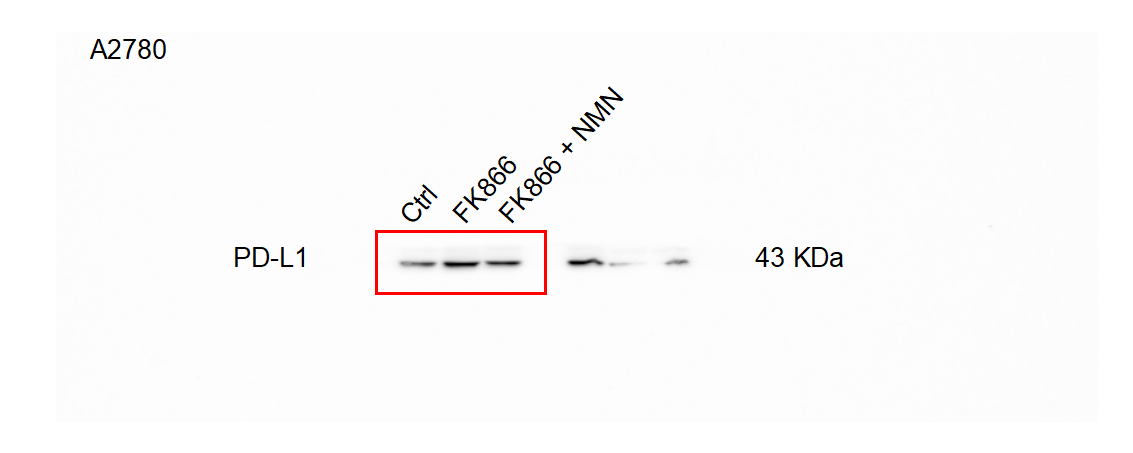

Supplement: Supplementary file 2 — Source Data Fig. 1 [file 44321_2024_51_MOESM2_ESM.zip › Fig-1/1I/Western Blot-PD-L1 A2780.tif]

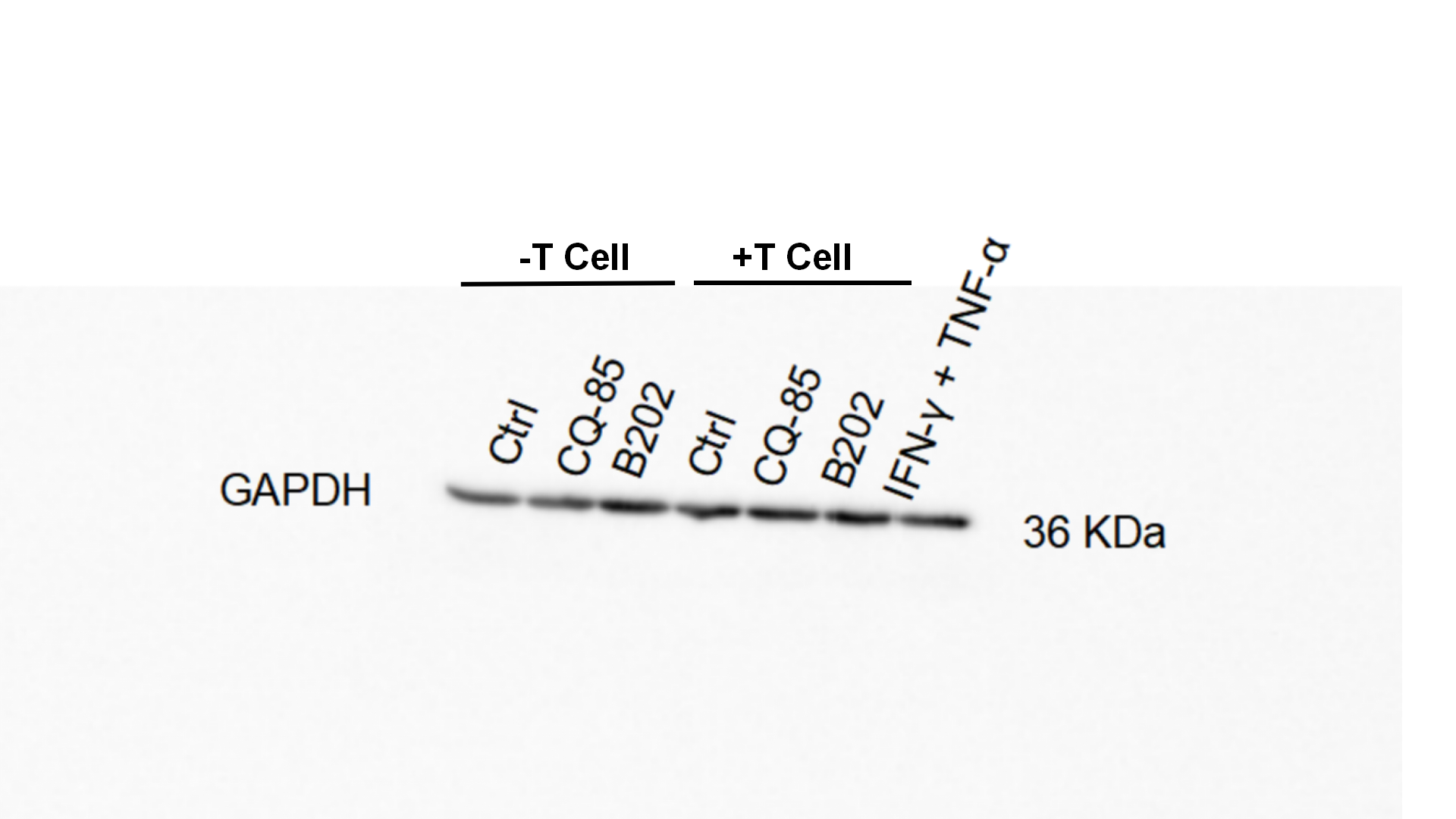

Supplement: Supplementary file 3 — Source Data Fig. 2 [file 44321_2024_51_MOESM3_ESM.zip › Fig-2/Fig-2-B/Western Blot-GAPDH.tif]

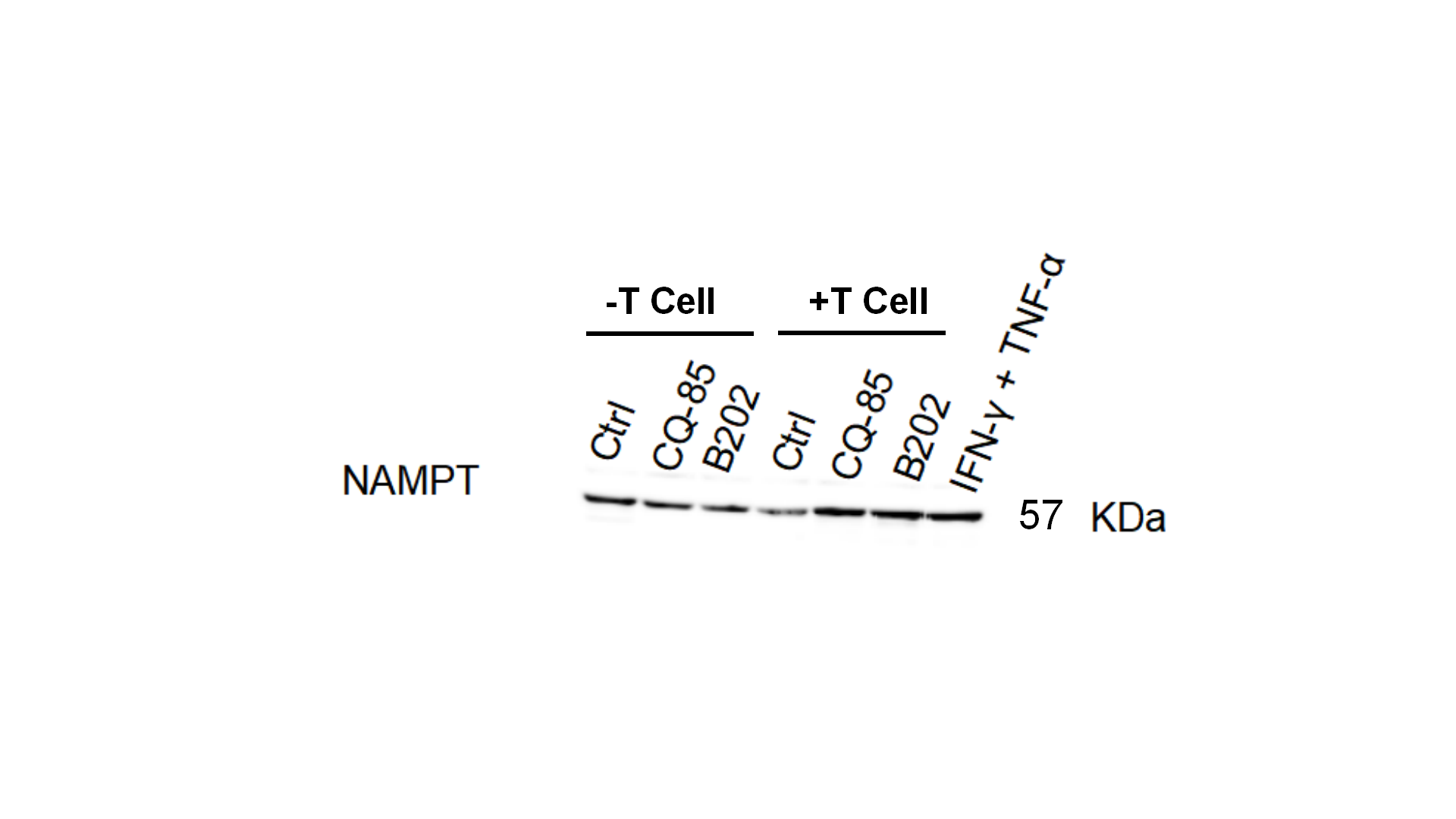

Supplement: Supplementary file 3 — Source Data Fig. 2 [file 44321_2024_51_MOESM3_ESM.zip › Fig-2/Fig-2-B/Western Blot-NAMPT.tif]

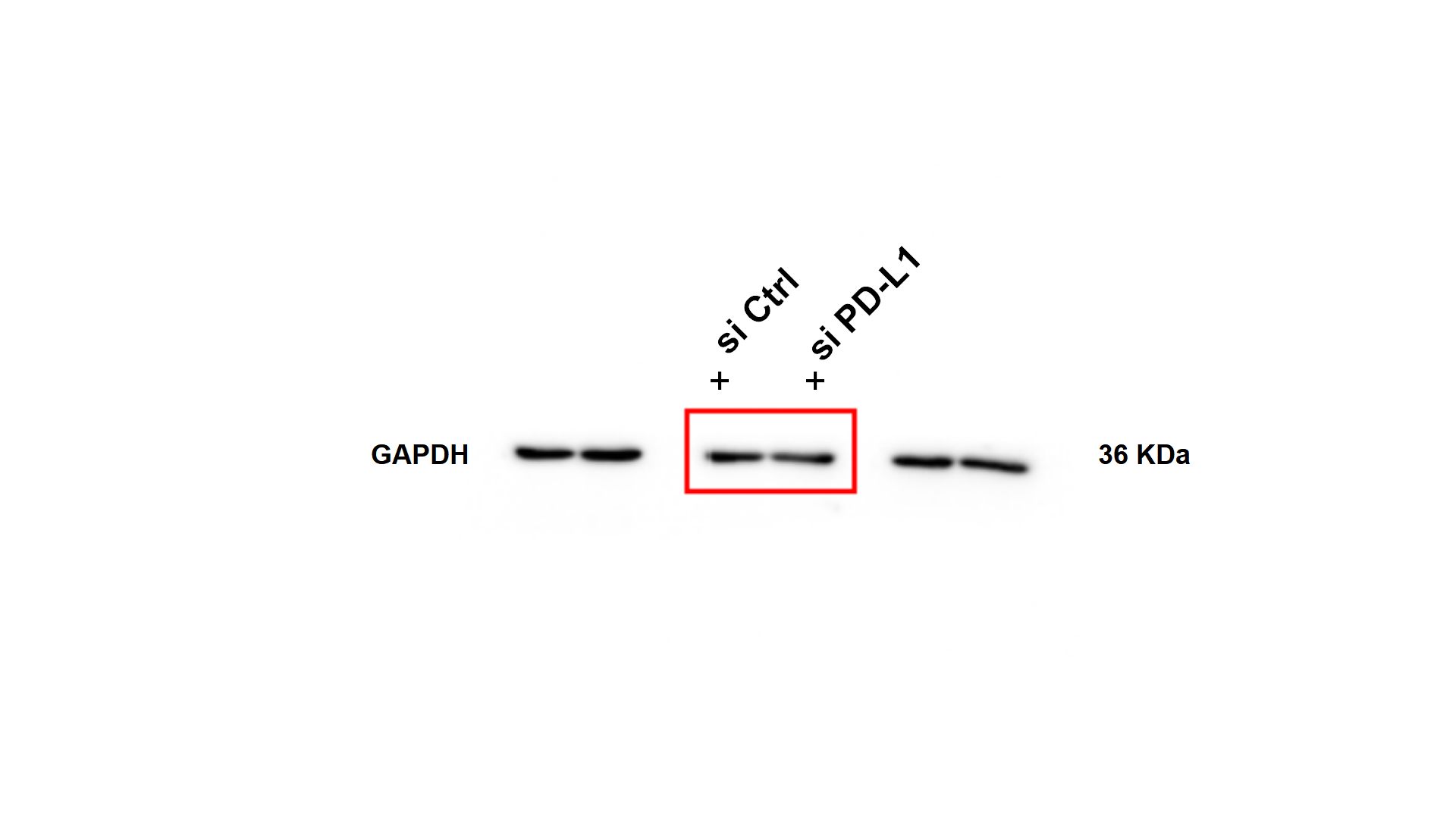

Supplement: Supplementary file 3 — Source Data Fig. 2 [file 44321_2024_51_MOESM3_ESM.zip › Fig-2/Fig-2-C/Western Blot-GAPDH.tif]

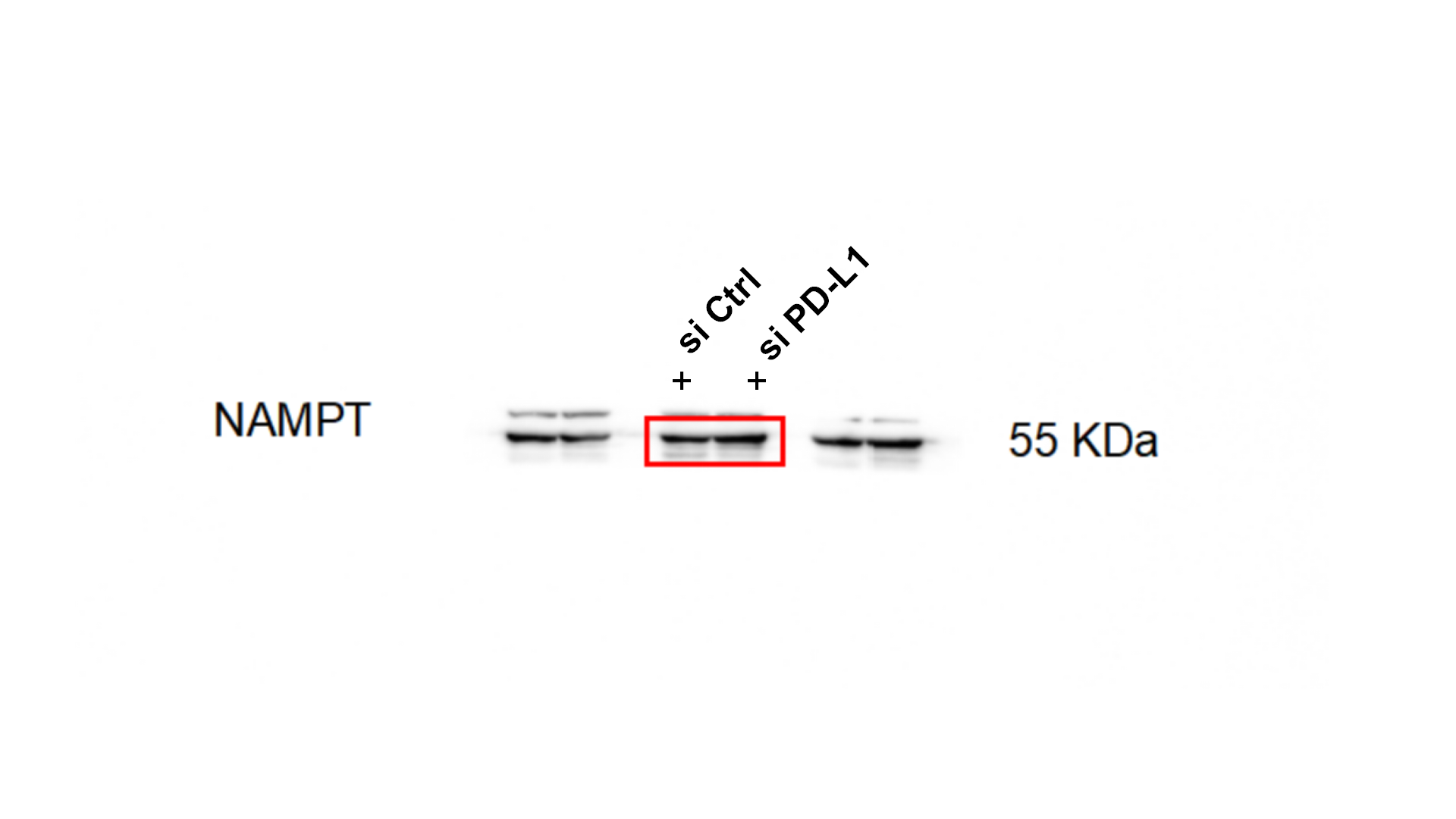

Supplement: Supplementary file 3 — Source Data Fig. 2 [file 44321_2024_51_MOESM3_ESM.zip › Fig-2/Fig-2-C/Western Blot-NAMPT.tif]

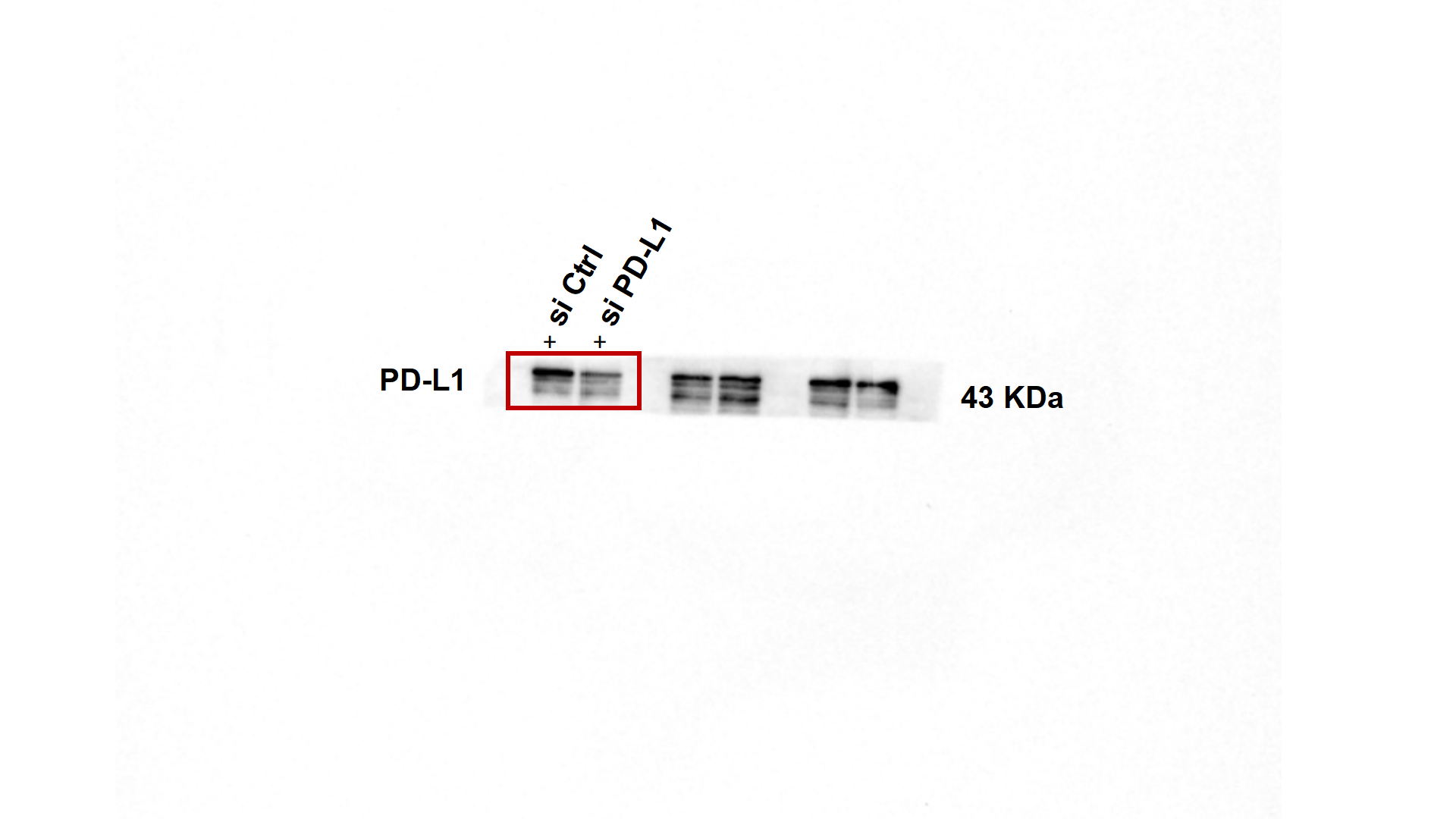

Supplement: Supplementary file 3 — Source Data Fig. 2 [file 44321_2024_51_MOESM3_ESM.zip › Fig-2/Fig-2-C/Western Blot-PD-L1.tif]

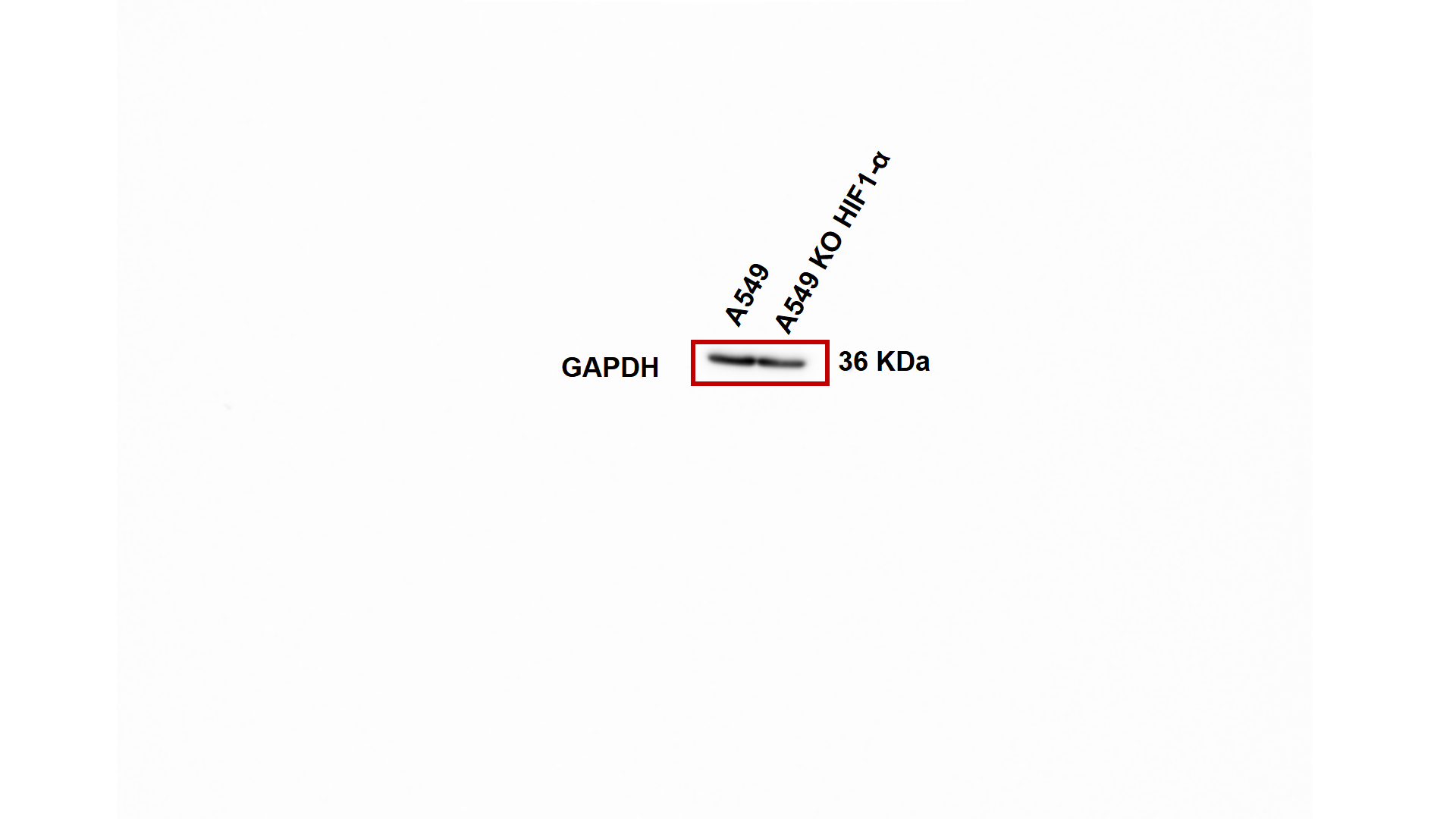

Supplement: Supplementary file 3 — Source Data Fig. 2 [file 44321_2024_51_MOESM3_ESM.zip › Fig-2/Fig-2-I/Western Blot-GAPDH+HIF-1α/Western Blot-GAPDH.tif]

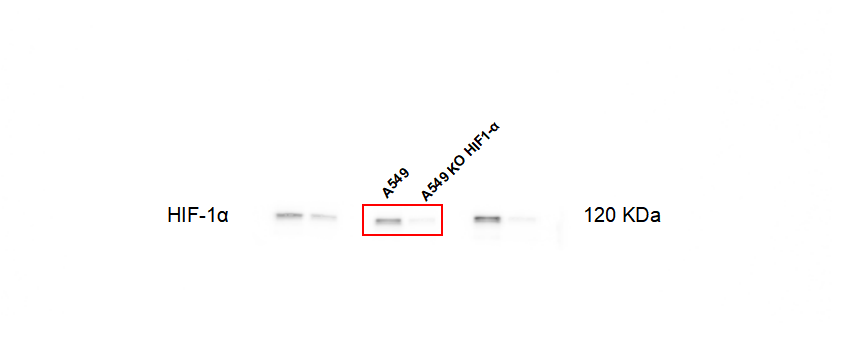

Supplement: Supplementary file 3 — Source Data Fig. 2 [file 44321_2024_51_MOESM3_ESM.zip › Fig-2/Fig-2-I/Western Blot-GAPDH+HIF-1α/Western Blot-HIF-1α.tif]

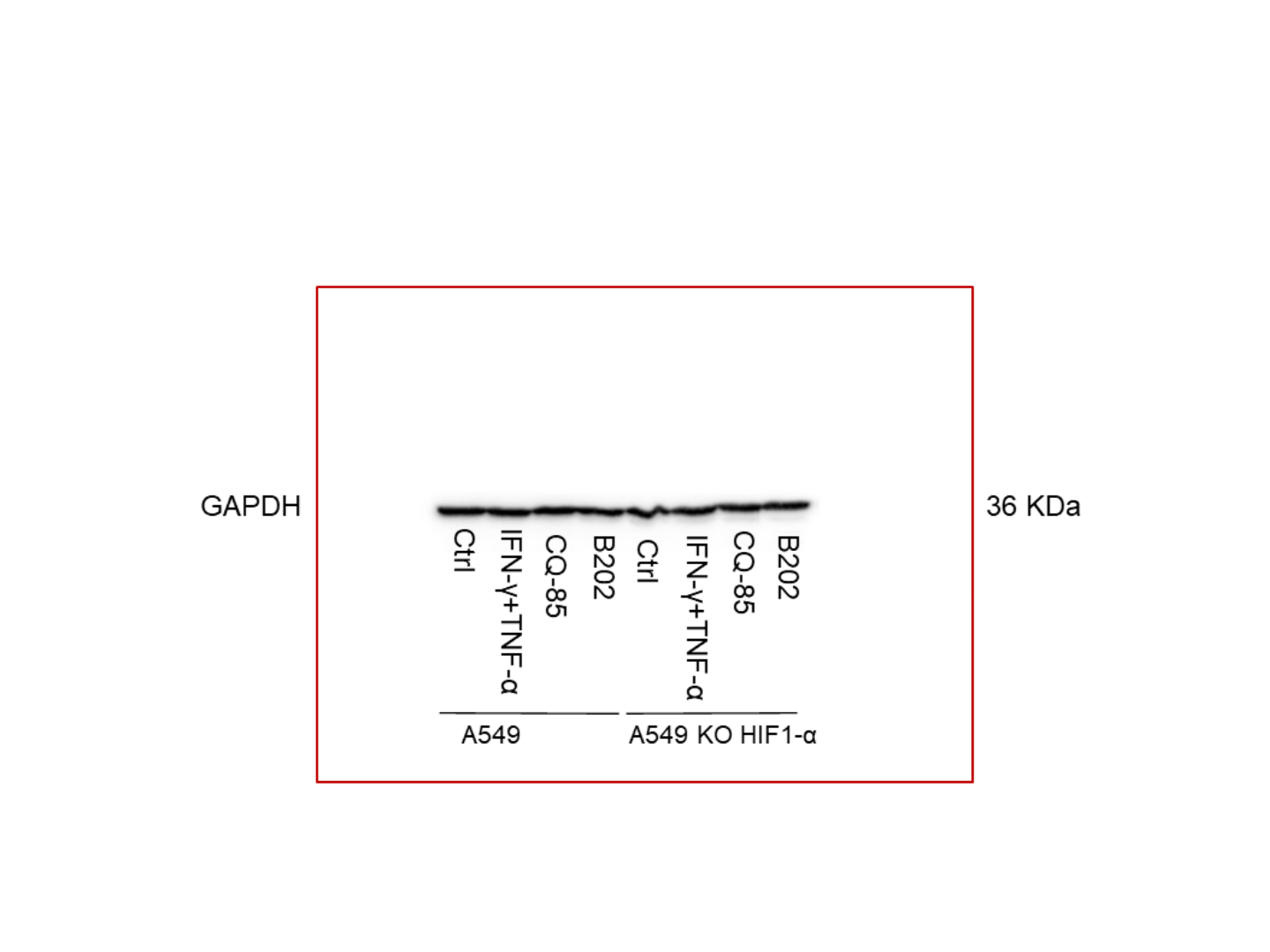

Supplement: Supplementary file 3 — Source Data Fig. 2 [file 44321_2024_51_MOESM3_ESM.zip › Fig-2/Fig-2-I/Western Blot-NAMPT GAPDH/Western Blot-GAPDH.tif]

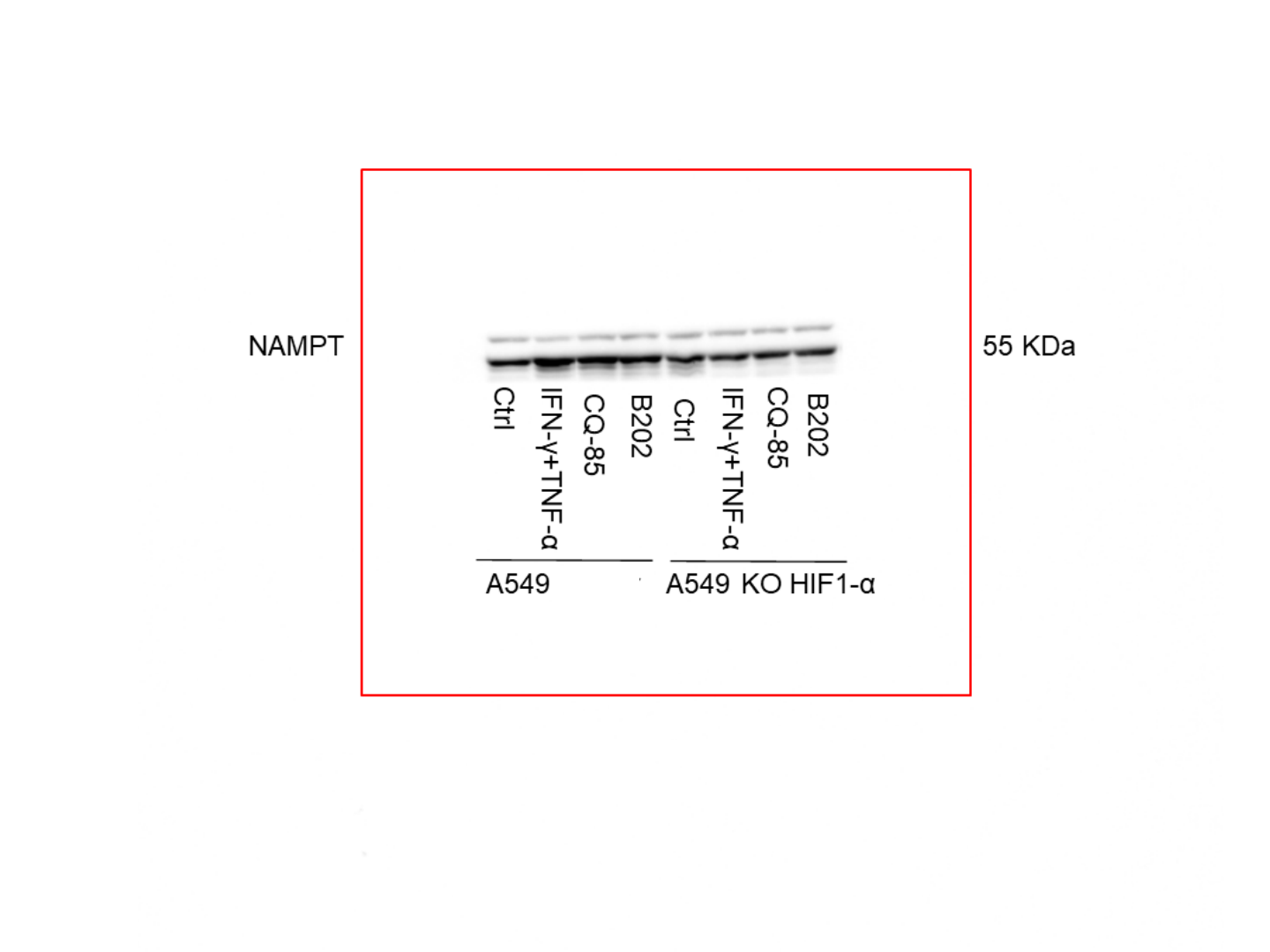

Supplement: Supplementary file 3 — Source Data Fig. 2 [file 44321_2024_51_MOESM3_ESM.zip › Fig-2/Fig-2-I/Western Blot-NAMPT GAPDH/Western Blot-NAMPT.tif]

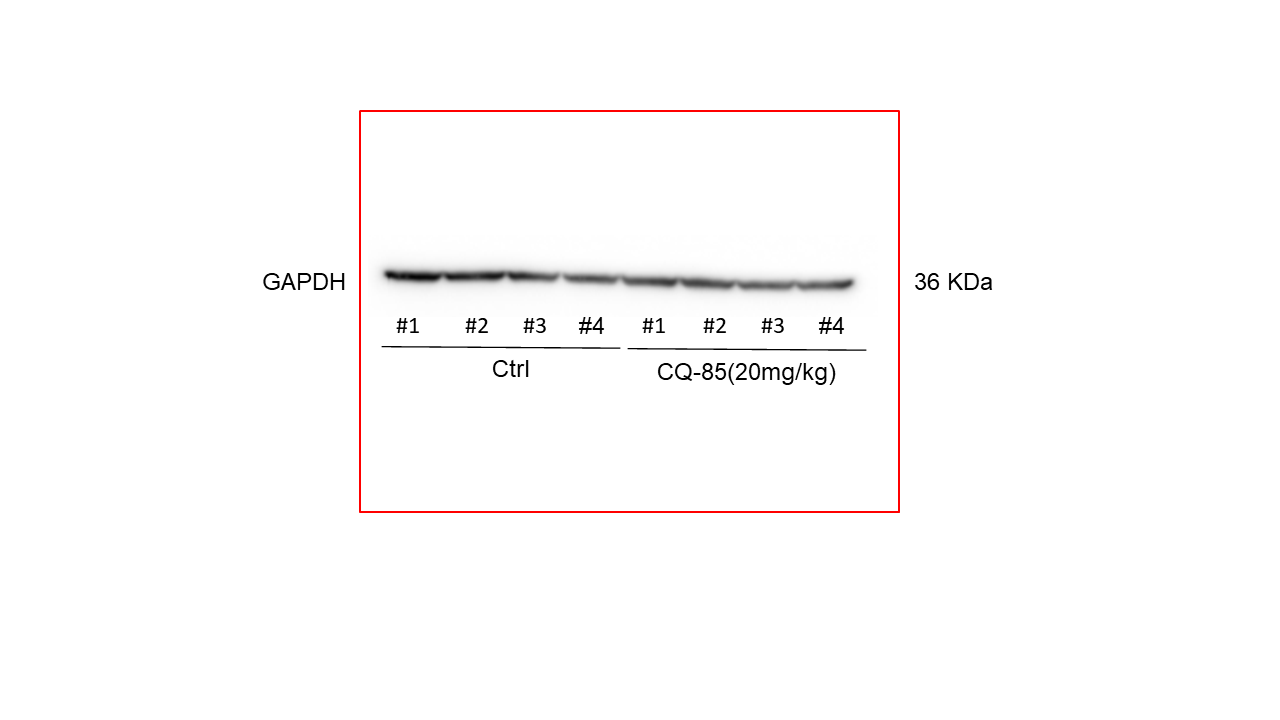

Supplement: Supplementary file 3 — Source Data Fig. 2 [file 44321_2024_51_MOESM3_ESM.zip › Fig-2/Fig-2-A/Western Blot-GAPDH.TIF]

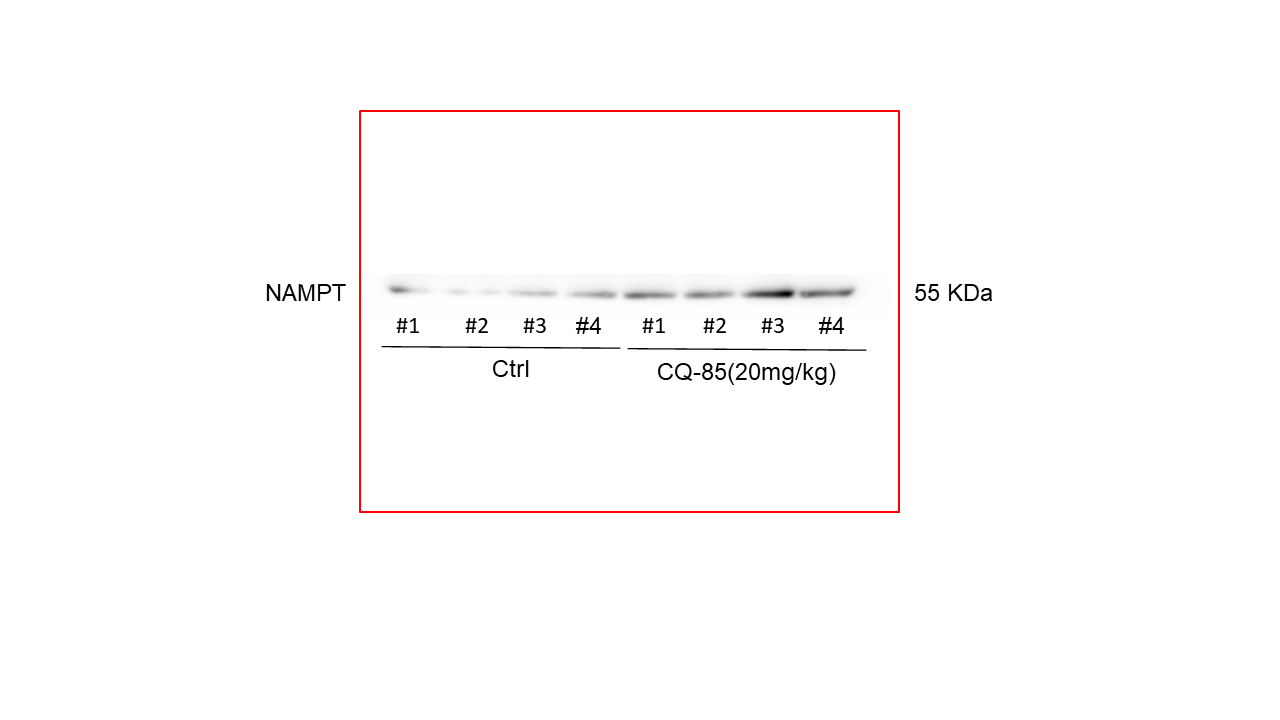

Supplement: Supplementary file 3 — Source Data Fig. 2 [file 44321_2024_51_MOESM3_ESM.zip › Fig-2/Fig-2-A/Western Blot-NAMPT.TIF]

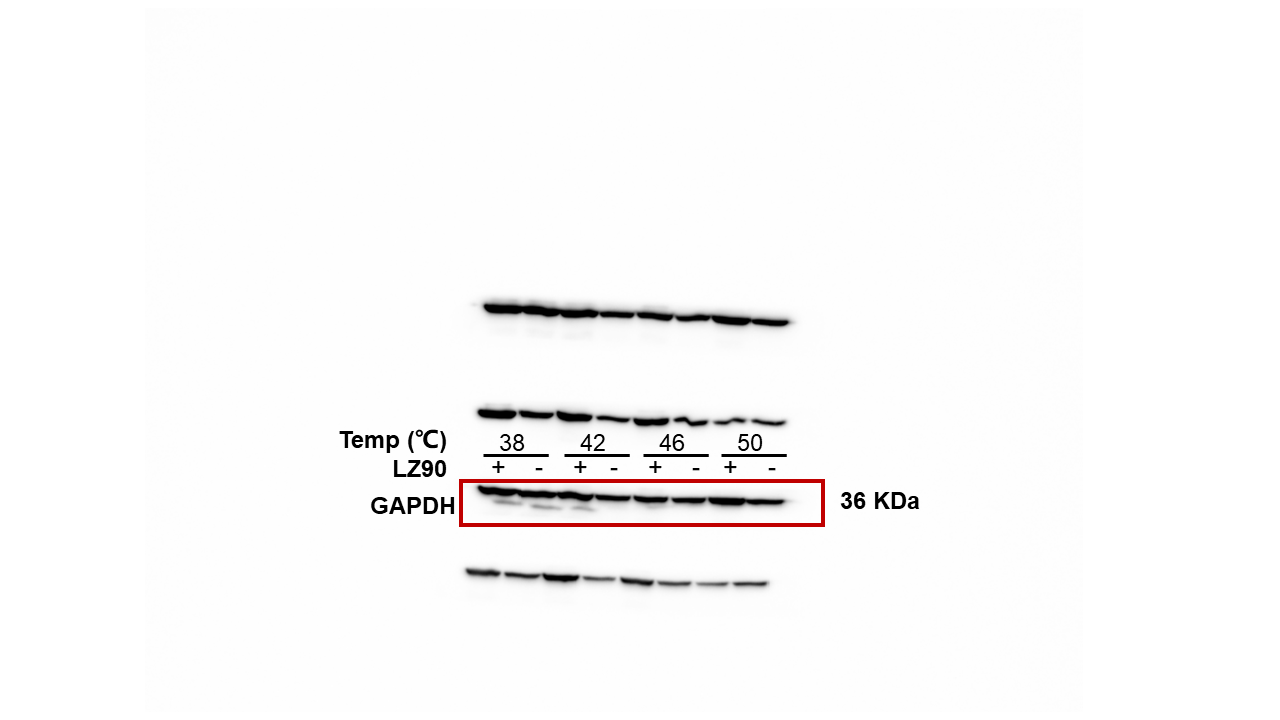

Supplement: Supplementary file 4 — Source Data Fig. 3 [file 44321_2024_51_MOESM4_ESM.zip › Fig-3/3C/Western Blot-GAPDH 38-50°C.tif]

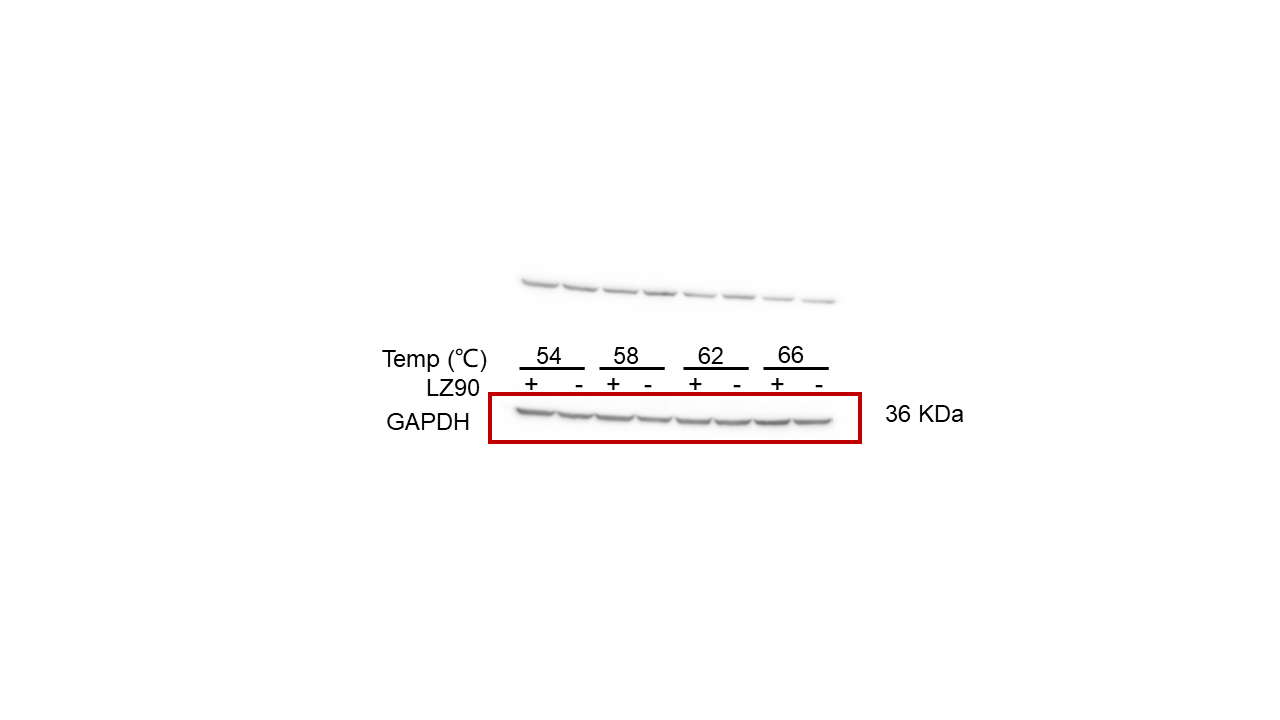

Supplement: Supplementary file 4 — Source Data Fig. 3 [file 44321_2024_51_MOESM4_ESM.zip › Fig-3/3C/Western Blot-GAPDH 54-66°C.tif]

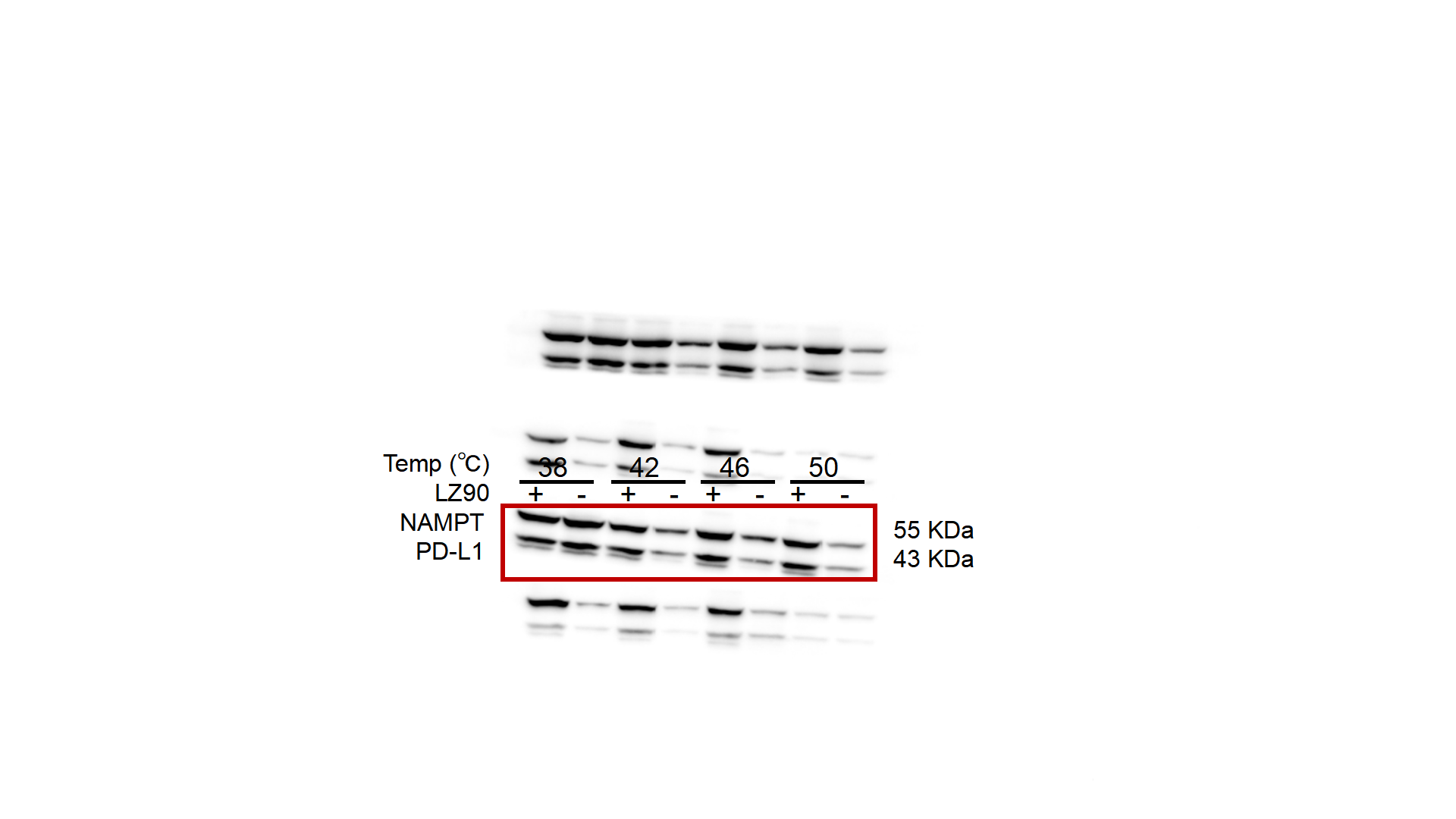

Supplement: Supplementary file 4 — Source Data Fig. 3 [file 44321_2024_51_MOESM4_ESM.zip › Fig-3/3C/Western Blot-NAMPT PD-L1 38-50°C.tif]

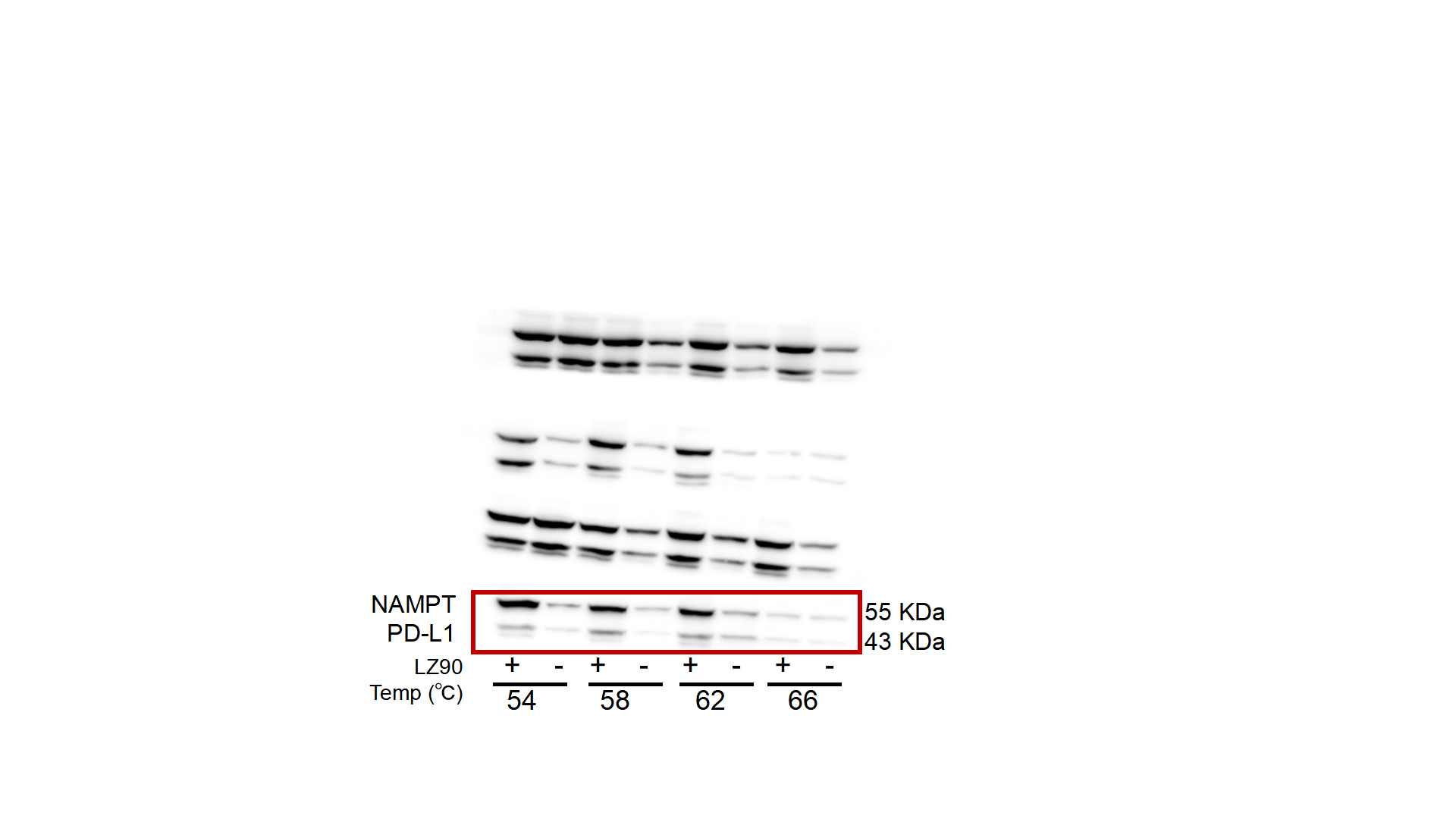

Supplement: Supplementary file 4 — Source Data Fig. 3 [file 44321_2024_51_MOESM4_ESM.zip › Fig-3/3C/Western Blot-NAMPT PD-L1 54-66°C.tif]

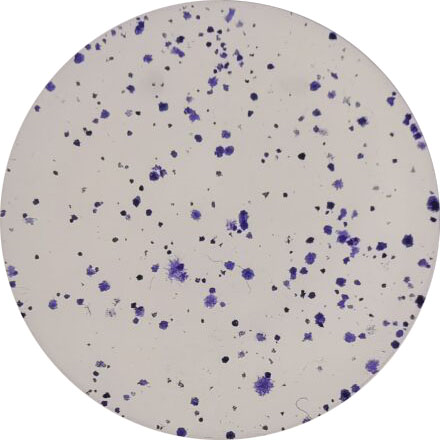

Supplement: Supplementary file 5 — Source Data Fig. 4 [file 44321_2024_51_MOESM5_ESM.zip › Fig-4/Fig4/4A/A2780/A2780 LZ90 0.5nM.tif]

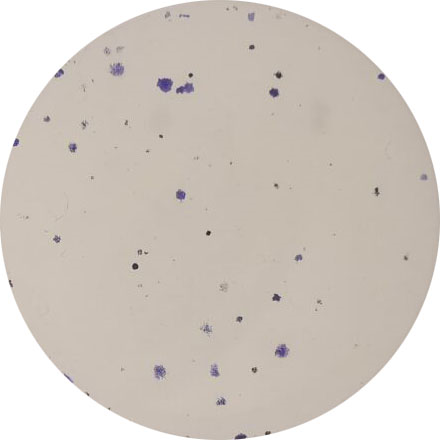

Supplement: Supplementary file 5 — Source Data Fig. 4 [file 44321_2024_51_MOESM5_ESM.zip › Fig-4/Fig4/4A/A2780/A2780 LZ90 1nM-1.tif]

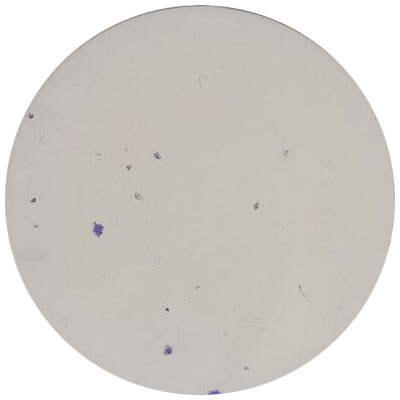

Supplement: Supplementary file 5 — Source Data Fig. 4 [file 44321_2024_51_MOESM5_ESM.zip › Fig-4/Fig4/4A/A2780/A2780 LZ90 2nM.tif]

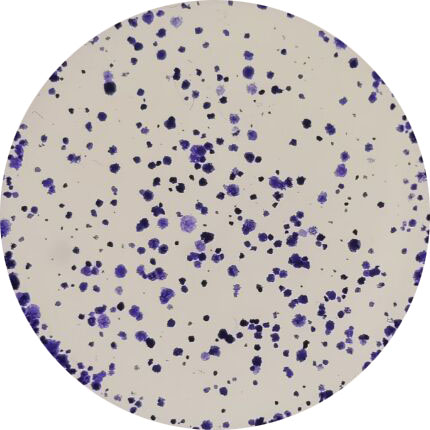

Supplement: Supplementary file 5 — Source Data Fig. 4 [file 44321_2024_51_MOESM5_ESM.zip › Fig-4/Fig4/4A/A2780/A2780 CON.tif]

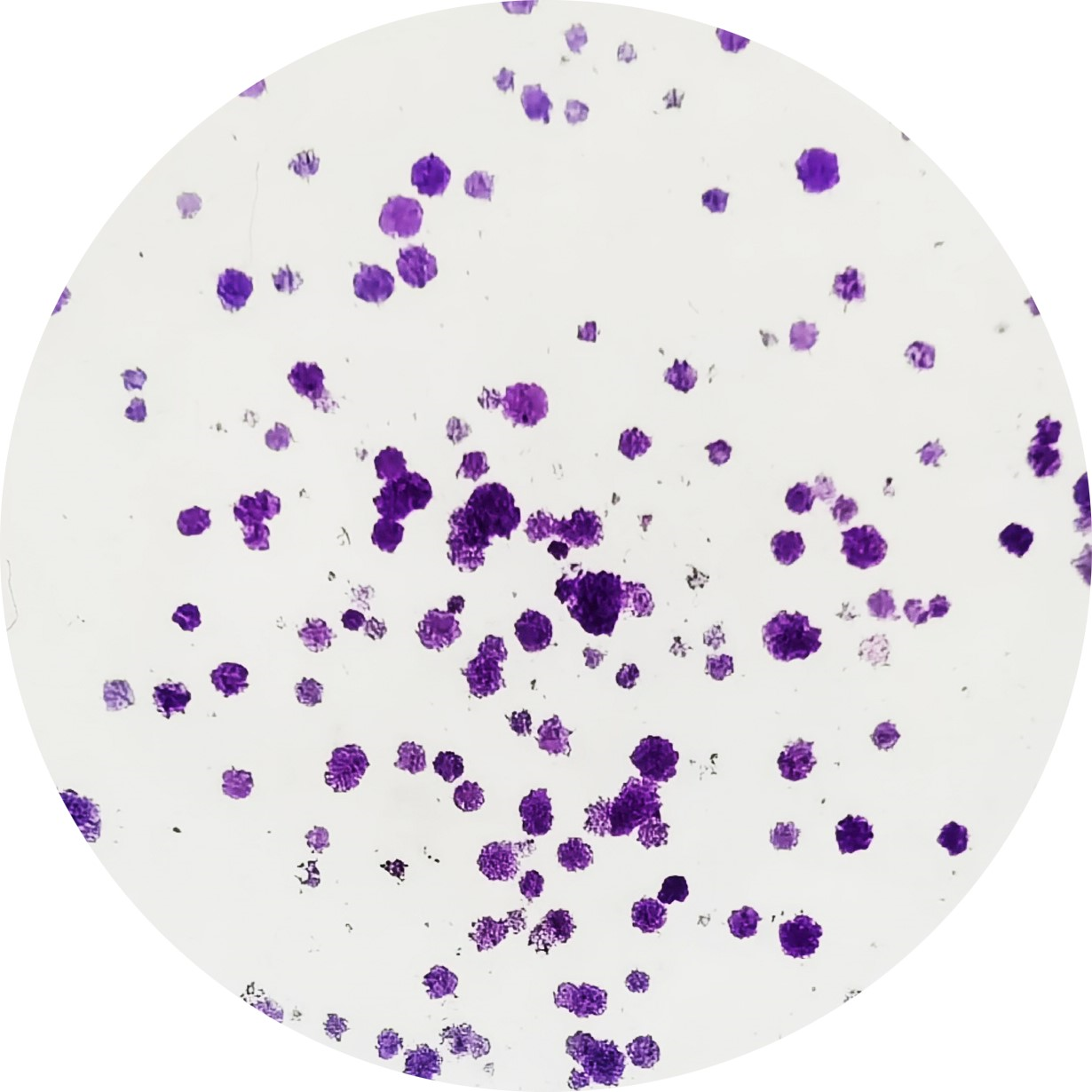

Supplement: Supplementary file 5 — Source Data Fig. 4 [file 44321_2024_51_MOESM5_ESM.zip › Fig-4/Fig4/4A/HCT-116/HCT-116 Ctrl.tif]

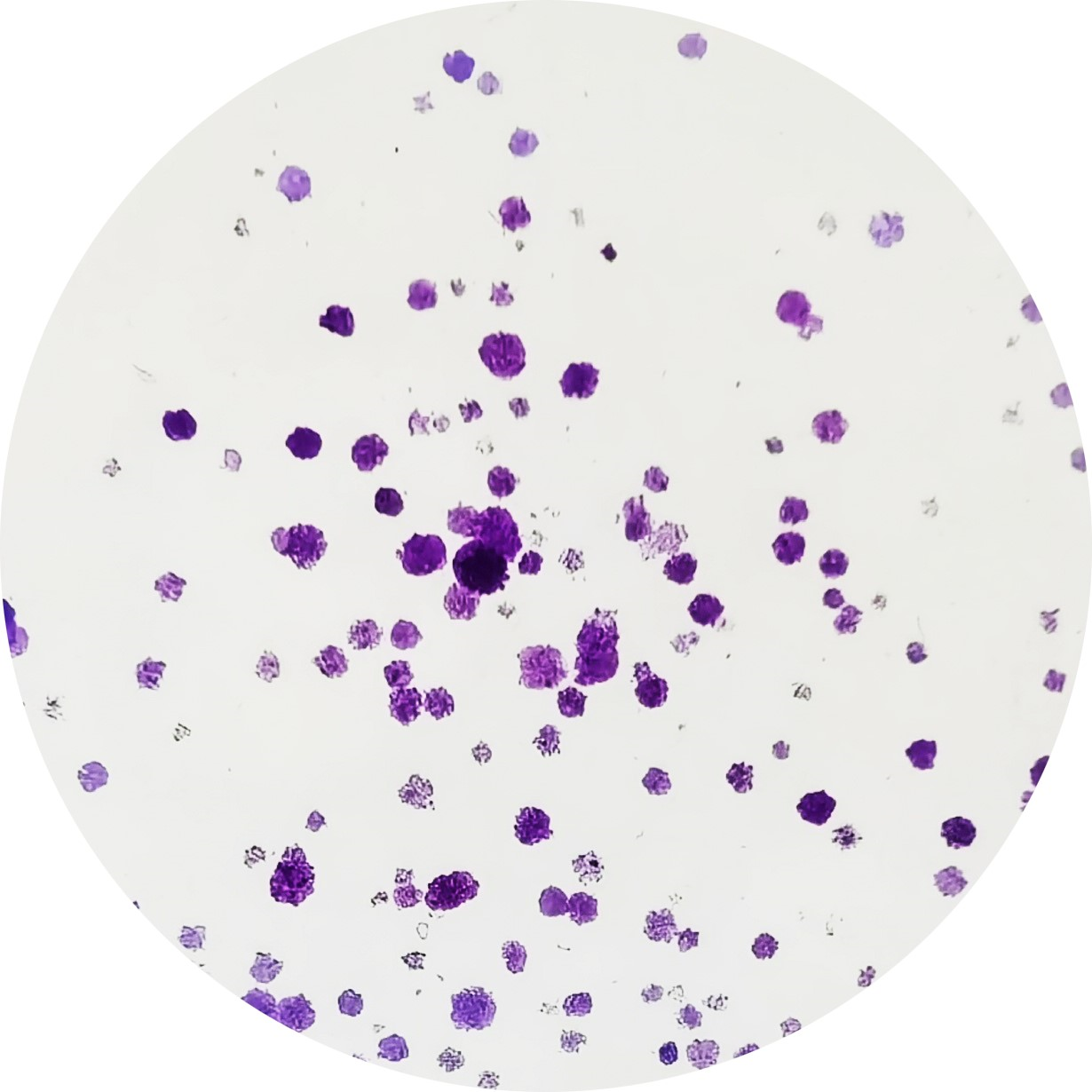

Supplement: Supplementary file 5 — Source Data Fig. 4 [file 44321_2024_51_MOESM5_ESM.zip › Fig-4/Fig4/4A/HCT-116/HCT-116 LZ90 0.5 nM.tif]

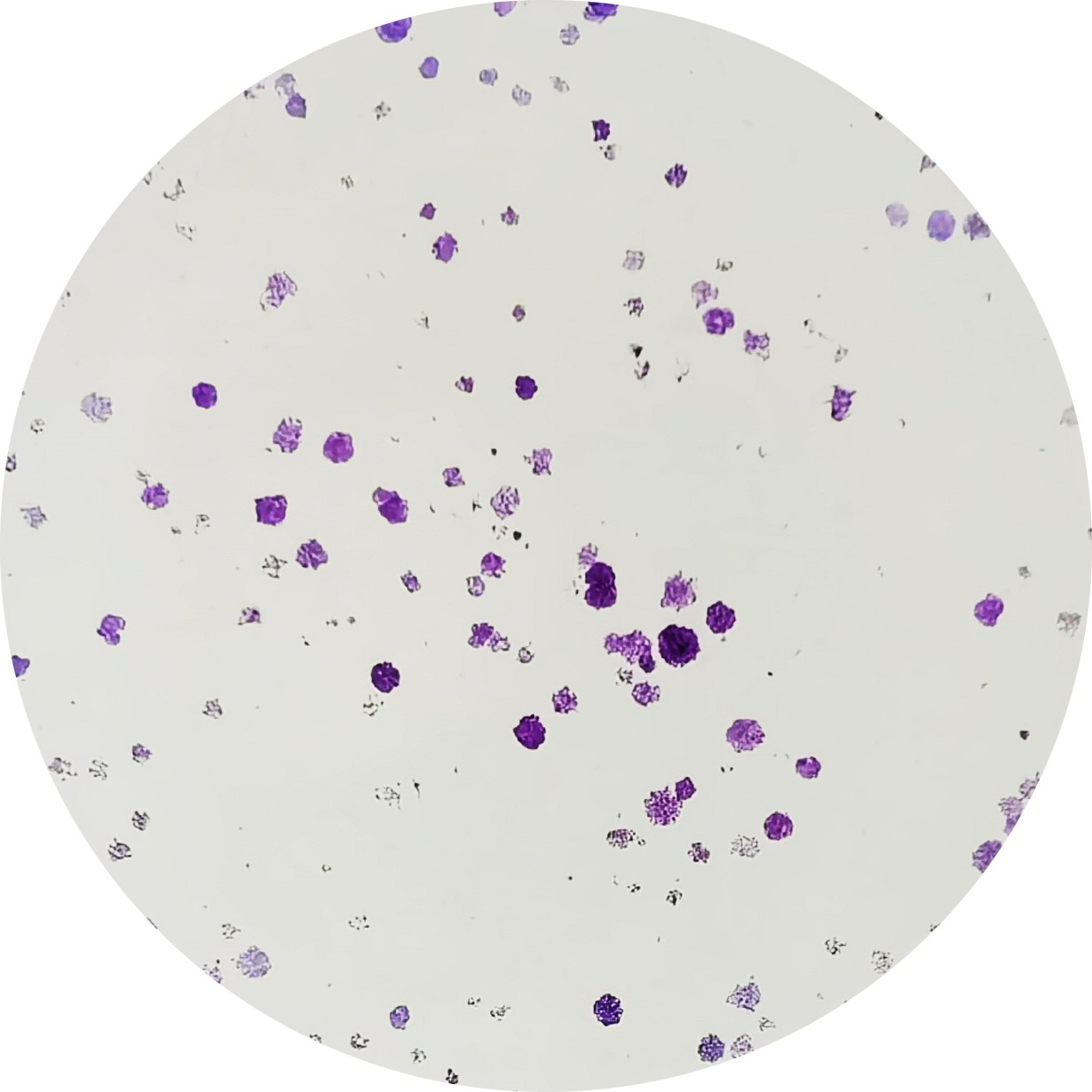

Supplement: Supplementary file 5 — Source Data Fig. 4 [file 44321_2024_51_MOESM5_ESM.zip › Fig-4/Fig4/4A/HCT-116/HCT-116 LZ90 1 nM.tif]

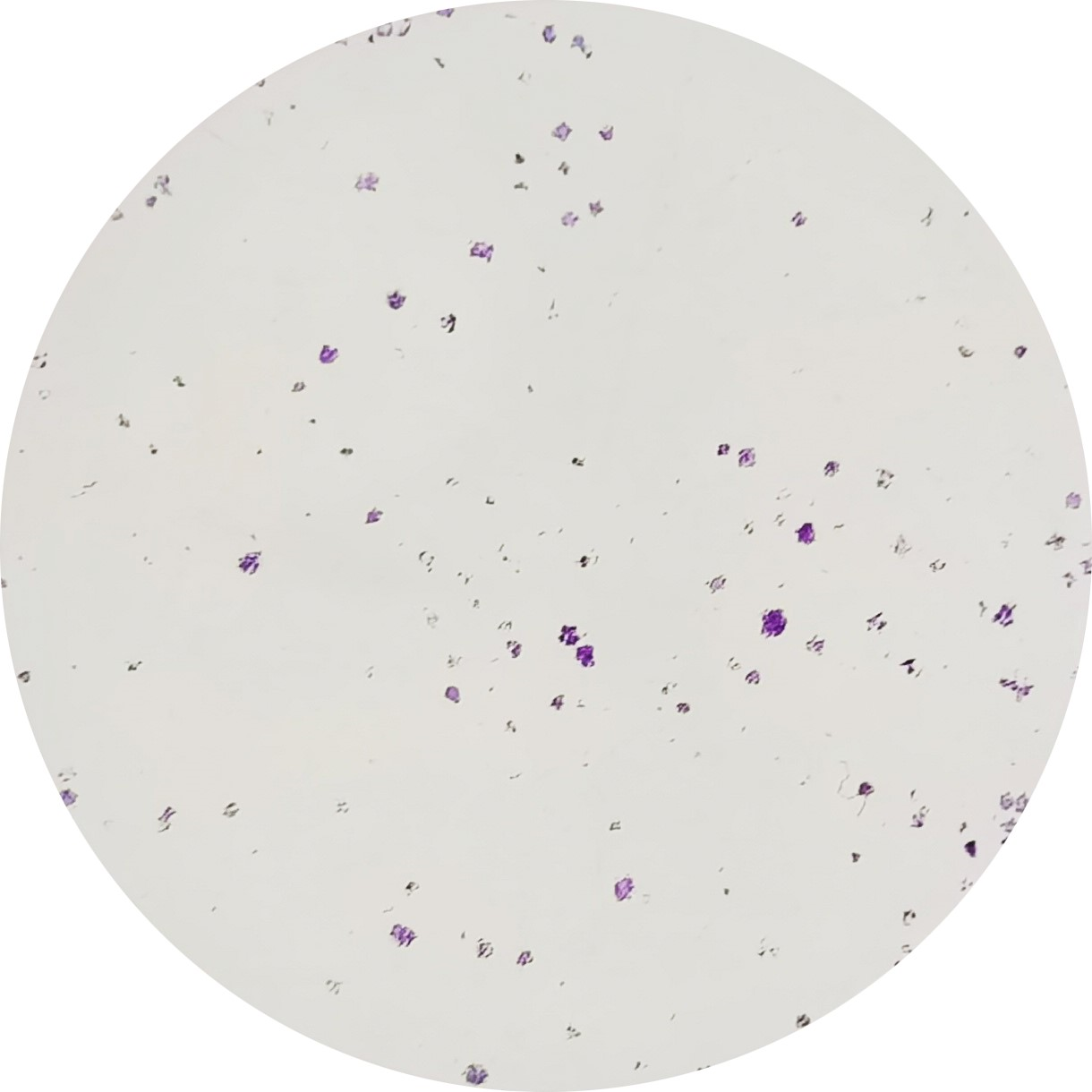

Supplement: Supplementary file 5 — Source Data Fig. 4 [file 44321_2024_51_MOESM5_ESM.zip › Fig-4/Fig4/4A/HCT-116/HCT-116 LZ90 2 nM.tif]

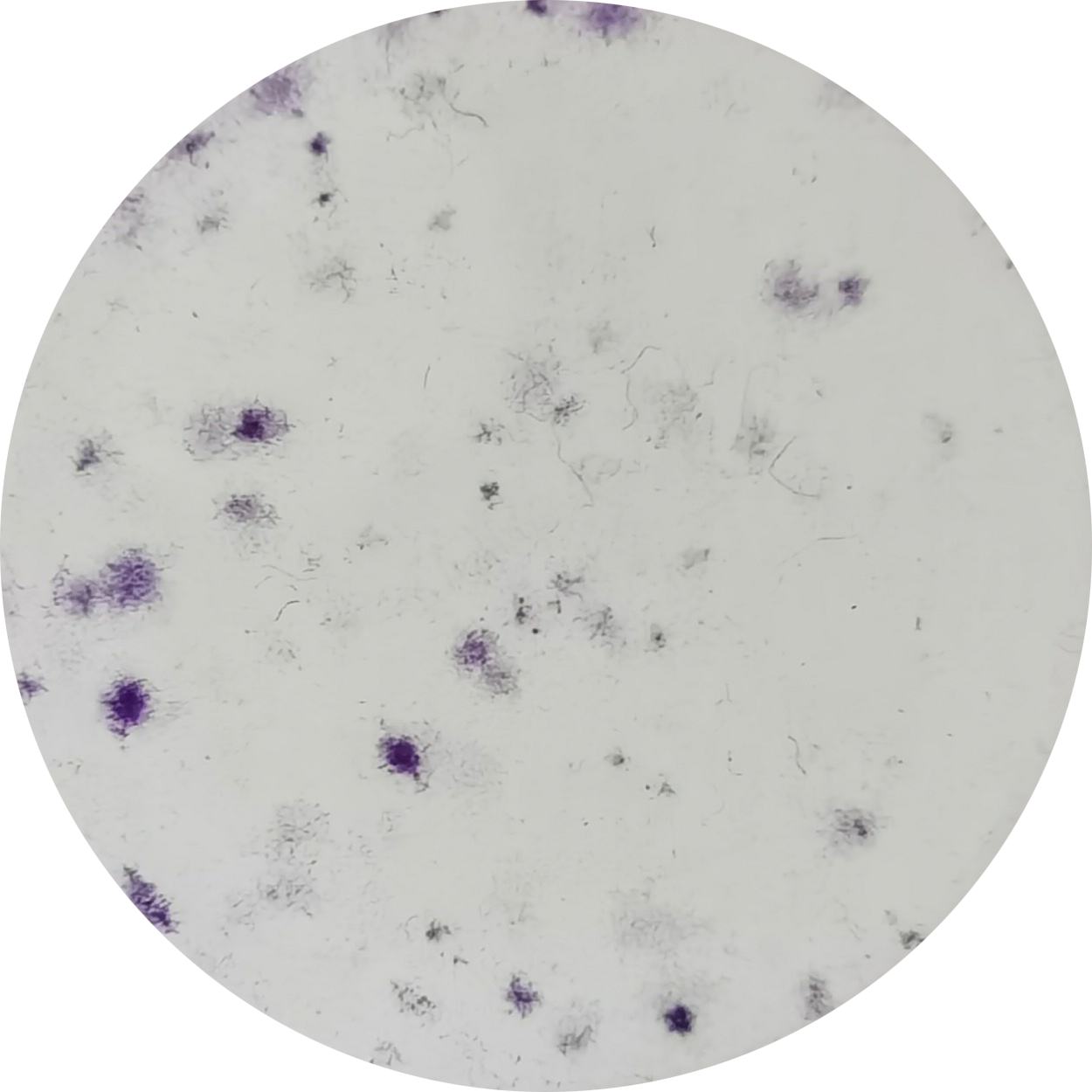

Supplement: Supplementary file 5 — Source Data Fig. 4 [file 44321_2024_51_MOESM5_ESM.zip › Fig-4/Fig4/4A/LLC/LLC Ctrl.tif]

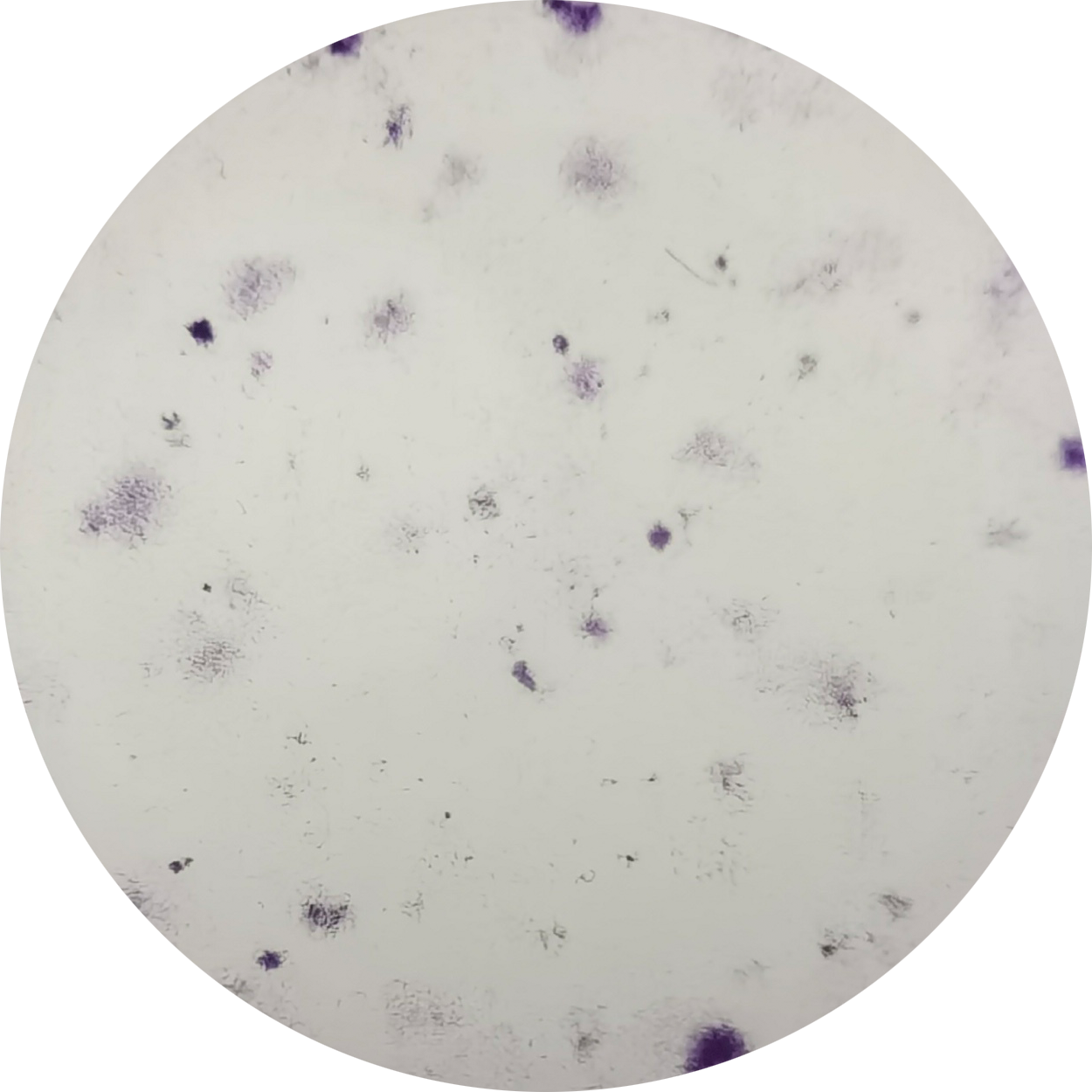

Supplement: Supplementary file 5 — Source Data Fig. 4 [file 44321_2024_51_MOESM5_ESM.zip › Fig-4/Fig4/4A/LLC/LLC 40 nM.tif]

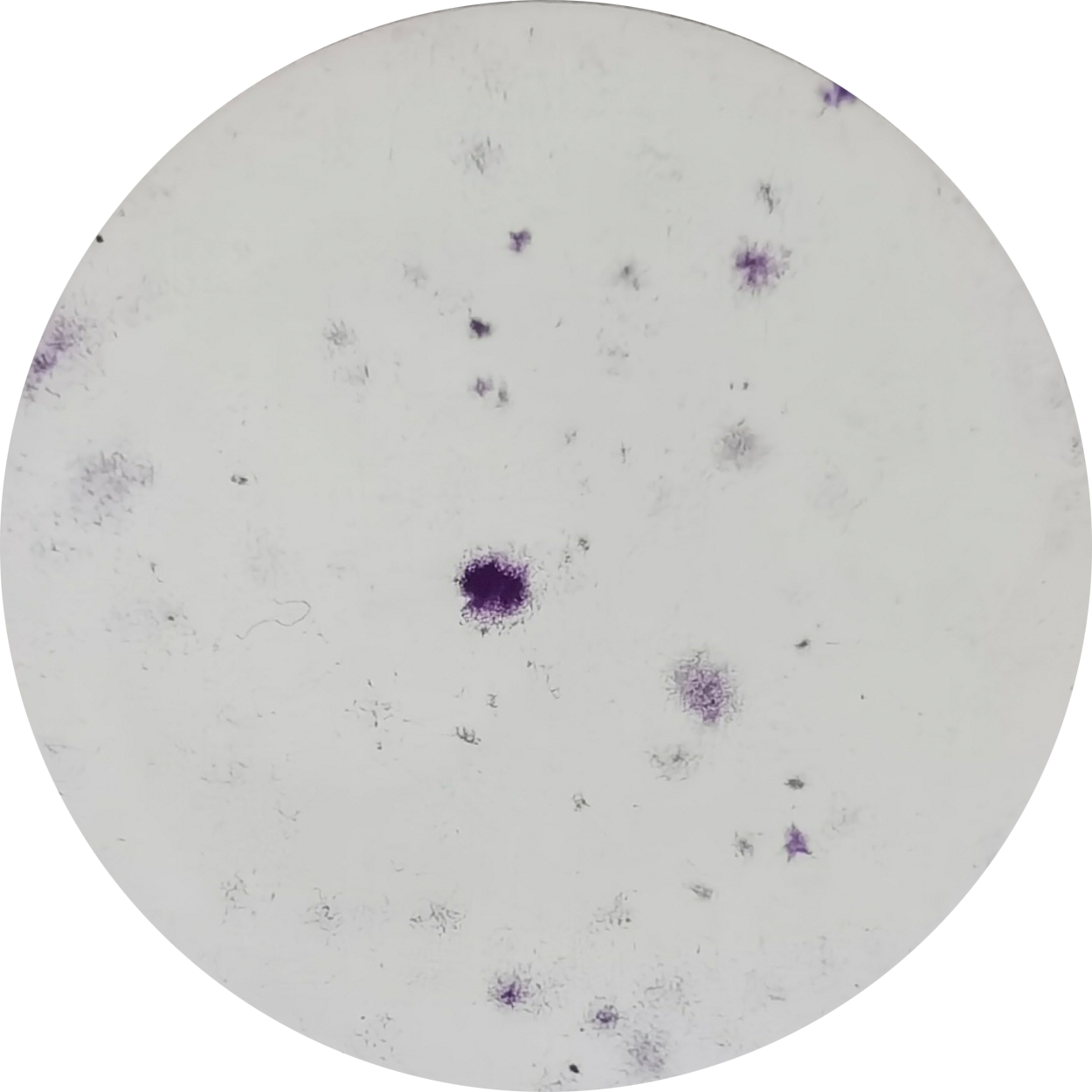

Supplement: Supplementary file 5 — Source Data Fig. 4 [file 44321_2024_51_MOESM5_ESM.zip › Fig-4/Fig4/4A/LLC/LLC 80 nM.tif]

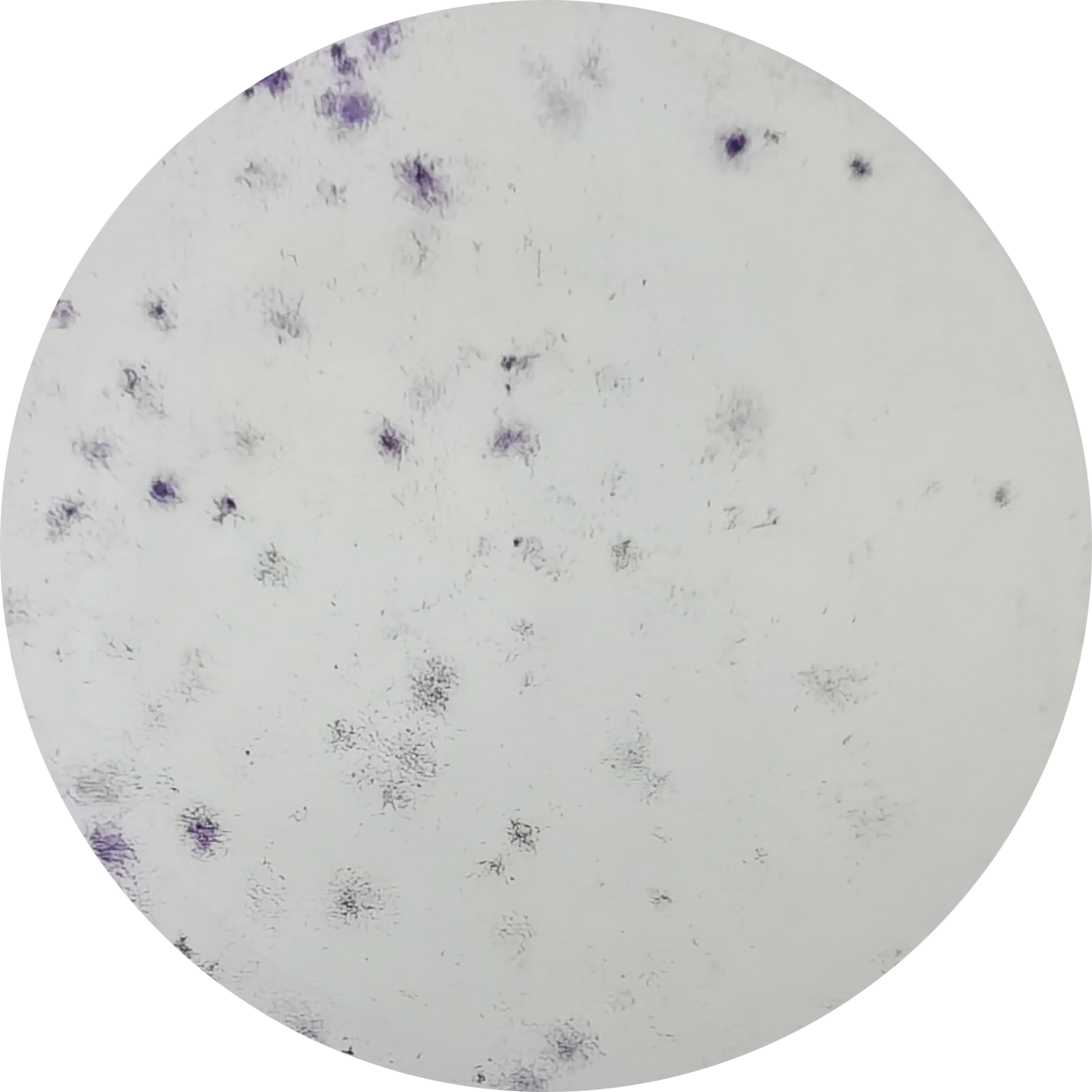

Supplement: Supplementary file 5 — Source Data Fig. 4 [file 44321_2024_51_MOESM5_ESM.zip › Fig-4/Fig4/4A/LLC/LLC 20 nM.tif]

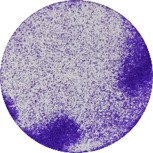

Supplement: Supplementary file 6 — Source Data Fig. 5 [file 44321_2024_51_MOESM6_ESM.zip › Fig-5/5A/A2780/1.tif]

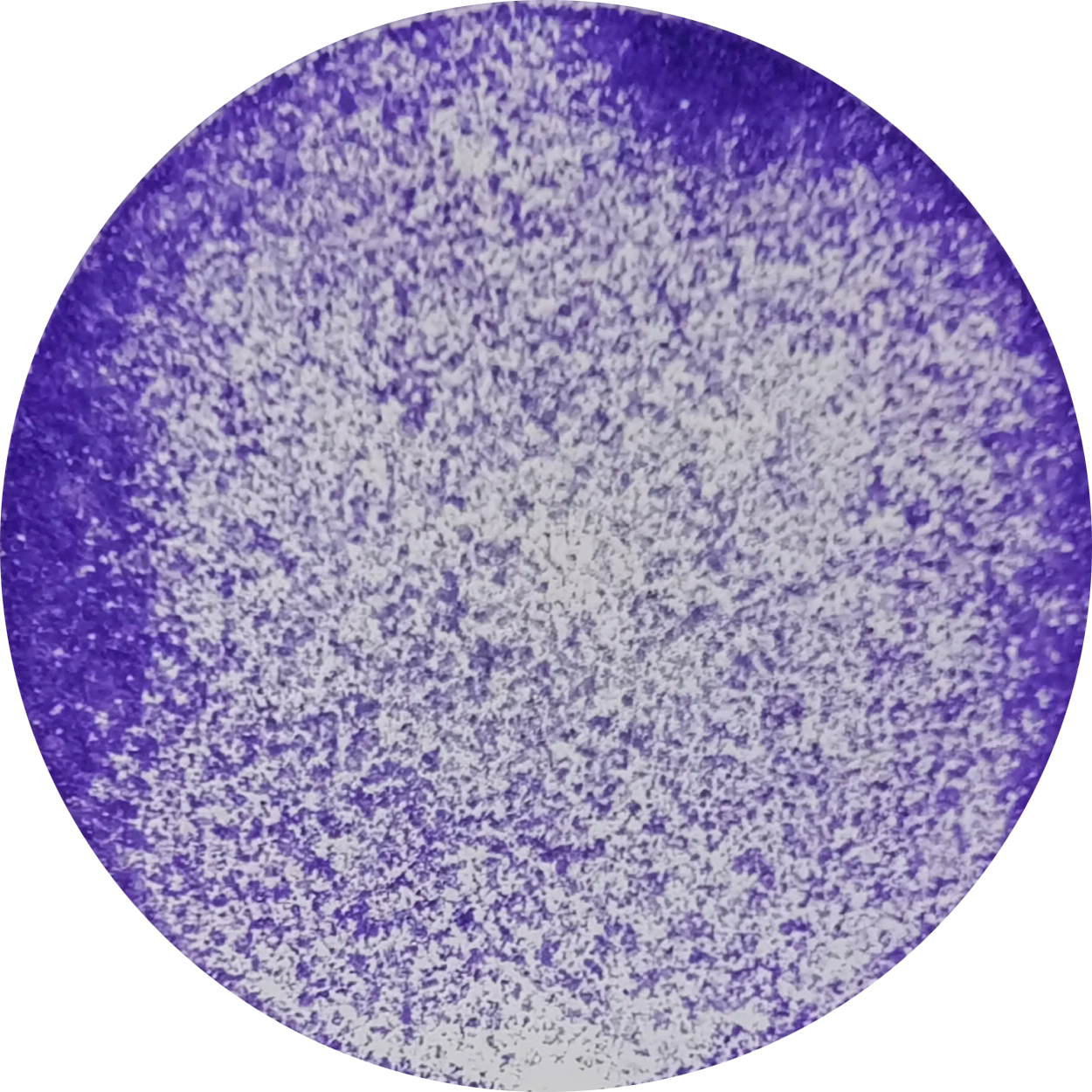

Supplement: Supplementary file 6 — Source Data Fig. 5 [file 44321_2024_51_MOESM6_ESM.zip › Fig-5/5A/A2780/2.tif]

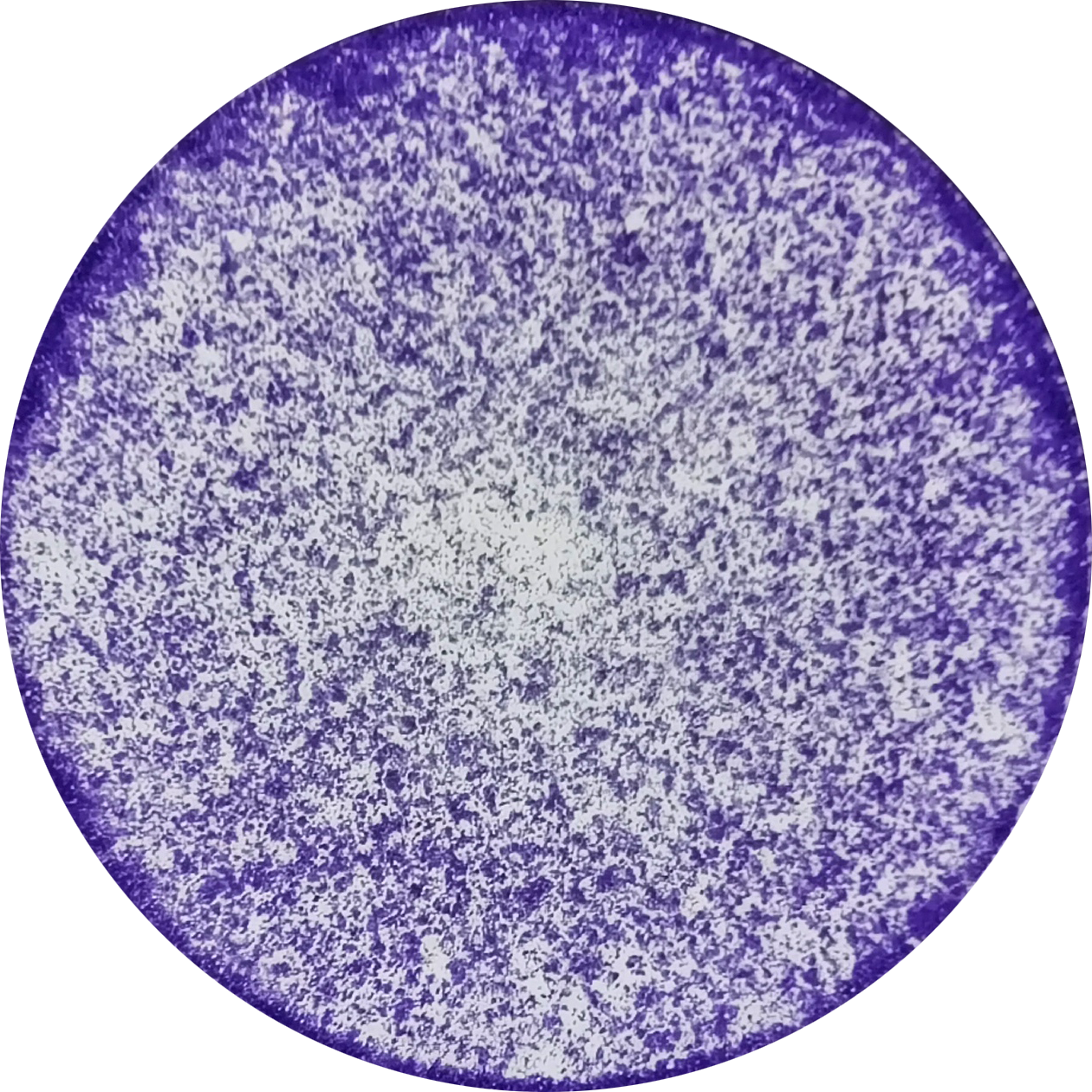

Supplement: Supplementary file 6 — Source Data Fig. 5 [file 44321_2024_51_MOESM6_ESM.zip › Fig-5/5A/A2780/3.tif]

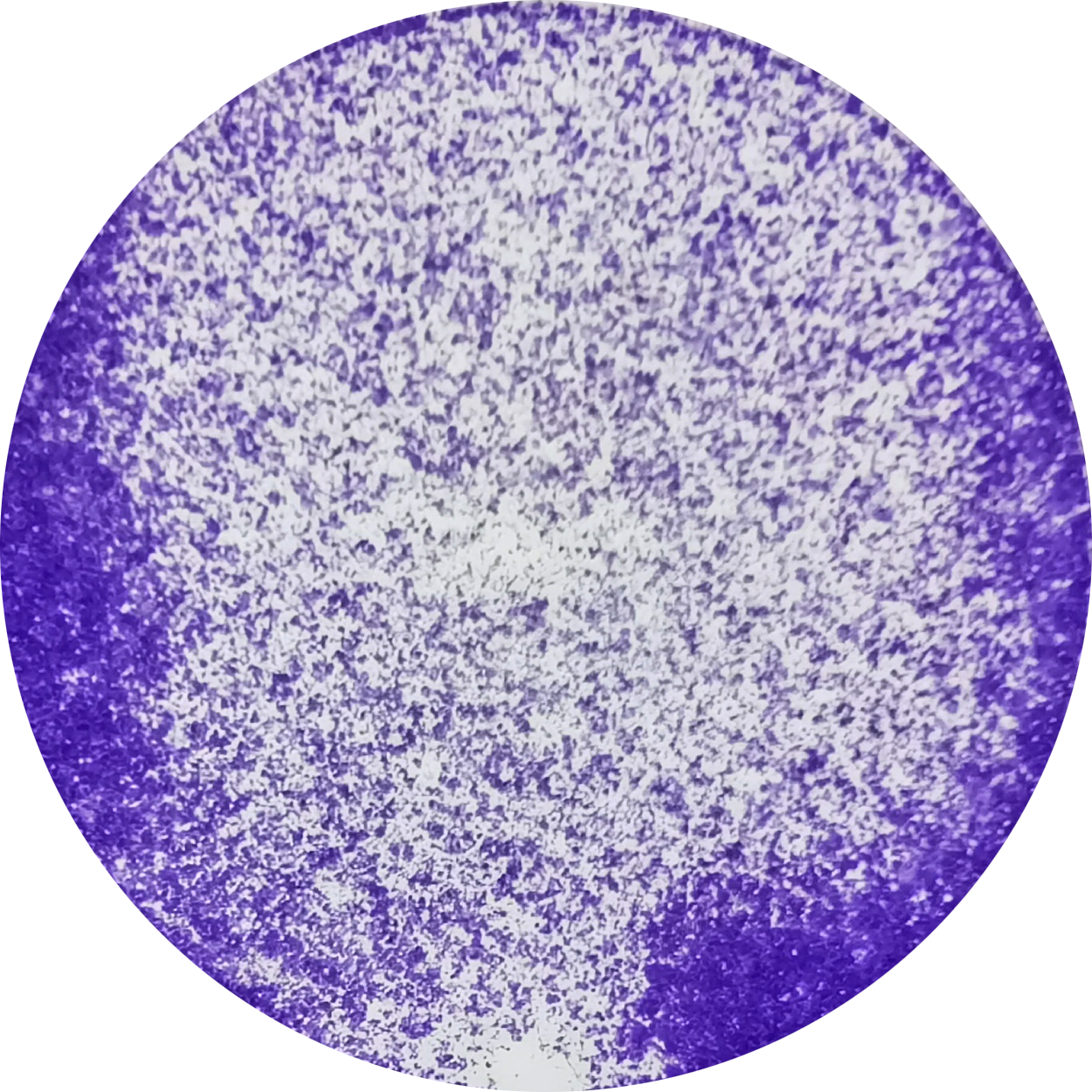

Supplement: Supplementary file 6 — Source Data Fig. 5 [file 44321_2024_51_MOESM6_ESM.zip › Fig-5/5A/A2780/4.tif]

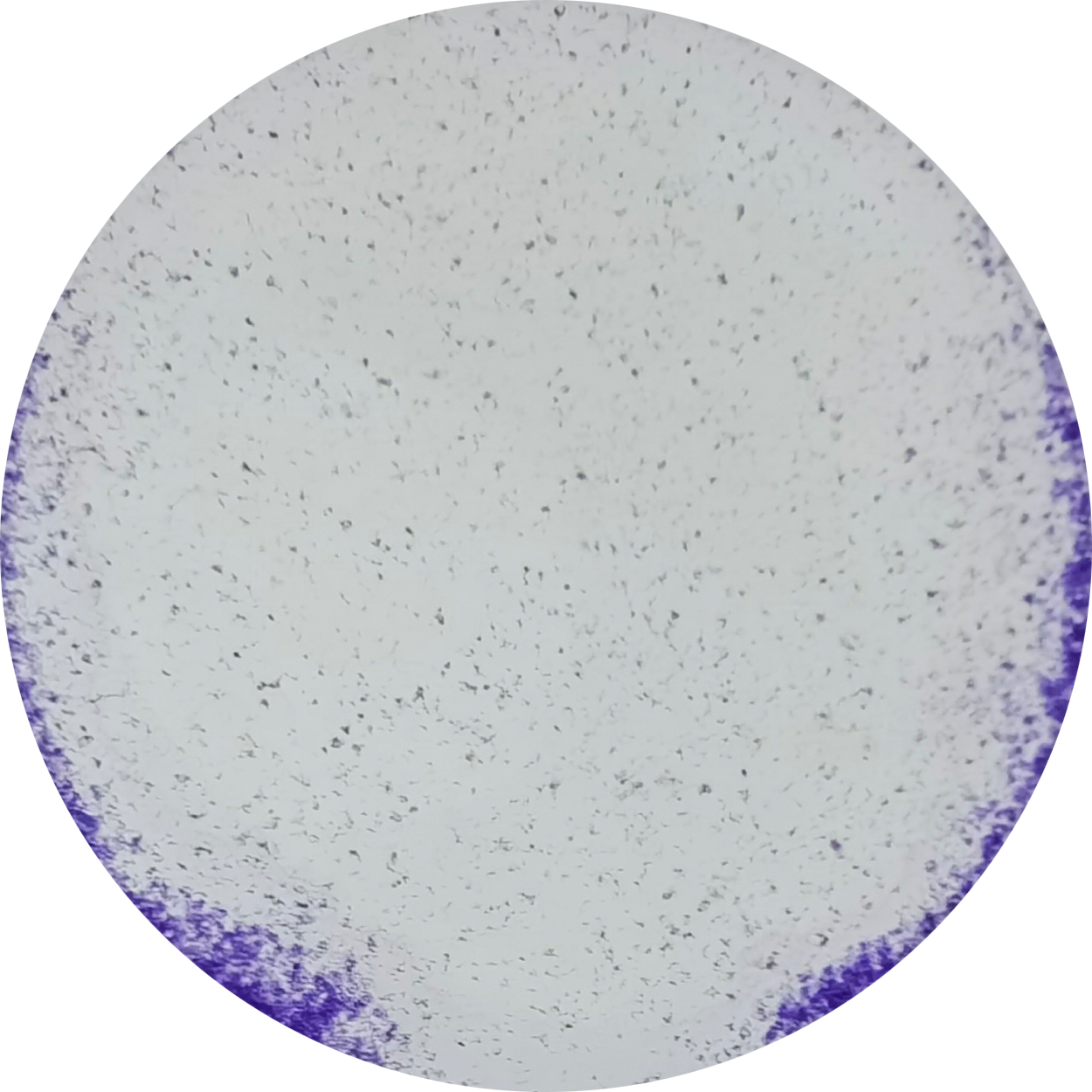

Supplement: Supplementary file 6 — Source Data Fig. 5 [file 44321_2024_51_MOESM6_ESM.zip › Fig-5/5A/A2780/5.tif]

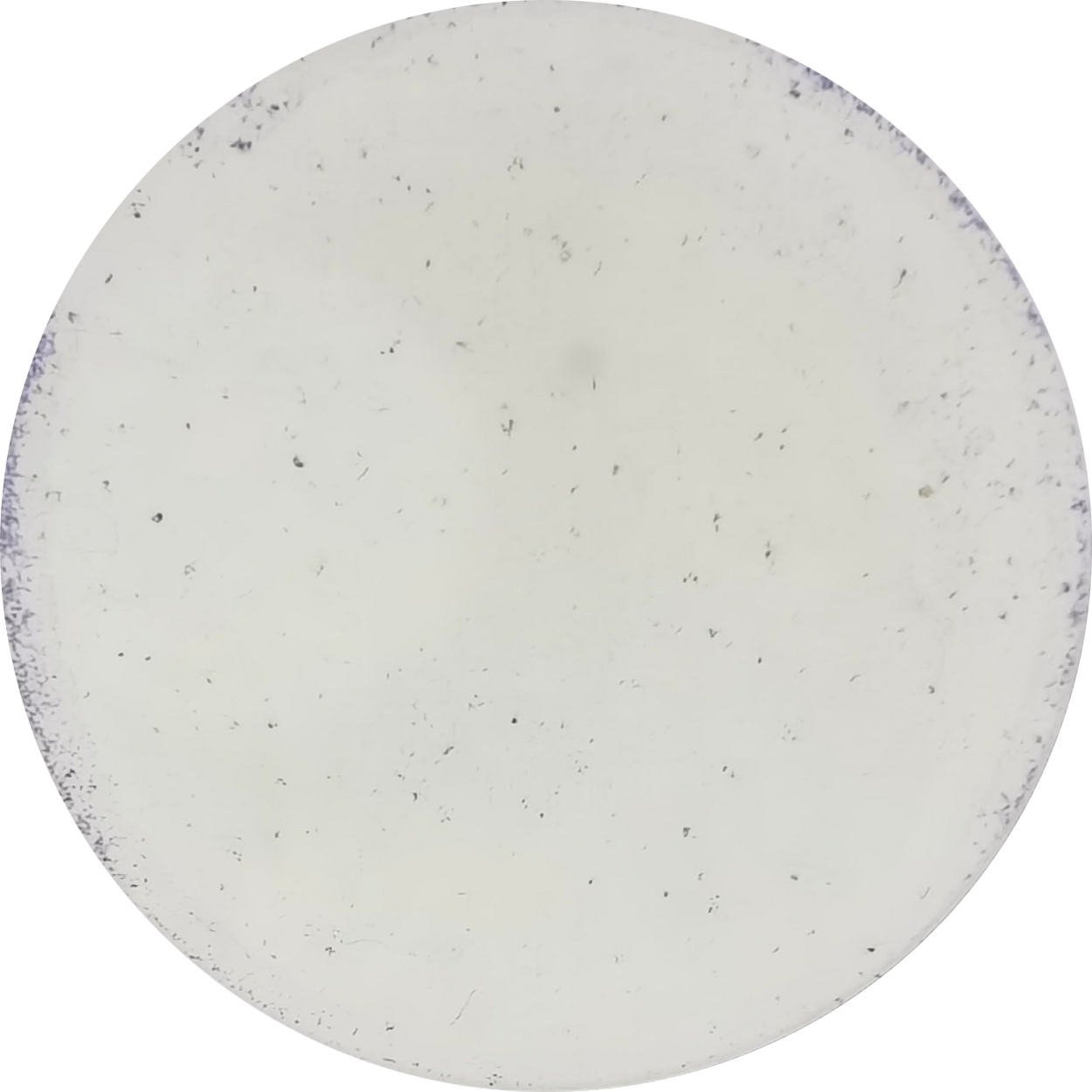

Supplement: Supplementary file 6 — Source Data Fig. 5 [file 44321_2024_51_MOESM6_ESM.zip › Fig-5/5A/A2780/6.tif]

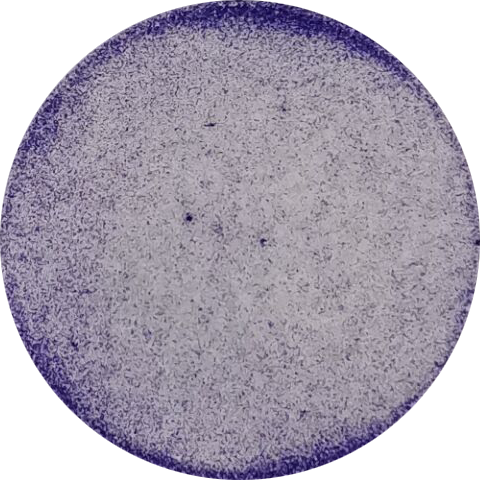

Supplement: Supplementary file 6 — Source Data Fig. 5 [file 44321_2024_51_MOESM6_ESM.zip › Fig-5/5A/CT-26/1.tif]

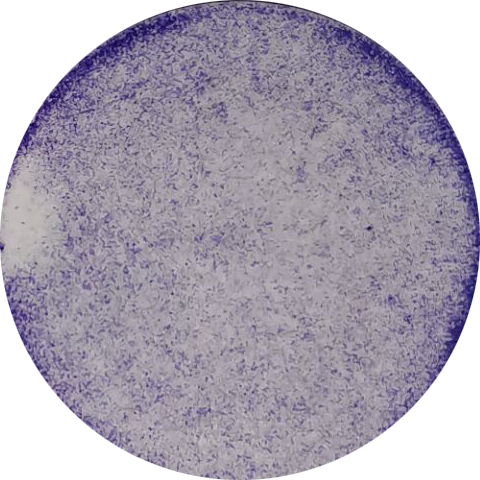

Supplement: Supplementary file 6 — Source Data Fig. 5 [file 44321_2024_51_MOESM6_ESM.zip › Fig-5/5A/CT-26/2.tif]

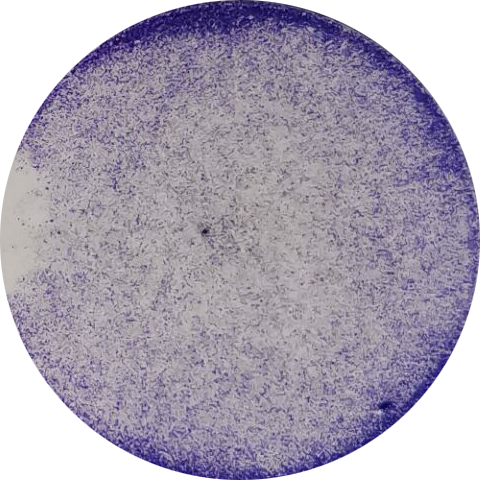

Supplement: Supplementary file 6 — Source Data Fig. 5 [file 44321_2024_51_MOESM6_ESM.zip › Fig-5/5A/CT-26/3.tif]

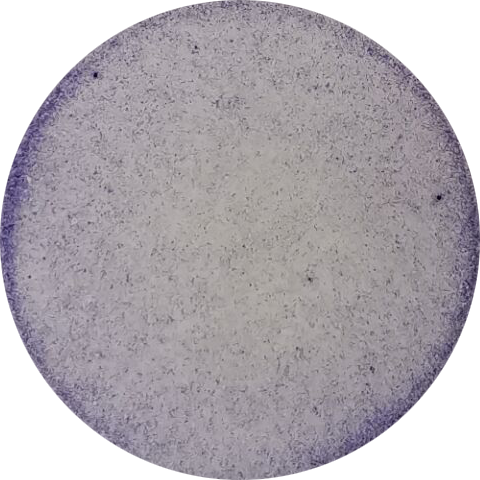

Supplement: Supplementary file 6 — Source Data Fig. 5 [file 44321_2024_51_MOESM6_ESM.zip › Fig-5/5A/CT-26/4.tif]

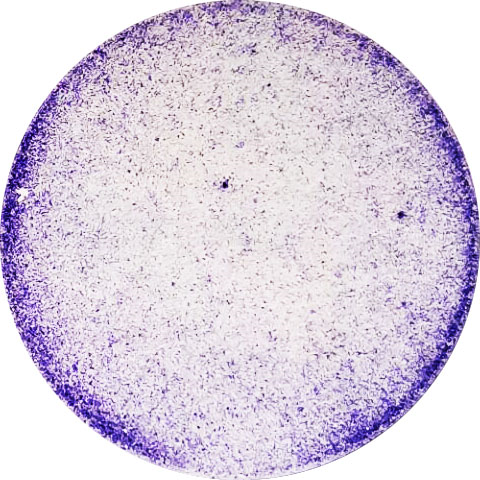

Supplement: Supplementary file 6 — Source Data Fig. 5 [file 44321_2024_51_MOESM6_ESM.zip › Fig-5/5A/CT-26/5.tif]

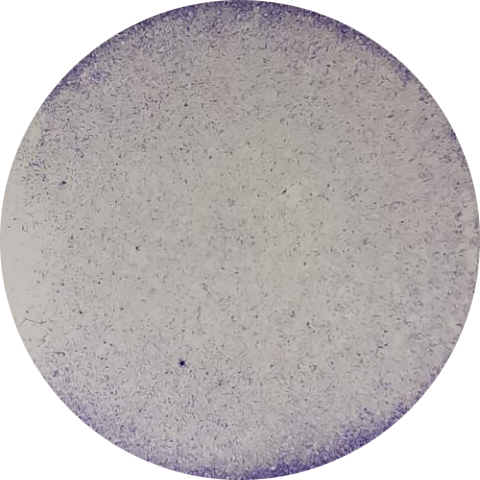

Supplement: Supplementary file 6 — Source Data Fig. 5 [file 44321_2024_51_MOESM6_ESM.zip › Fig-5/5A/CT-26/6.tif]

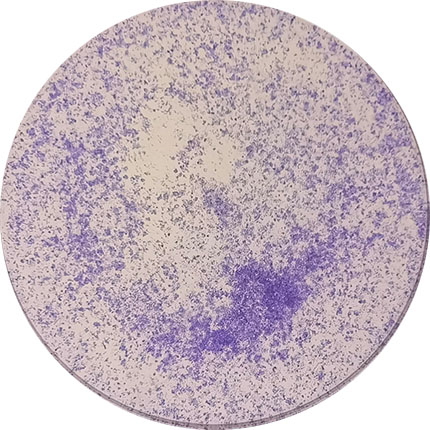

Supplement: Supplementary file 6 — Source Data Fig. 5 [file 44321_2024_51_MOESM6_ESM.zip › Fig-5/5A/HCT-116/1.tif]

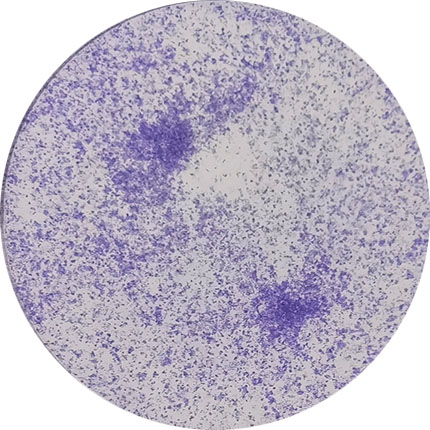

Supplement: Supplementary file 6 — Source Data Fig. 5 [file 44321_2024_51_MOESM6_ESM.zip › Fig-5/5A/HCT-116/2.tif]

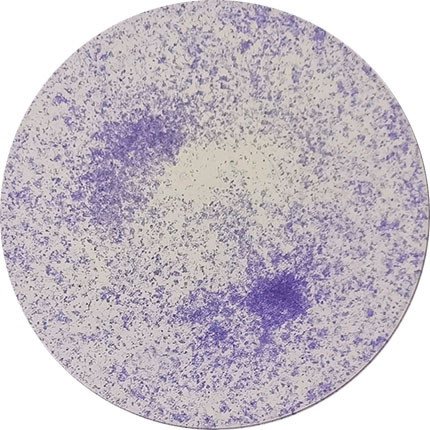

Supplement: Supplementary file 6 — Source Data Fig. 5 [file 44321_2024_51_MOESM6_ESM.zip › Fig-5/5A/HCT-116/3.tif]

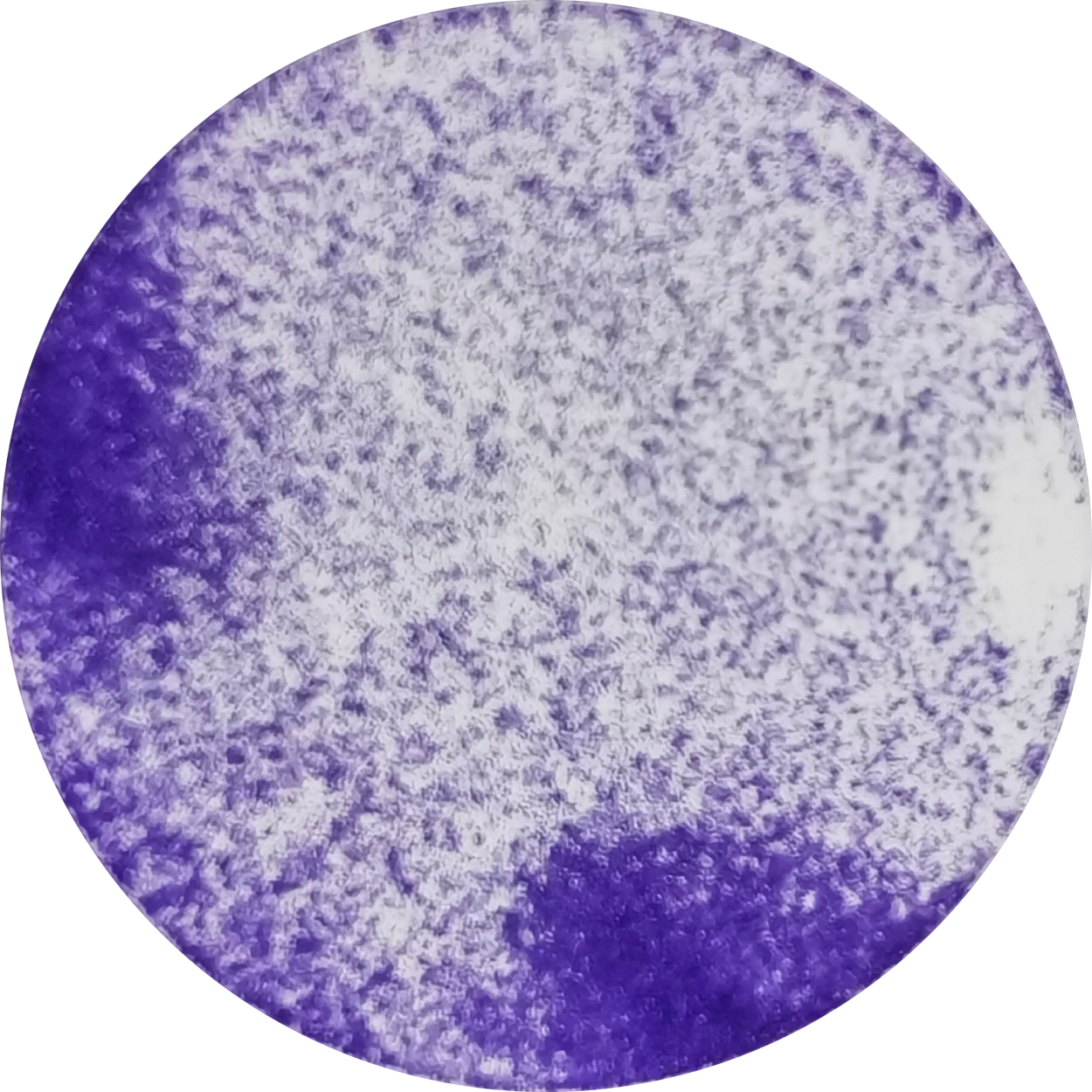

Supplement: Supplementary file 6 — Source Data Fig. 5 [file 44321_2024_51_MOESM6_ESM.zip › Fig-5/5A/HCT-116/4.tif]

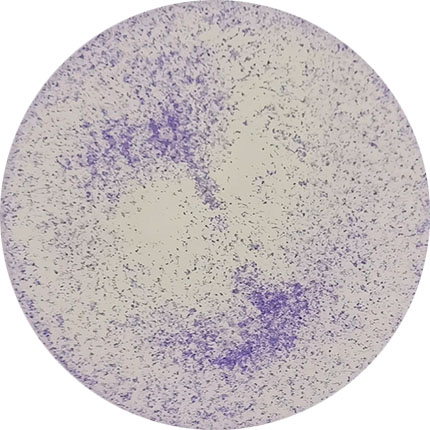

Supplement: Supplementary file 6 — Source Data Fig. 5 [file 44321_2024_51_MOESM6_ESM.zip › Fig-5/5A/HCT-116/5.tif]

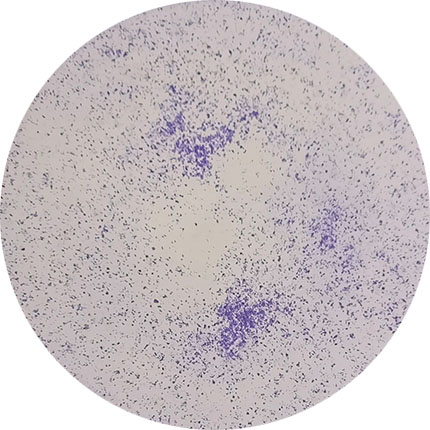

Supplement: Supplementary file 6 — Source Data Fig. 5 [file 44321_2024_51_MOESM6_ESM.zip › Fig-5/5A/HCT-116/6.tif]

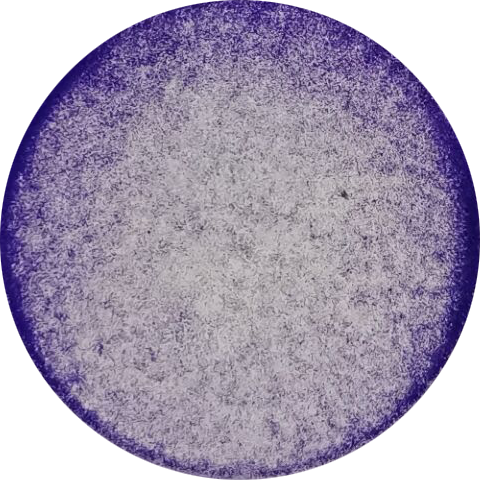

Supplement: Supplementary file 6 — Source Data Fig. 5 [file 44321_2024_51_MOESM6_ESM.zip › Fig-5/5A/LLC/1.tif]

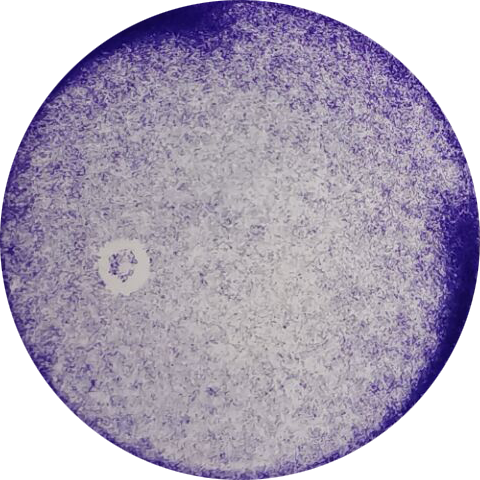

Supplement: Supplementary file 6 — Source Data Fig. 5 [file 44321_2024_51_MOESM6_ESM.zip › Fig-5/5A/LLC/2.tif]

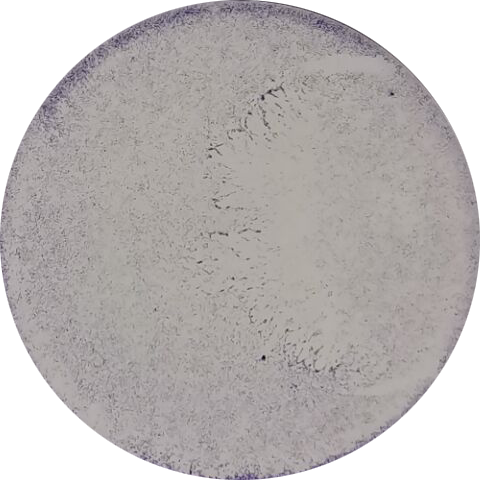

Supplement: Supplementary file 6 — Source Data Fig. 5 [file 44321_2024_51_MOESM6_ESM.zip › Fig-5/5A/LLC/5.tif]

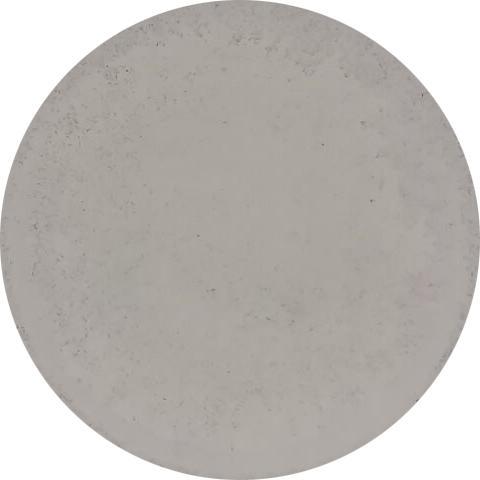

Supplement: Supplementary file 6 — Source Data Fig. 5 [file 44321_2024_51_MOESM6_ESM.zip › Fig-5/5A/LLC/6.tif]

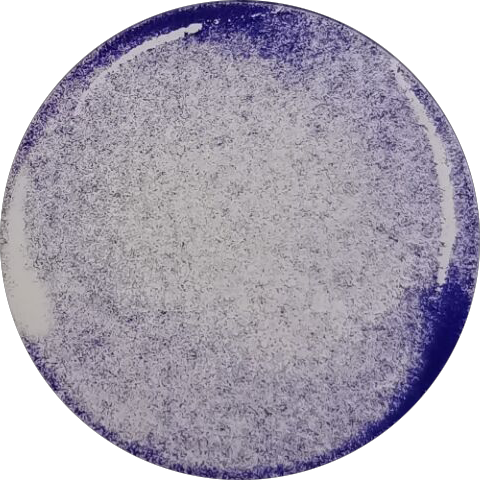

Supplement: Supplementary file 6 — Source Data Fig. 5 [file 44321_2024_51_MOESM6_ESM.zip › Fig-5/5A/LLC/4.tif]

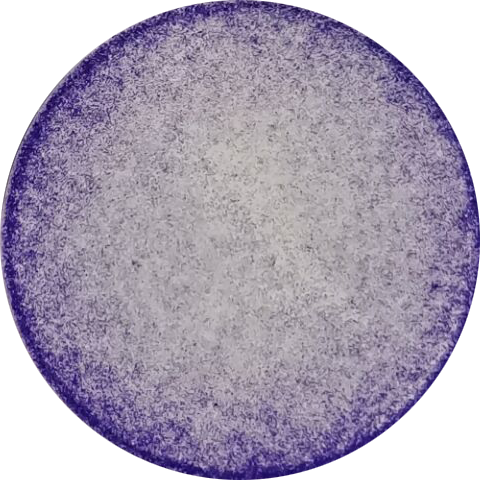

Supplement: Supplementary file 6 — Source Data Fig. 5 [file 44321_2024_51_MOESM6_ESM.zip › Fig-5/5A/LLC/3.tif]

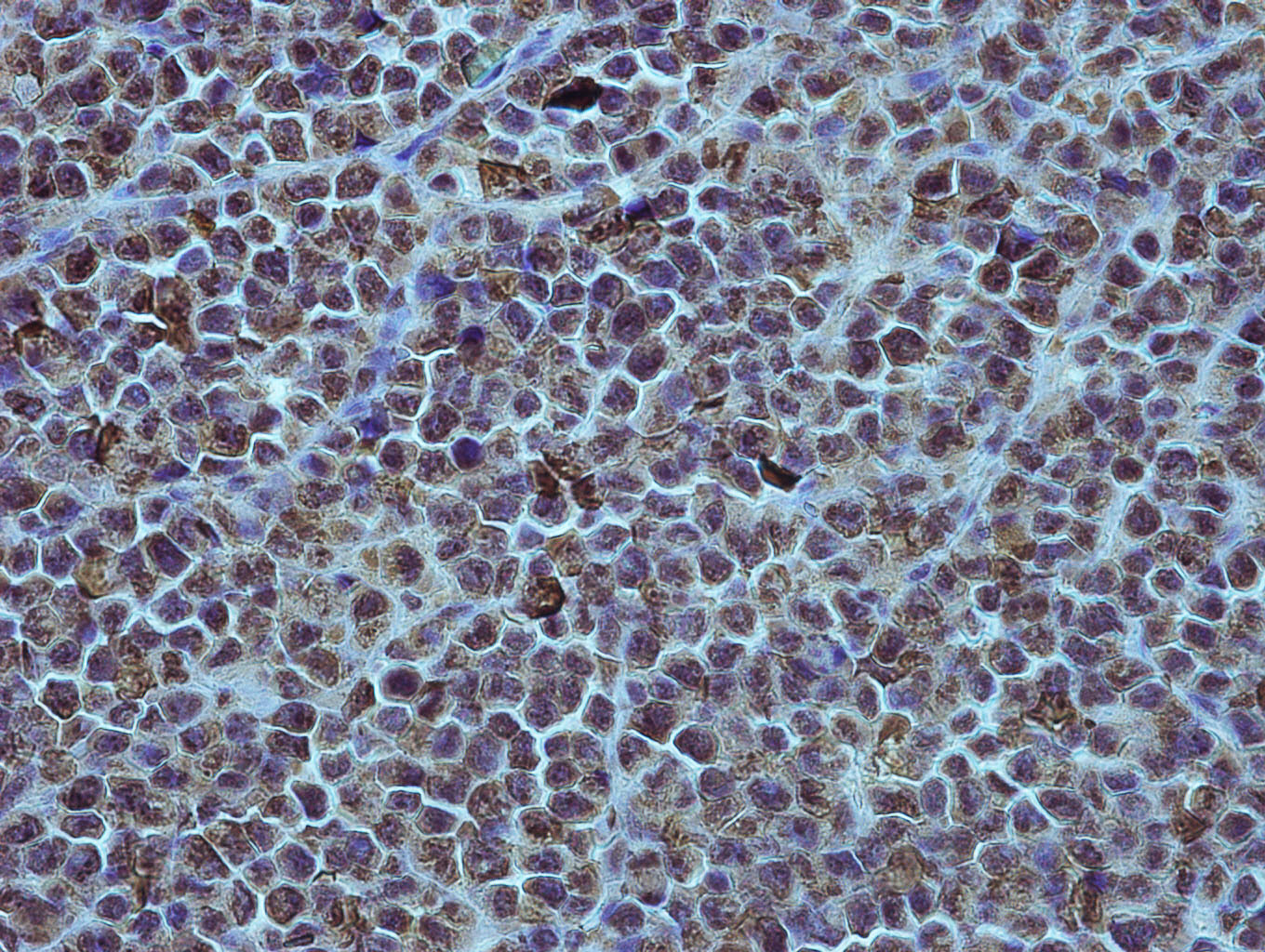

Supplement: Supplementary file 10 — Source Data Fig. 6-EFGH [file 44321_2024_51_MOESM10_ESM.zip › Fig-6-EFGH/6 F/FIG6-F-KI67/A20 Ctrl.tif]

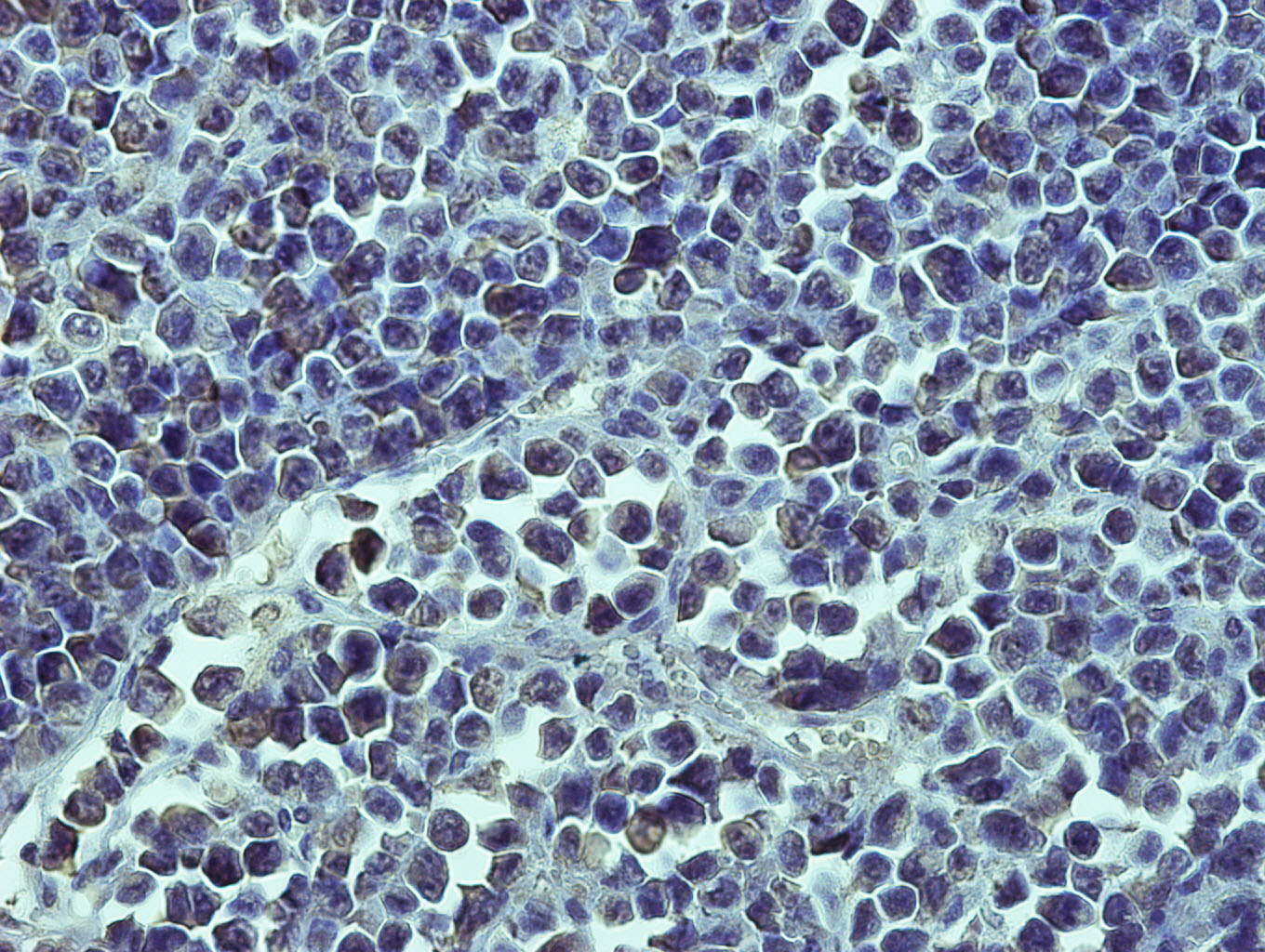

Supplement: Supplementary file 10 — Source Data Fig. 6-EFGH [file 44321_2024_51_MOESM10_ESM.zip › Fig-6-EFGH/6 F/FIG6-F-KI67/A20 LZ90.tif]

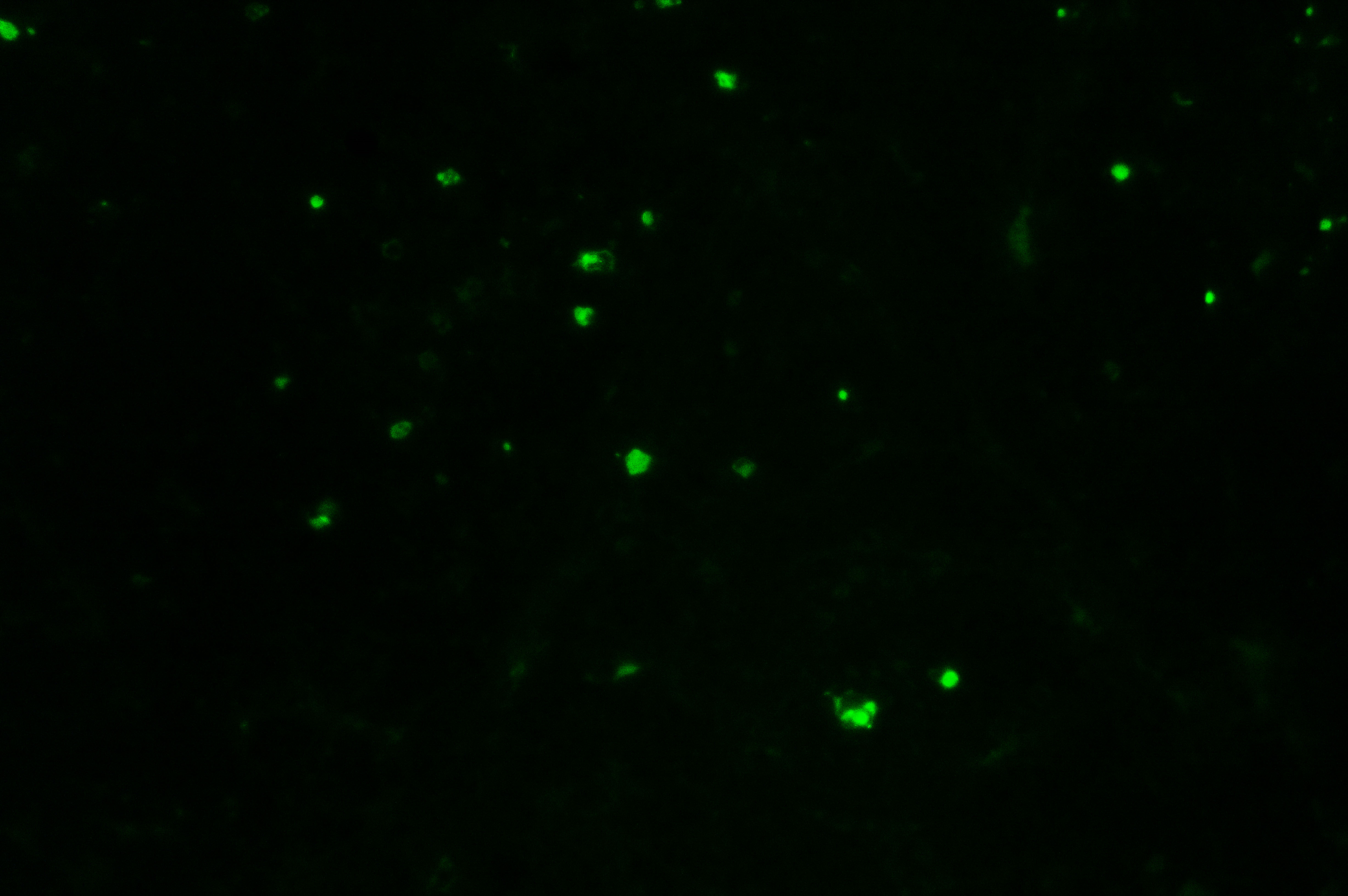

Supplement: Supplementary file 10 — Source Data Fig. 6-EFGH [file 44321_2024_51_MOESM10_ESM.zip › Fig-6-EFGH/6 F/Fig6-F-Tunel/Tunel Ctrl.tif]

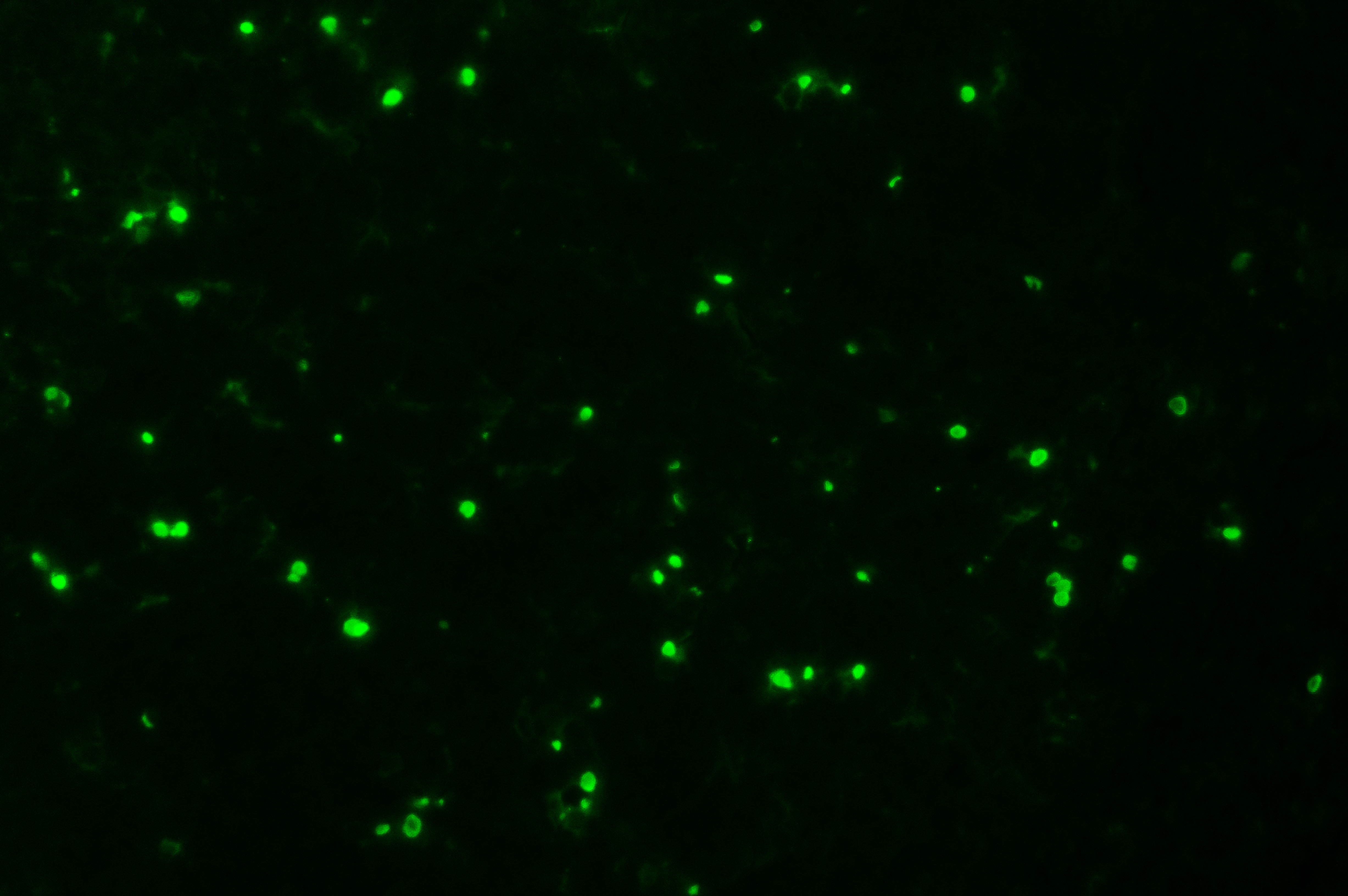

Supplement: Supplementary file 10 — Source Data Fig. 6-EFGH [file 44321_2024_51_MOESM10_ESM.zip › Fig-6-EFGH/6 F/Fig6-F-Tunel/Tunel LZ90.tif]

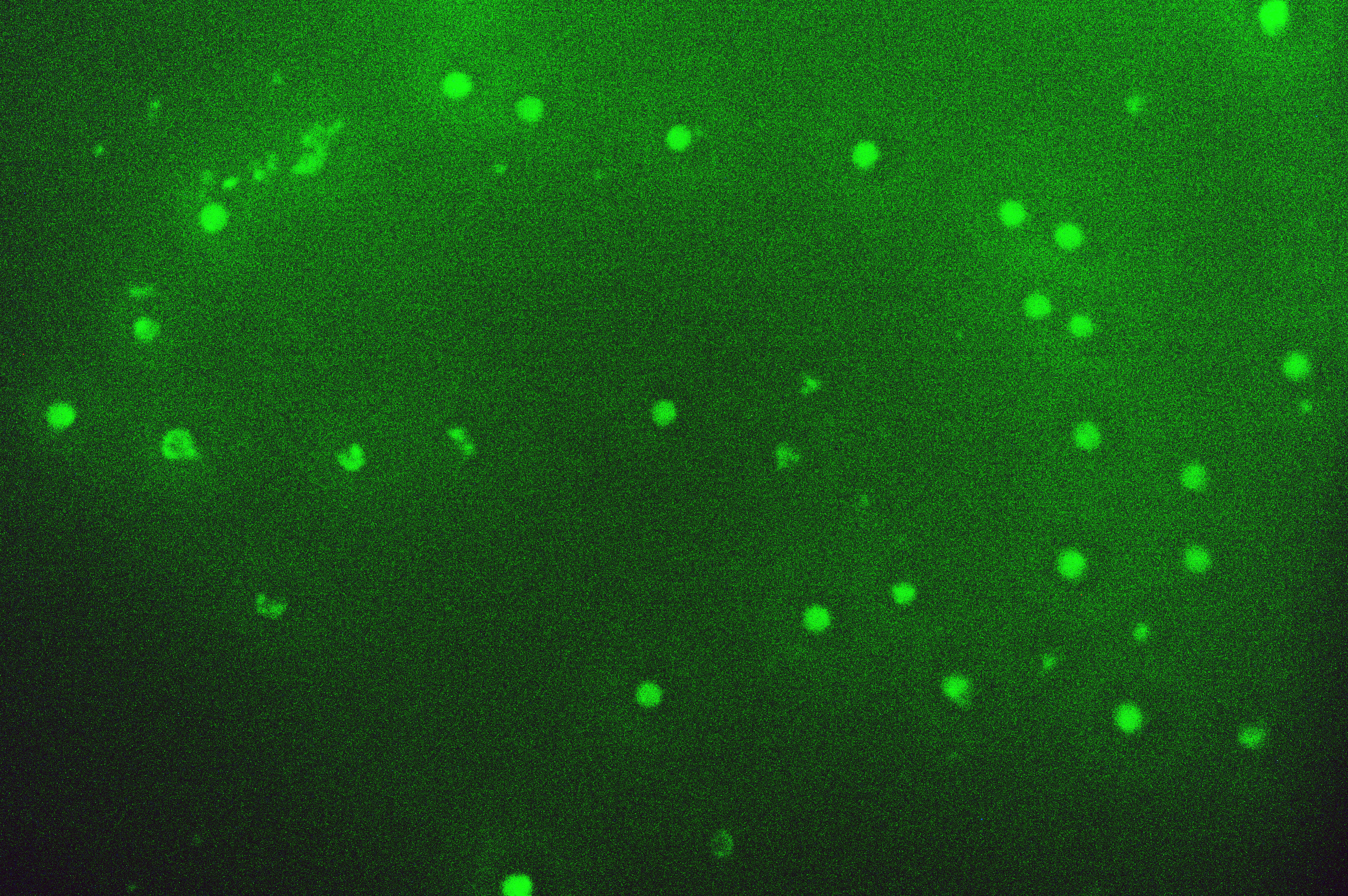

Supplement: Supplementary file 10 — Source Data Fig. 6-EFGH [file 44321_2024_51_MOESM10_ESM.zip › Fig-6-EFGH/6 H/CFSE D6 Ctrl.tif]

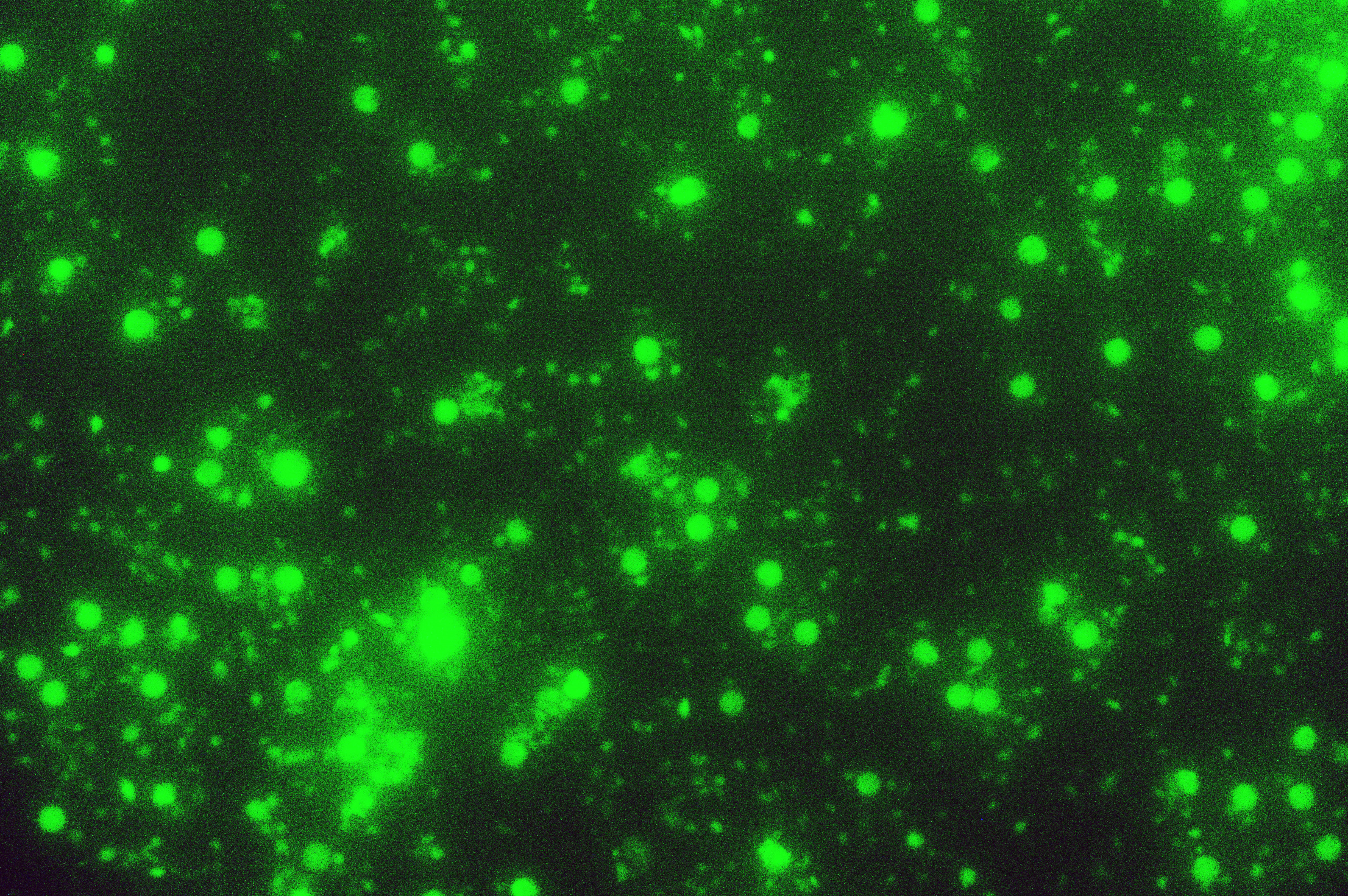

Supplement: Supplementary file 10 — Source Data Fig. 6-EFGH [file 44321_2024_51_MOESM10_ESM.zip › Fig-6-EFGH/6 H/CFSE D6 LZ90.tif]

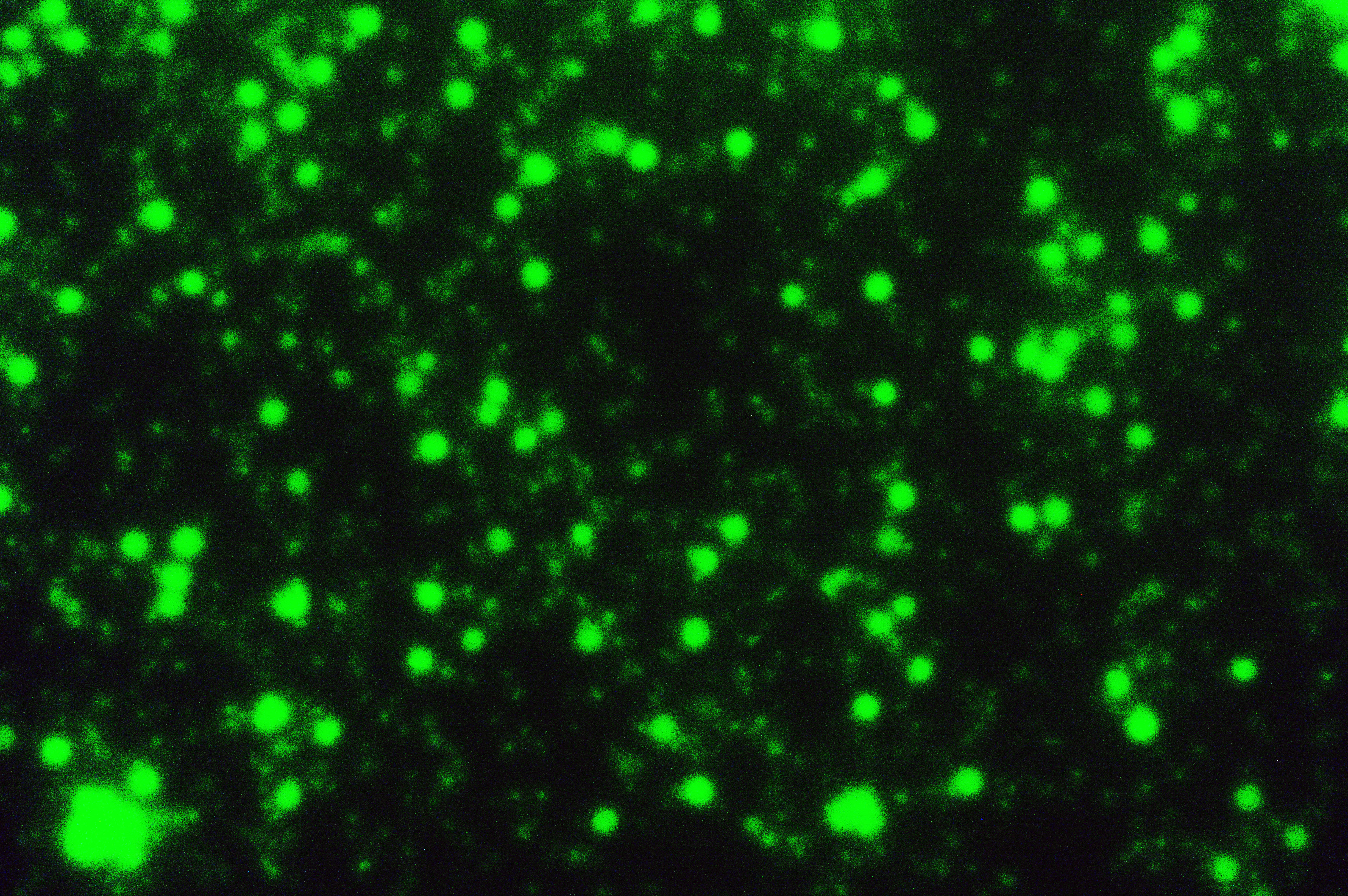

Supplement: Supplementary file 10 — Source Data Fig. 6-EFGH [file 44321_2024_51_MOESM10_ESM.zip › Fig-6-EFGH/6 H/CFSE D9 Ctrl.tif]

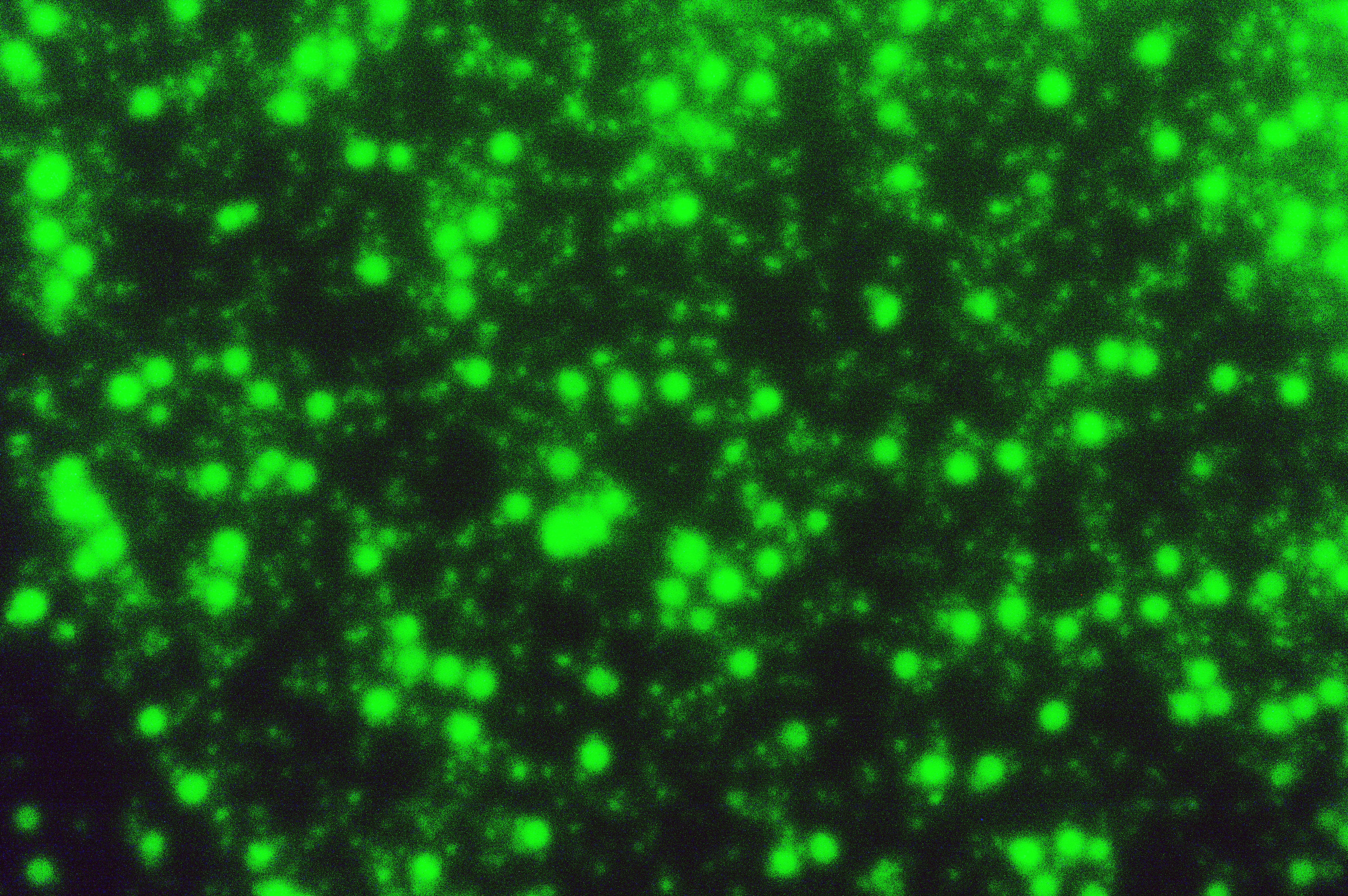

Supplement: Supplementary file 10 — Source Data Fig. 6-EFGH [file 44321_2024_51_MOESM10_ESM.zip › Fig-6-EFGH/6 H/CFSE D9 LZ90.tif]

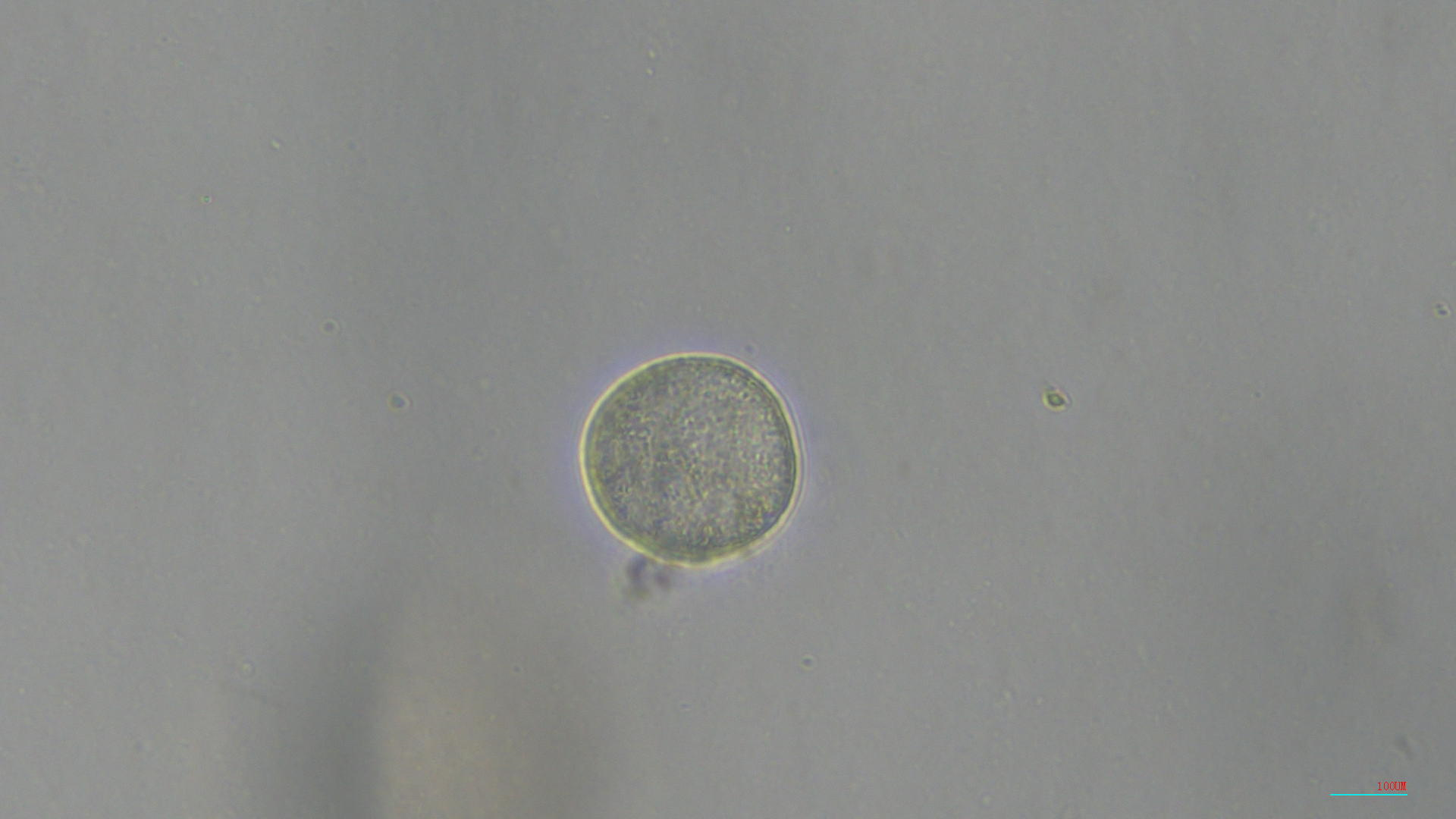

Supplement: Supplementary file 10 — Source Data Fig. 6-EFGH [file 44321_2024_51_MOESM10_ESM.zip › Fig-6-EFGH/6 G/Ctrl-D0.tif]

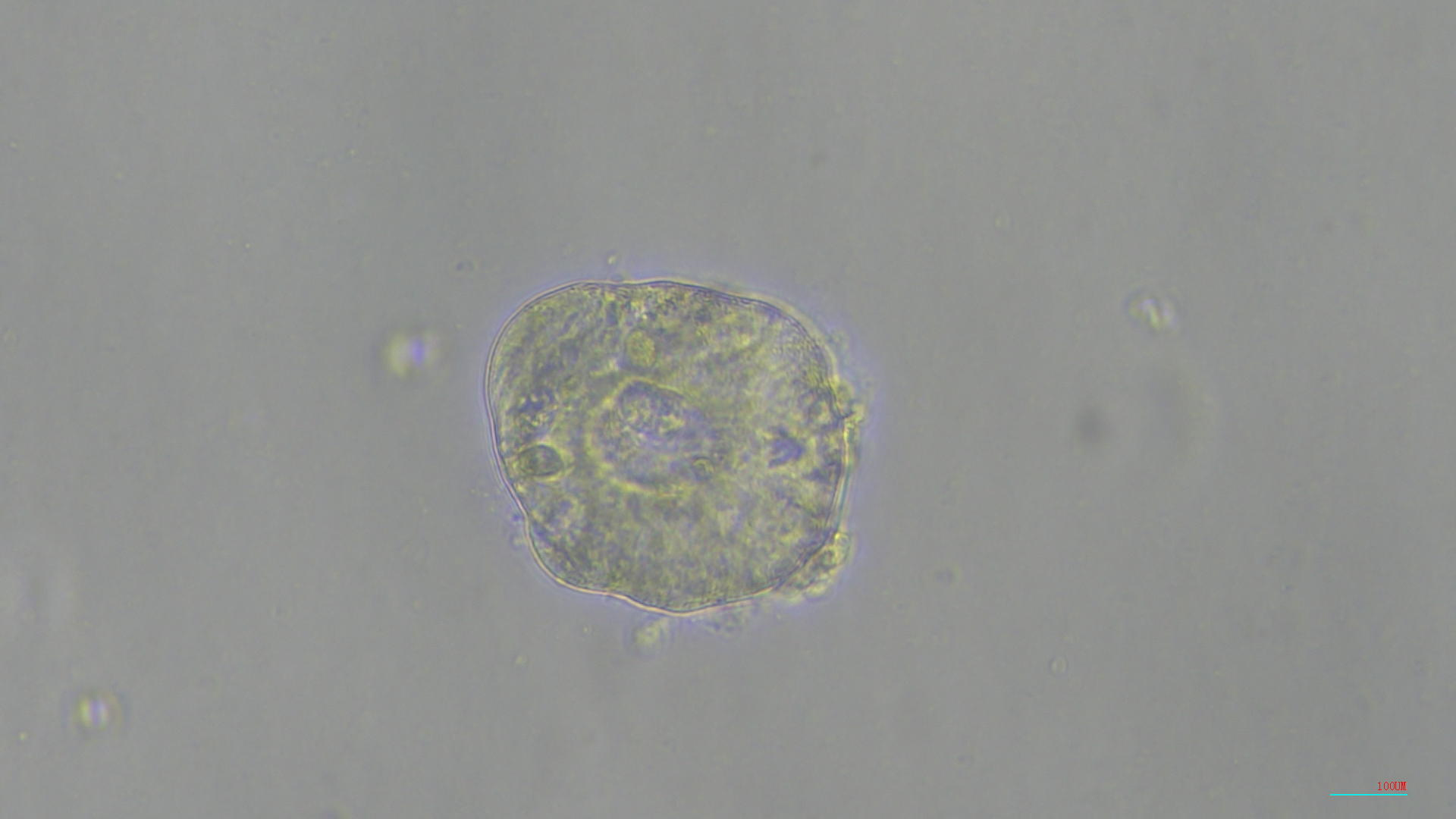

Supplement: Supplementary file 10 — Source Data Fig. 6-EFGH [file 44321_2024_51_MOESM10_ESM.zip › Fig-6-EFGH/6 G/Ctrl-D9.tif]

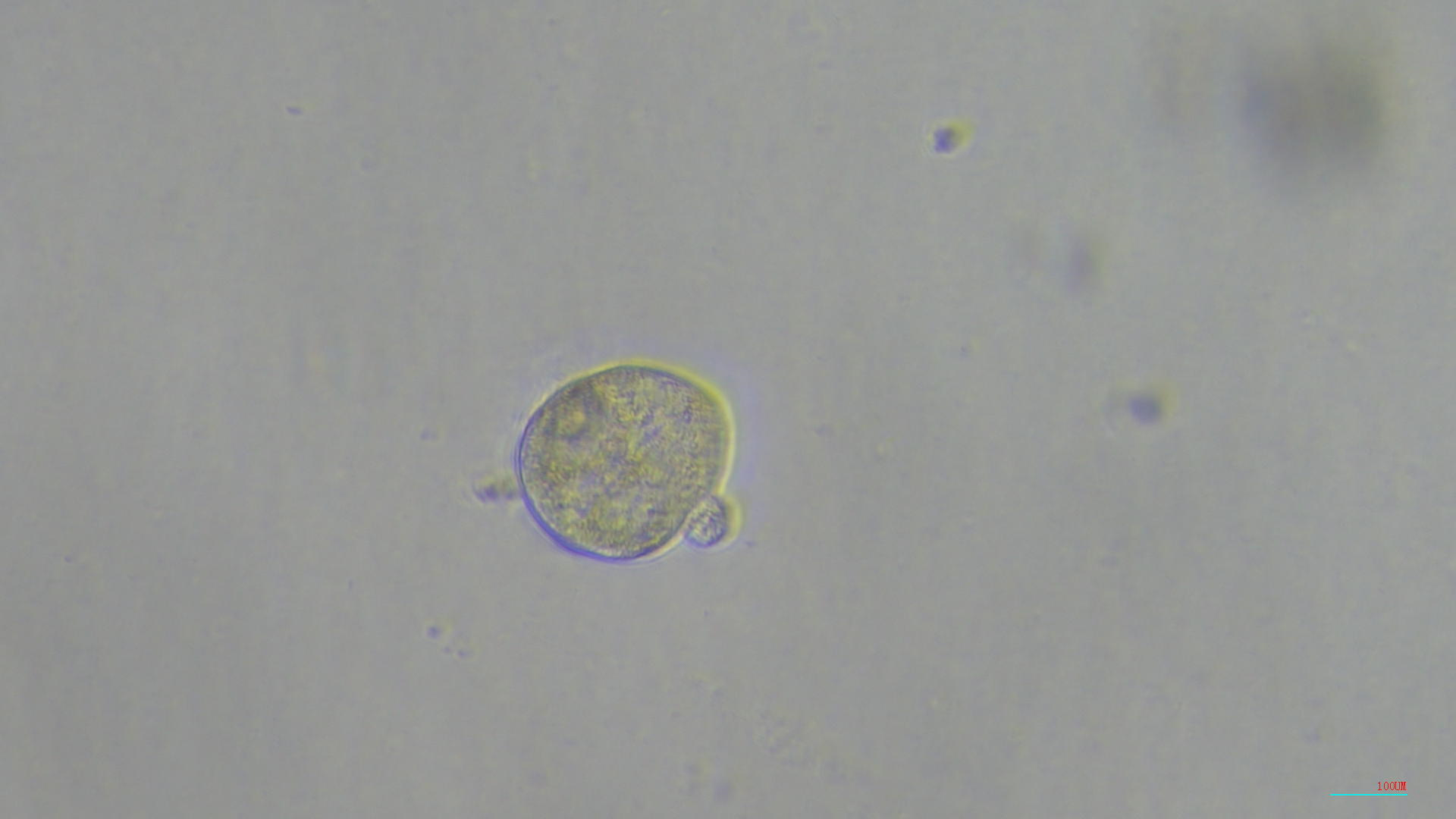

Supplement: Supplementary file 10 — Source Data Fig. 6-EFGH [file 44321_2024_51_MOESM10_ESM.zip › Fig-6-EFGH/6 G/LZ90-D0.tif]

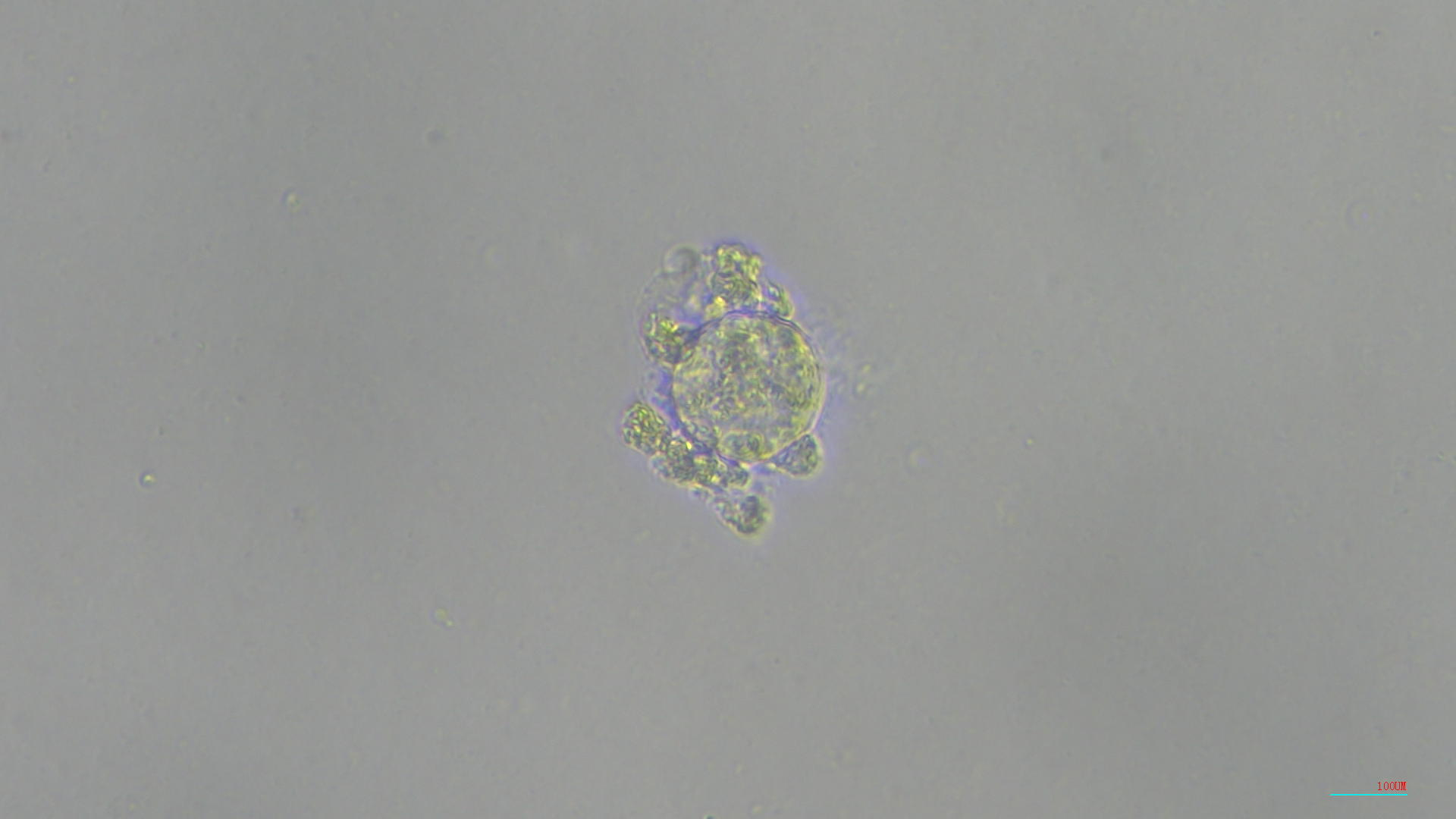

Supplement: Supplementary file 10 — Source Data Fig. 6-EFGH [file 44321_2024_51_MOESM10_ESM.zip › Fig-6-EFGH/6 G/LZ90-D9.tif]

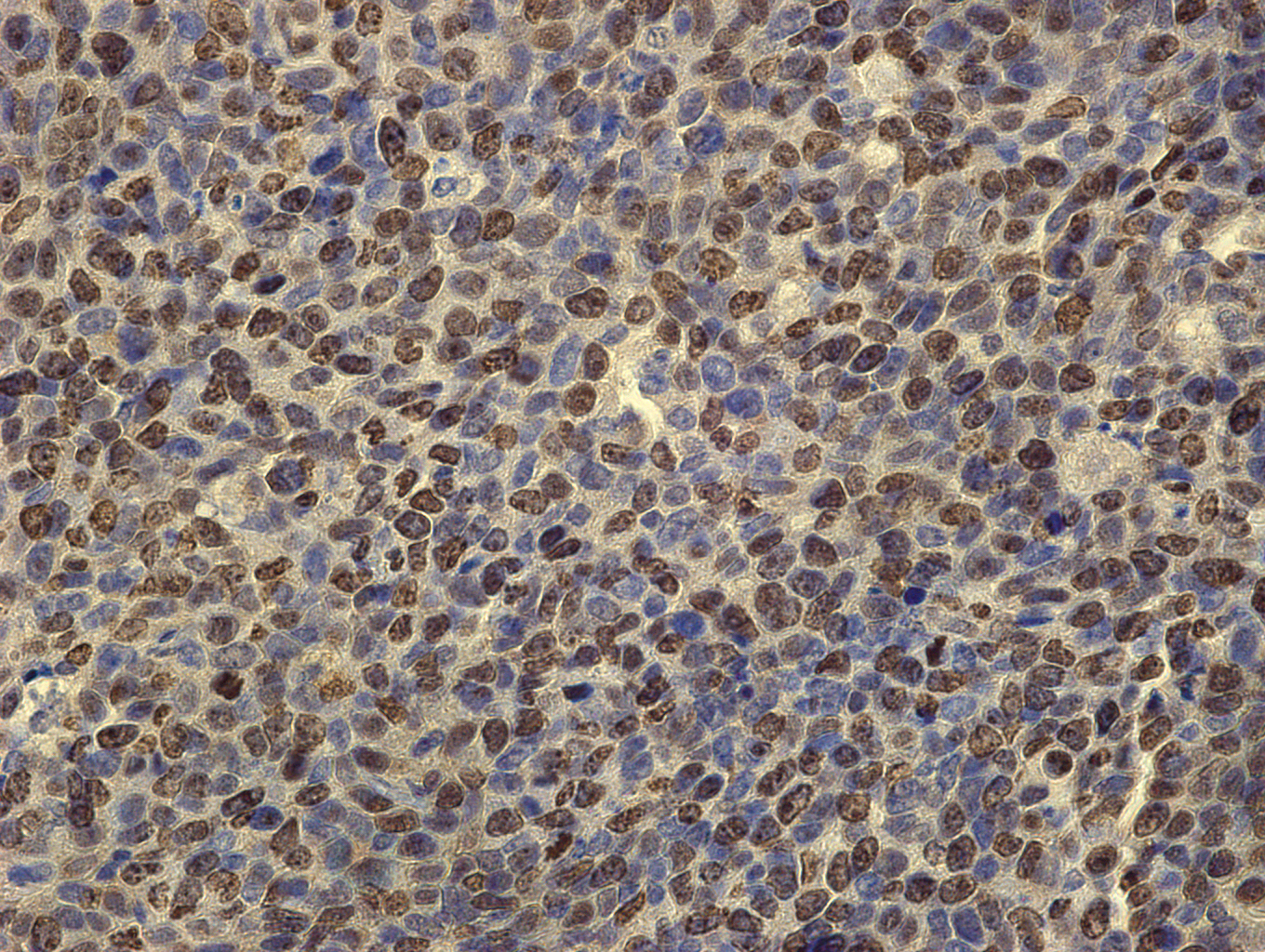

Supplement: Supplementary file 11 — Source Data Fig. 7 [file 44321_2024_51_MOESM11_ESM.zip › Fig-7/7F/7F-KI67/LLC Ctrl.tif]

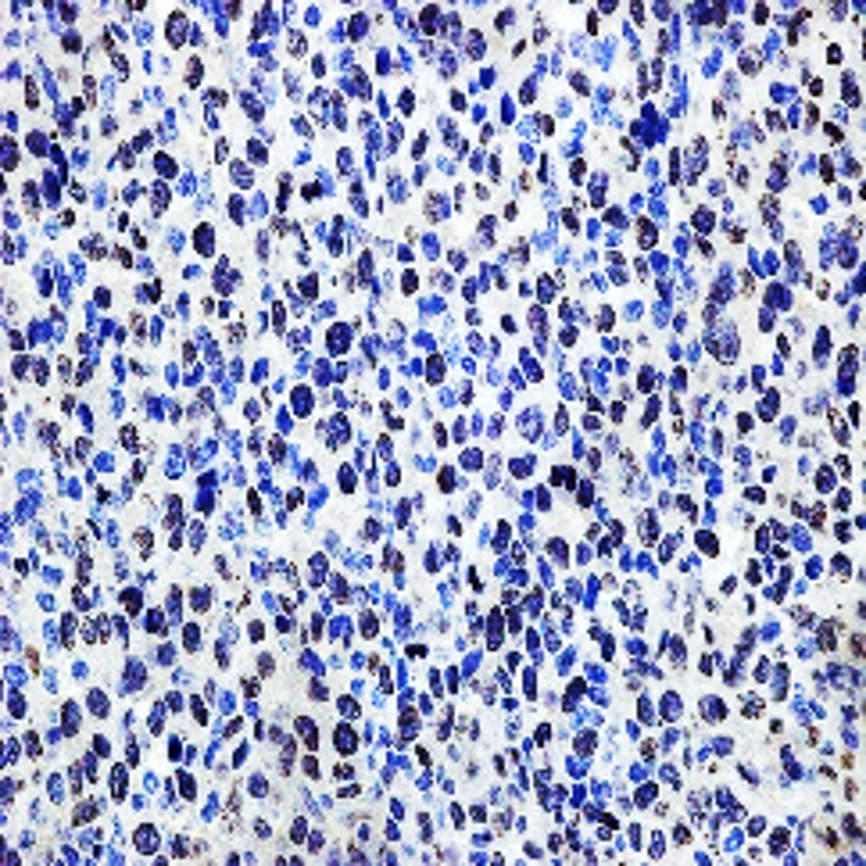

Supplement: Supplementary file 11 — Source Data Fig. 7 [file 44321_2024_51_MOESM11_ESM.zip › Fig-7/7F/7F-KI67/KI67 LZ90.tif]

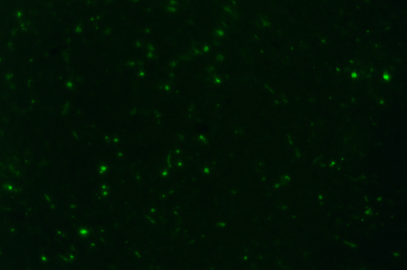

Supplement: Supplementary file 11 — Source Data Fig. 7 [file 44321_2024_51_MOESM11_ESM.zip › Fig-7/7F/7F-Tunel/7F Tunel Ctrl.tif]

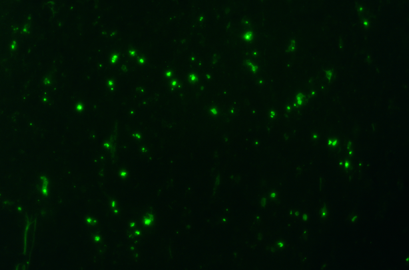

Supplement: Supplementary file 11 — Source Data Fig. 7 [file 44321_2024_51_MOESM11_ESM.zip › Fig-7/7F/7F-Tunel/7F Tunel LZ90.tif]

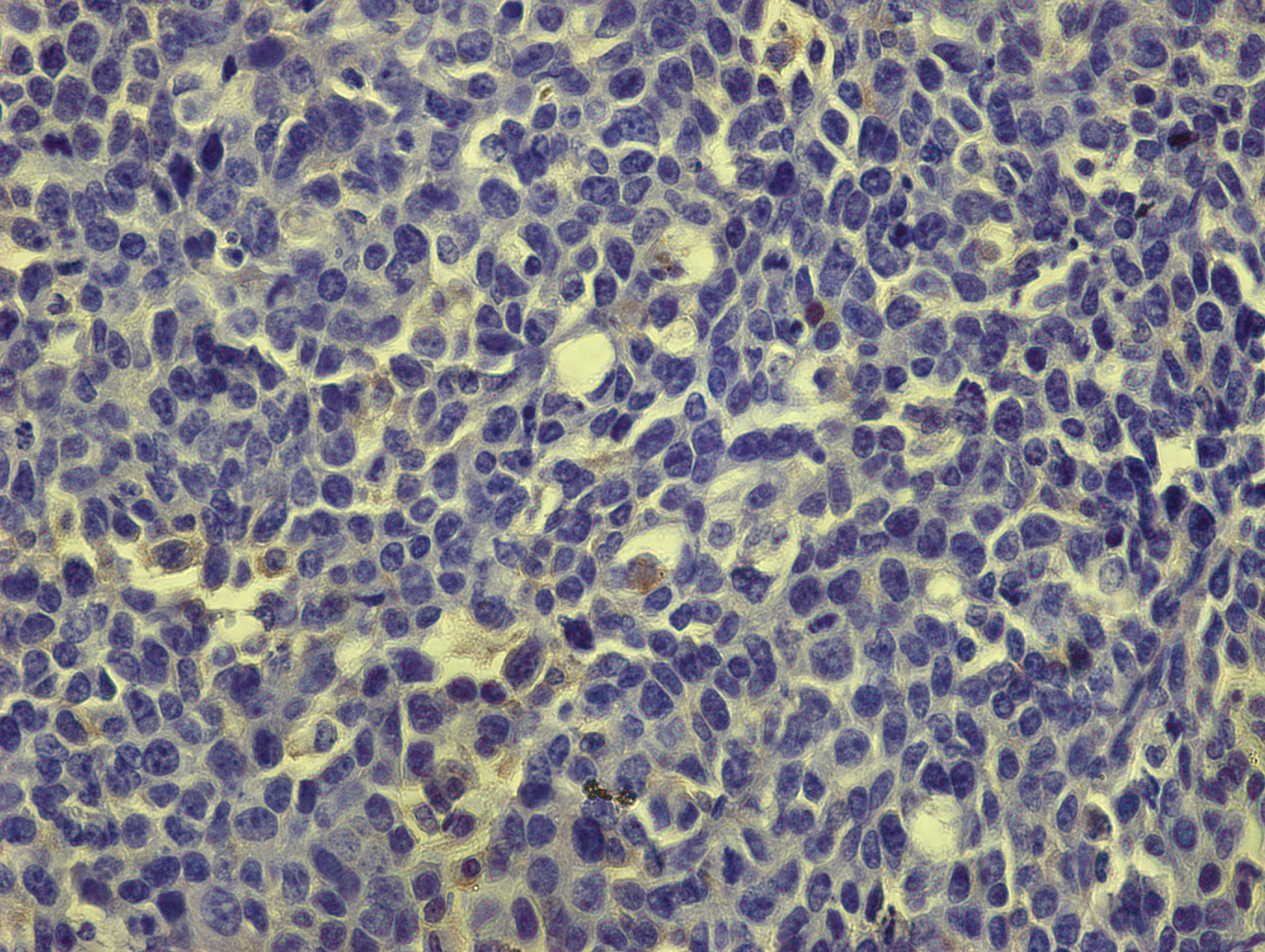

Supplement: Supplementary file 11 — Source Data Fig. 7 [file 44321_2024_51_MOESM11_ESM.zip › Fig-7/7E/CD8 Ctrl.tif]

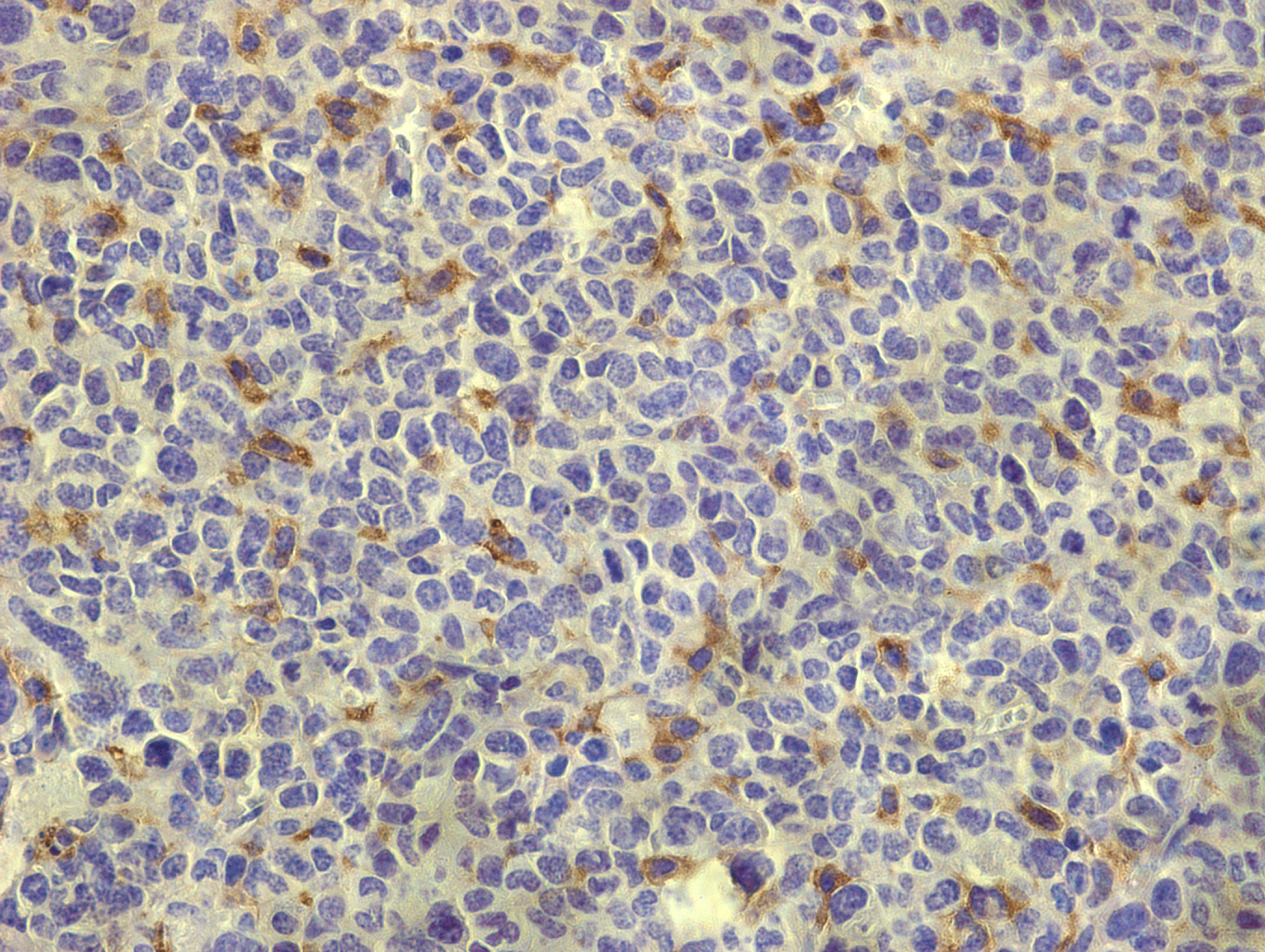

Supplement: Supplementary file 11 — Source Data Fig. 7 [file 44321_2024_51_MOESM11_ESM.zip › Fig-7/7E/CD8 LZ90.tif]

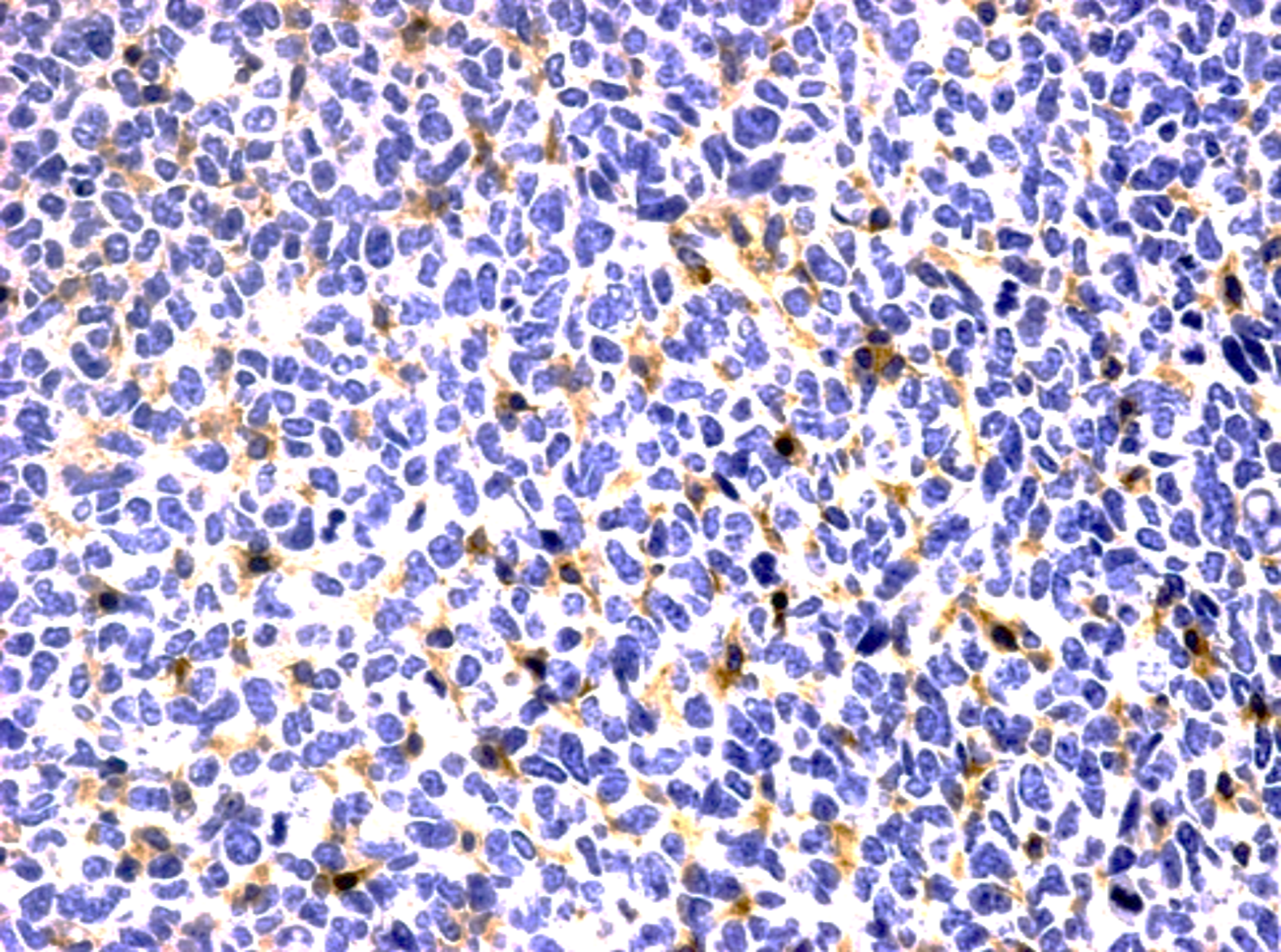

Supplement: Supplementary file 11 — Source Data Fig. 7 [file 44321_2024_51_MOESM11_ESM.zip › Fig-7/7E/CD45 LZ90.tif]

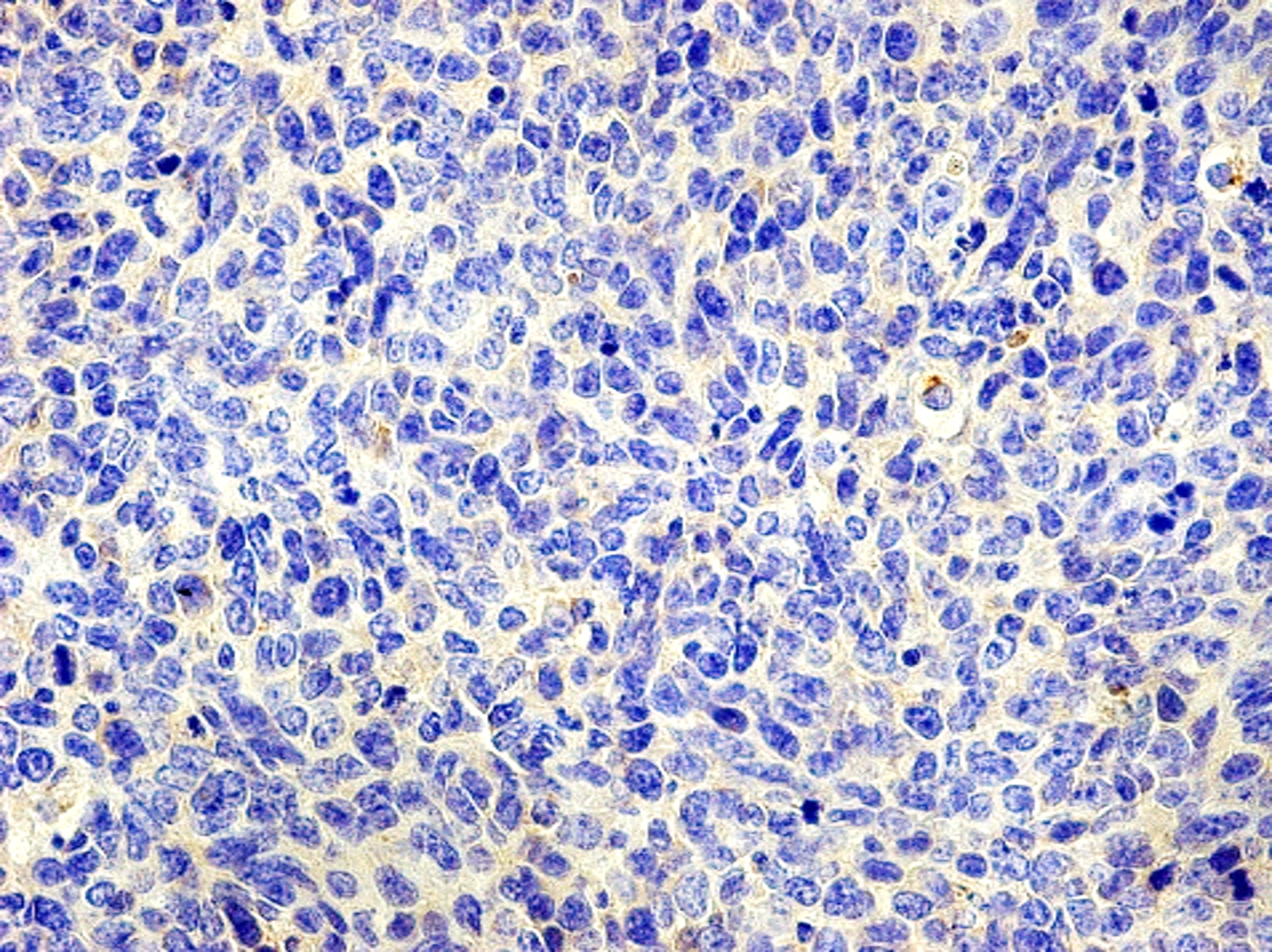

Supplement: Supplementary file 11 — Source Data Fig. 7 [file 44321_2024_51_MOESM11_ESM.zip › Fig-7/7E/CD45 Ctrl.tif]
